# Murine Gammaretrovirus Group G3 Was Not Found in Swedish Patients with Myalgic Encephalomyelitis/Chronic Fatigue Syndrome and Fibromyalgia

**DOI:** 10.1371/journal.pone.0024602

**Published:** 2011-10-12

**Authors:** Amal Elfaitouri, Xingwu Shao, Johan Mattsson Ulfstedt, Shaman Muradrasoli, Agnes Bölin Wiener, Sultan Golbob, Christina Öhrmalm, Michael Matousek, Olof Zachrisson, Carl-Gerhard Gottfries, Jonas Blomberg

**Affiliations:** 1 Section of Clinical Virology, Department of Medical Sciences, University of Uppsala, Uppsala, Sweden; 2 Gottfries Clinic AB, Institute of Neuroscience and Physiology, University of Gothenburg, Gothenburg, Sweden; George Mason University, United States of America

## Abstract

**Background:**

The recent report of gammaretroviruses of probable murine origin in humans, called xenotropic murine retrovirus related virus (XMRV) and human murine leukemia virus related virus (HMRV), necessitated a bioinformatic search for this virus in genomes of the mouse and other vertebrates, and by PCR in humans.

**Results:**

Three major groups of murine endogenous gammaretroviruses were identified. The third group encompassed both exogenous and endogenous Murine Leukemia Viruses (MLVs), and most XMRV/HMRV sequences reported from patients suffering from myalgic encephalomyelitis/chronic fatigue syndrome (ME/CFS). Two sensitive real-time PCRs for this group were developed. The predicted and observed amplification range for these and three published XMRV/HMRV PCRs demonstrated conspicuous differences between some of them, partly explainable by a recombinatorial origin of XMRV. Three reverse transcription real-time PCRs (RTQPCRs), directed against conserved and not overlapping stretches of *env*, *gag* and integrase (INT) sequences of XMRV/HMRV were used on human samples. White blood cells from 78 patients suffering from ME/CFS, of which 30 patients also fulfilled the diagnostic criteria for fibromyalgia (ME/CFS/FM) and in 7 patients with fibromyalgia (FM) only, all from the Gothenburg area of Sweden. As controls we analyzed 168 sera from Uppsala blood donors. We controlled for presence and amplifiability of nucleic acid and for mouse DNA contamination. To score as positive, a sample had to react with several of the XMRV/HMRV PCRs. None of the samples gave PCR reactions which fulfilled the positivity criteria.

**Conclusions:**

XMRV/HMRV like proviruses occur in the third murine gammaretrovirus group, characterized here. PCRs developed by us, and others, approximately cover this group, except for the INT RTQPCR, which is rather strictly XMRV specific. Using such PCRs, XMRV/HMRV could not be detected in PBMC and plasma samples from Swedish patients suffering from ME/CFS/FM, and in sera from Swedish blood donors.

## Introduction

A gammaretrovirus related to the Mouse Leukemia Viruses (MLVs), was 2006 found in a few percent of patients suffering from prostate cancer [1]. It was initially named XMRV, “Xenotropic Murine retrovirus Related Virus”. In 2009 XMRV was also found in patients suffering from ME/CFS [2]. Although the width of the term “XMRV” can be understood in a rather broad way, it is often used in a more restricted sense, as a specific xenotropic gammaretrovirus that was found in humans with these diseases. In 2010, the term XMRV was complemented with “HMRV” (Human retrovirus related to Murine RetroVirus), because gammaretroviral sequences found in ME/CFS were more diverse than just XMRV [3]. It is known that endogenous retroviral sequences (ERVs) highly related to XMRV and HMRV occur in the mouse genome. In fact, XMRV was recently reported to be a recombinant between two MLV-related murine ERVs [4]. However, beyond that, a more exact mapping between the MLV-related sequences found in humans, and the murine ERVs, and indeed the clustering of intact murine gammaretroviral proviruses has not been performed. We will in the following refer to the gammaretroviruses related to murine ERVs which have been reported to occur in humans as “XMRV/HMRV”. It is also unknown if XMRV/HMRV is confined to the mouse genome. Thus, the origin and spread of XMRV/HMRV can be studied bioinformatically in the genomes of the mouse and other vertebrates. In principle, the reports of XMRV/HMRV in humans with and without disease could have far-reaching implications, for the personal life of the patients, for the development of diagnostic methods, for transfusion safety, and for the understanding of other human diseases with a possible retroviral etiology. Reports which verify [3] and do not verify [5], [6] the original ME/CFS report have come. The conflicting results may be due to methodological differences (some were demonstrated here), an uneven geographic distribution of XMRV/HMRVs, or different types of laboratory contamination [4], [7], [8], [9], [10], [11], [12].

MLVs are gammaretroviruses which may be both exogenous (infects between individuals of a similar generation, i.e. horizontally), or endogenous (proviruses integrated into the germ line of mice and were thereby transmitted to the next generation, i.e. vertically). However, a wider group of MLV-like gammaretroviruses (“MLLVs”) [13] have been, and are, spreading among vertebrates. They have repeatedly infected nonmurine vertebrates in the not so distant past. Gibbon apes [14], [15] and koalas [16] have been “invaded” by MLLVs. A similar transspecies gammaretroviral infection of uncertain origin occurred recently in birds [17]. MLLVs include the gammaretroviruses of mediterranean and middle eastern cats [18], and of pigs, although the murine origin of the virus in those species is less certain. In the infected animals, MLLVs are associated with significant disease like encephalitis, malignancy (leukemia and lymphoma), wasting, and immunosuppression. Is the human species now also “invaded” by an MLLV, i.e. XMRV/HMRV?

ME/CFS can be diagnosed according to internationally accepted criteria, see e.g. [19]. It seems to be a rather common disease, maybe amounting to 0.4% of the population [19]. Finding the cause, new diagnostic techniques and, hopefully, a cure, for this often debilitating disease is a high medical priority. ME/CFS borders to the diseases fibromyalgia (FM) and irritable bowel syndrome (IBS). Evidently, there is a great need for confirmation of the reports on XMRV/HMRV in ME/CFS, and in other populations. In view of the recently reported diversity of retroviral sequences in ME/CFS, it is important to establish the detection range of XMRV/HMRV detection methods.

In the present study, we first address murine gammaretroviral diversity. We describe three groups among them. We trace the recombinatorial origin of XMRV using murine endogenous retroviral sequences. From this we predict the ability of our own and of some other commonly used XMRV/HMRV PCRs to detect portions of this murine gammaretroviral spectrum. We report the development of two sensitive PCRs which are broadly targeted to detect murine retroviruses s belonging to group G3. We further apply those two and some previously published XMRV/HMRV detection PCRs to PBMCs from ME/CFS patients and sera from blood donors. We also address the possible occurrence of contamination, either from the PCRs themselves, from synthetic target DNA, or from murine DNA.

## Results

### Gammaretroviruslike proviruses of the genomes of the mouse and some other vertebrates, as represented in the “RetroBank” collection

Of 7656 retroviral sequences detected in the mm8 assembly, 1461 were gammaretroviruslike [20]. Some of the latter (300) were scored higher than 2000 by RetroTector (ReTe) [21]. This meant that they were structurally intact or almost intact. They were all complete proviruses in which very few stop or shift (indel) mutations likely incapacitating the provirus were detected. The 300 proviruses contained 35 which had no such mutations, being “intact” by bioinformatic criteria. The 35 had a less than 0.5% LTR divergence. They are marked with green in the Pol trees shown in Information S1. Thus, the 35 proviruses had hallmarks of being infectious, and also belonged to the most recently integrated murine ERVs.

**Figure 1:**
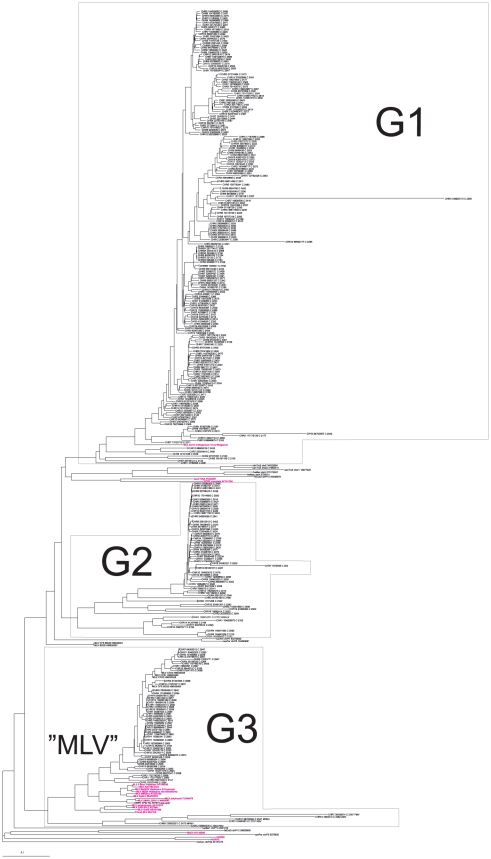
*gag* sequences of 300 high scoring mouse gammaretroviral sequences were aligned together with reference sequences. The tree was rooted with a rabbit sequence. Sequences in red are from ME patients, published in the paper of Lo et al [3]. Two short blood donor (“BD”) sequences from the Lo et al study came out at the beginning of group G2, in most other trees in group G3. “HMRV” corresponds to group G3 in the tree. A higher resolution version is shown in Information S1). (genomic ERV sequences taken from the prototype of RetroBank were named as oryCun = rabbit, cavPor = guinea pig, felCat = Cat, panTro = Chimpanzee or rheMac = Rhesus macaque. Mouse sequences from the mm8 assembly are just shown with their chromosomal location). MLV sequences of known tropism, from GenBank, were also added. The separation of xeno-, modified polytropic and polytropic G3 sequences was not clear in this tree. It was more clear in other trees (Information S1).

The phylogenetic analysis of the 300 proviruses together with related high-scoring gammaretroviruslike proviruses of other vertebrates is presented in Figure 1 (more detailed in Information S1). Briefly, three major groups of high scoring murine gammaretroviral proviruses resulted. The three groups were evident in both *gag* and *pol* nucleotide based trees. The *gag* and *pol* nucleotide based groups contained the same or almost the same members. Group G1 (Gamma 1) contained 188 members, with an average within-group identity of 94%. Ten of them had an ORF in *gag*, *pro*, *pol* and *env*. Members encompassed the MmERV [22] (interpreted by RetroTector from GenBank Id AC005743) sequence. *Mus dunni* ERV (MdERV, AF053745) was highly related. The most similar non-mouse proviruses were the rat Chr17 5186121 and Chr7 31839324 ERVs, and more distantly, Gibbon ape Leukemia Virus (GaLV, PCGGPE) and Koala RetroVirus (KoRV, AF151794) sequences. Group G2 contained 59 members, with an average within-group identity of 85%. Three of them had an ORF in *gag*, *pro*, *pol* and *env*). It contained the GLN murine ERVs of Ribet et al [23]. Outside of the mouse genome, a group of PERV located at chr9 151463024, chr10 71670155, chr12 29233668 and chr4 47233287, respectively, were highly (78–87% identical) related. More distantly related (73–78% identical) were, rat endogenous proviruses on chr9 2586497 and chr7 74285691. Group G3 contained 53 members, with an average within-group identity of 93%. Twentytwo had an ORF in *gag*, *pro*, *pol* and *env*. It encompassed the eco-, xeno-, poly- and modified polytropic endogenous MLVs [24], [25], [26]. The ecotropic exogenous MLVs had only one endogenous G3 counterpart, on chromosome 8 (see Figures 1 and Information S1).

Exogenous ecotropic MLVs were 86–94% identical to the consensus of the endogenous G3 members. They can therefore also be considered to be G3 viruses. The amphotropic MLVs were not represented among the endogenous sequences, but clustered at the base of G3. The Hortulanus endogenous murine provirus (HEMV) [27] was ancestral to the G3 group (not shown here) [13]. The properties of the G1–G3 groups can be further studied in Information S3 and Table S1. The degree of coherence of the groups, consensus sequences and PBS usage are shown in supporting document S3. The relationships between the MLLVs will be further examined in a forthcoming paper [13]. The three major groups were discernible in neighbor joining (NJ) trees resulting from alignment of *gag*, *pol* and *env* nucleotide sequences, and, with 99–100% bootstrap support, in NJ and minimum evolution (ME) trees as well as in maximum likelihood (ML) trees, resulting from Gag, Pol and Env protein alignments, using the MEGA 5 phylogeny package and the ClustalW and MUSCLE alignment programs. They represent three different, evolutionary recent, bursts of gammaretroviral proliferation in the mouse and its immediate progenitors. The third group, highly similar to the retroviruses reported from the human diseases prostate cancer and ME/CFS, contains the highest proportion of bioinformatically intact proviruses. It may thus have the greatest zoonotic potential.

The data set of 300 high scoring murine gammaretroviruslike proviruses, plus reference MLV sequences from GenBank, and related sequences from other species in the “RetroBank” collection [20], was the basis of the bioinformatic prediction of the detection range of the PCRs employed in this work.

### The unique recombinatorial origin of 22Rv1/XMRV studied using murine ERVs

**Figure 2:**
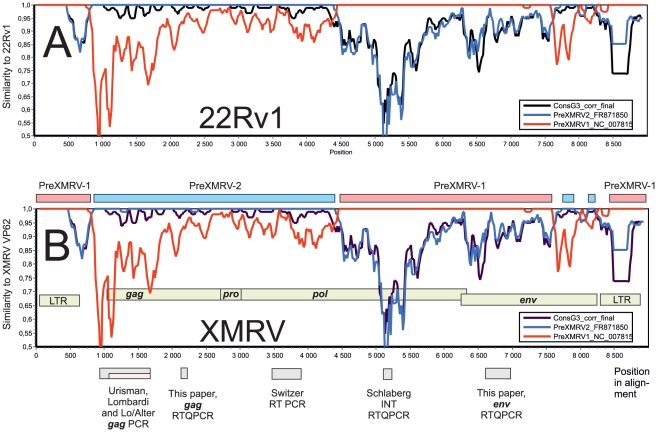
Relation between the XMRV/HMRV PCRs considered in this paper, and the recombinant origin of XMRV/22Rv1. Similarity plots from a Clustal alignment with 22RV1 (Fig. 2A) and XMRV (Fig. 2B) as references, and with PreXMRV-1, PreXMRV-2 and the G3 consensus (from this paper) as queries, were made with Simplot v1.3 (1998, kindly provided by dr S. Ray, dpt of Infectious Diseases, Johns Hopkins University, US) with a window size of 100 and a step size of 20 nucleotides, inclusion of gaps and Kimura transition/transversion scoring. The 22Rv1 virus was 99.9% identical to XMRV over the entire alignment, and yielded an identical similarity plot with the probable ancestors and the G3 consensus. The mosaic nature of XMRV, and its consequences for the detection range of the PCRs considered in this paper, as well as the exact match between 22Rv1 and XMRV, is evident. It is highly unlikely that the same recombination could have occurred by chance independently, outside of the 22Rv1 cell line. Additional material is found in Information S2.

It was recently shown that the XMRV that was found in samples from humans suffering from prostate cancer and ME/CFS most likely is a recombinant between two murine gammaretroviral ERVs, PreXMRV-1 and PreXMRV-2 which arose during passage of the human prostate cancer cell line 22Rv1 in nude mice [4]. We used the mm8 sequences in RetroBank to verify and extend this conclusion. We first searched for the most related sequences to PreXMRV-1, PreXMRV-2, 22Rv1 and XMRV in RetroBank using BlastN, and in the murine genome and nonredundant sequence data sets in GenBank, using MegaBlast. There were no exact counterparts to them. PreXMRV-1 and -2 likely derived from the nude mice which were used to propagate the 22Rv1 cells. These mice have their own variant set of ERVs. However, the four sequences most similar to each of the four were selected, giving 14 XMRV/22Rv1-related sequences, all falling within group G3. XMRV and 22Rv1 were 99.9% identical, and gave the same results in all comparisons with the 14 related sequences. A similarity plot of XMRV/22Rv1 versus PreXMRV-1, PreXMRV-2 and the group G3 consensus sequence (Figure 2a and b) showed that indeed PreXMRV-1 was 99% identical to XMRV/22Rv1 over the middle and the LTRs, while PreXMRV-2 was 99% identical to XMRV/22Rv1 over 3600 nucleotides covering gag, pro and a part of pol, and two shorter regions just before the LTR. No other sequences with such a high degree of similarity to XMRV/22Rv1 were identified in RetroBank or GenBank.

The regional distribution of similarity between PreXMRV-1, PreXMRV-2 and their most similar proviruses from the databases, and the uniqueness of 22Rv1/XMRV among xenotransplantation derived xenotropic viruses is further detailed in Information S2. The region around 5000 (target for the INT RTQPCR mentioned below) seems to be a major dissimilarity region within group G3 genomes (cf. Figure 2). The mosaic origins of 22Rv1/XMRV thus have a bearing on the detection range of the PCRs whose targets are distributed over much of the 22Rv1/XMRV genome (see below and Figure 2).

### Design and evaluation of the *gag* and *env* RTQPCRs

The design considerations, and the basic methodological evaluation, for the *gag* and *env* RTQPCRs are detailed in the Information S4.

### Bioinformatic prediction of detection range for XMRV/HMRV PCRs

**Figure 3:**
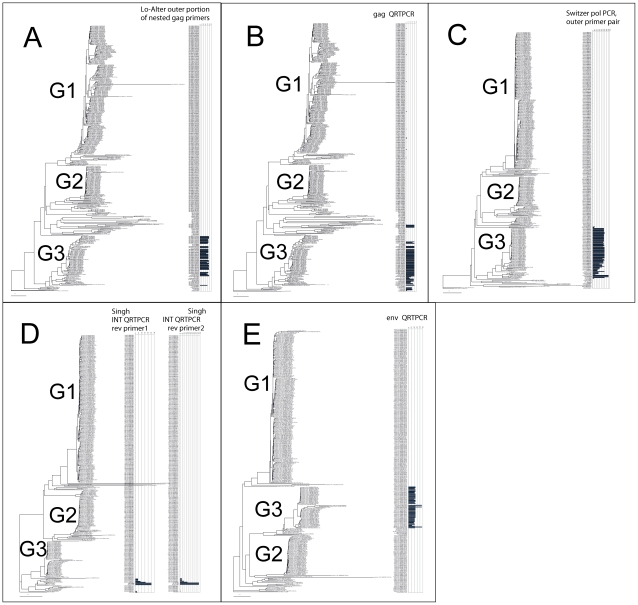
Estimated PCR detection range in an alignment of 300 high-scoring gammaretroviruslike proviruses found by RetroTector in the mm8 assembly. Predictions were mapped onto NJ trees of alignments of *gag*, *pol*, integrase and *env* nucleotide sequences. A PCR detection score (shown as horizontal bars) was calculated by multiplying the fit of primers and probe (if present) to the target sequences for each provirus in the alignment. The degree of fit (match) was estimated using a modified NucZip algorithm [28]. A more complete treatment of this subject will be published separately (Danielsson et al, in preparation). The trees and alignments are further presented, and shown in higher resolution together with trees made with several algorithms, with bootstrap figures, in Information S1. A. A *gag* nt alignment assessed with outer primers of the nested Lo/Alter PCR [3] (HMRV sequences from that paper are shown in red), and B. the same tree with the prediction for the primers and probe of the *gag* RTQPCR presented in this paper. C. A *pol* tree, with the prediction for the outer primer pair of the nested RT-based PCR of Switzer et al [5] is shown. D. A tree based on the 3′ (mainly integrase) portion of *pol*. Predictions based on the Singh RTQPCR, with its two reverse primers [35], are shown. XMRV sequences are shown in red. E. An *env* tree. The prediction of the detection range of the *env* RTQPCR presented in this paper is shown. A higher resolution picture is shown in Information S1.

An approximate prediction of PCR detection range among the aligned sequences was made using a computer program based on the NucZip algorithm [28]. Its output is a detection ability score, shown as horizontal bars for each provirus in an alignment of the 300 intact or nearly intact proviruses in the mm8 assembly. As seen in figure 3, results with both the outer primers of the nested Lo/Alter PCR, and the primers and probe of the *gag* RT QPCR presented here, indicate that it is only the third group (which contains the “traditional” MLV-like proviruses) that can be detected with these PCRs. The predicted detection range of the INT RTQPCR, which uses two different reverse primers, is much more narrow (Figure 3d). The nonendogenous variants of XMRV isolated from humans and human cells (VP62, VP35 and 22Rv1) were indicated to be detectable. However, two highly XMRV-related proviruses on chromosome 1 of C57Black/6J mice (the source of the mm8 assembly) were also indicated to be detectable (chr1 173317855; XMV43 and chr1 172778230; XMV41). The XMV subgroup assignments are from Jern et al [26]. These are the same sequences which were noted to be highly related to PreXMRV-1 in the Information S2. Thus, the C57Black/6J mouse genome contains a few sequences predicted to be detectable with the INT RTQPCR. The reverse transcriptase - directed nested primers of Switzer et al [5] also seem to cover most of group G3. Finally, the predicted detection range of the *env* RTQPCR, with its variation tolerant MegaBeacon probe, covered most of the group G3 murine gammaretroviruses, except for the ecotropic ones (Figure 3 e). All these PCRs, except for the one of Switzer et al [5], were tested in this paper.

### Test of detection range for the *gag* and *env* QPCRs using synthetic targets

The gammaretroviral sequence closest related to XMRV/HMRV in the human genome is HERV-T [29], [30]. We wanted to test how well separated the mouse gammaretroviral group G3 sequences were from HERV-T and from mouse gammaretroviral groups G1 and G2, by systematically letting primer and probe target sequences vary from murine to human sequence. Thus, gammaretroviral *gag* sequences which included XMRV and murine gammaretroviruses from mouse chromosome 4, 10 and 13, as well as HERV-T from human chromosome 11, were aligned. In order to test the detection range of the *gag* RTQPCR synthetic target sequences from aligned portions of these sequences hybrid sequences intermediate between HERV-T and XMRV were made.

**Figure 4:**
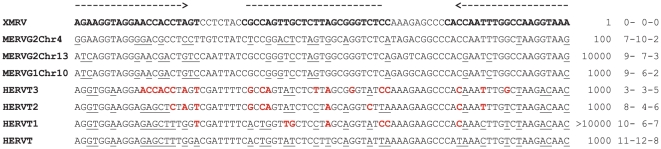
Alignment of endogenous group G1 (chr10) and G2 (chr4 and 13) MERV sequences, HERV-T and artificial hybrid HERV-T/XMRV target sequences used to test the detection range of the *gag* RTQPCR. The HERV-T sequence was gradually changed (red nucleotides) to become more XMRV-like. The detection limit was determined by running PCR with tenfold dilutions giving 1–100 000 molecules per PCR reaction. The lowest positive concentration, in molecules per PCR reaction of the respective constructs, is written to the right of the alignment. The number of mismatches (underlined) between forward primer, probe and reverse primer, respectively, is shown at the far right.

The results (Figure 4) showed that the murine chromosome 4 (belonging to group G2), chromosome 10 (group G1) and 13 (group G2) and HERV-T proviral sequences were only detected at 100–10000 times higher concentrations than the XMRV sequence, respectively. Thus, the predictions shown in figure 3 were approximately corroborated. The broadly targeted *gag* RTQPCR presented in this paper will probably miss murine gammaretroviruses outside of group G3.

**Figure 5:**
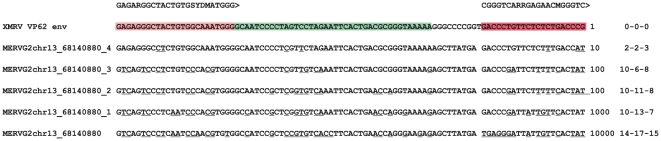
Test of detection range of the *env* RTQPCR using synthetic target sequences. Alignment of primer (mauve and red) and probe (green) target sequences with a synthetic PCR target sequence used to evaluate approximate detection width of the *env* QPCR. The detection limit was determined by running PCR with tenfold dilutions giving 1–100 000 molecules per PCR reaction. The lowest positive dilution in the series, in molecules per PCR reaction of the respective constructs, is written to the right of the alignment. The number of mismatches (underlined) between forward primer, probe and reverse primer, respectively, is shown in the far right. chr13_68140880 is a group G2 MERV.

To make a similar study of the targets of the *env* RTQPCR, a panel of artificial hybrid synthetic *env* sequences, with a gradual transition between chr13 6814088 (belonging to group G2) and XMRV VP62, was synthesized (Figure 5). The five synthetic targets were tested with the *env* RTQPCR. The results (Figure 5) showed that the provirus at chr13 68140880, only could be inefficiently amplified. It was also predicted to be amplifiable neither by the *env* RTQPCR, nor by any other of the evaluated PCRs (Figure 3).

### Evaluation of sensitivity and specificity

**Figure 6:**
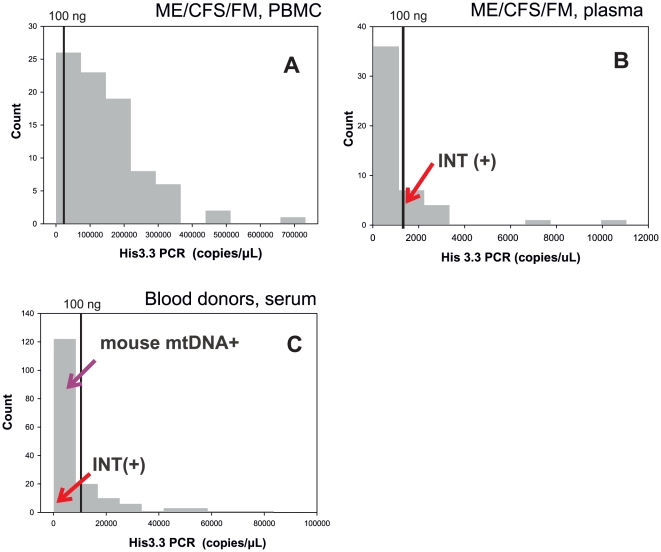
Histograms based on the histone 3.3 RTQPCR results for the three sample sets are shown. **A.** Data from PBMCs of ME/CFS/FM patients. **B.** Data from plasma of ME/CFS/FM/patients. **C.** Data from blood donor sera.

None of the PCRs amplified from human DNA. The *env* and *gag* RTQPCRs had a sensitivity of 1–10 copies of XMRV VP62 plasmid and synthetic target DNA in dilution experiments. The INT RTQPCR did not amplify from two different (Balb/C and C3H) mouse DNA samples. The two xenotropic endogenous sequences at chr1 173317855 and chr1 172778230 of C57Black/6J, predicted to be amplifiable, may not be present in these mice. The *env* and *gag* RTQPCRs, as well as the Lo/Alter primers, amplified from mouse DNA. In several tenfold dilution experiments (not shown), the *env* and *gag* RTQPCRs, and the nested Lo/Alter primers, could detect 0.1 - 0.01 copies of mouse DNA, whereas the non-nested outer Lo/Alter primers could detect around 1 copy of mouse DNA. The nucleic acid extracts of samples from patients and blood donors had a variable nucleic acid content, as estimated by the His3.3 RTQPCR: Blood donor sera contained 0.17–116, ME/CFS/FM PBMC samples 0.06–1017, and ME/CFS/FM plasma 0.53–15.9 ng amplifiable nucleic acid/PCR reaction. Thus, a positive outcome of XMRV/HMRV PCRs could not be expected from all samples due to a low RNA/DNA concentration (Figure 6).

### Results with the three RTQPCRs and human samples

#### *gag* RTQPCR

This PCR yielded no positive samples out of 85 ME/CFS/FM samples from Gothenburg. However, two samples (one from PBMC and one from plasma) reacted weakly initially, but sequencing revealed that this weak signal was due to contamination from the synthetic positive control with an abridged XMRV sequence shown in figure 4. This artificial sequence is highly unlikely to be of natural origin. None of 168 Blood donor samples from Uppsala were positive. However, 11 were initially weakly reactive (3–16 copies per PCR reaction, Ct 38.1–41.3). Sequencing revealed the same artificial sequence as mentioned in the previous section.

All PCR results except for the His3.3 RTQPCR results are summarized in Table 1.

**Table 1:** Summary of PCR results.

Category	Screening RTQPCR	N	Other PCRs	N	Other confirmatory assays
ME+FM patients(49 plasma, 85 PBMC)					
Plasma	INT (+), env −, gag −	1	nested gag PCR −, mtQPCR −, IAP PCR−	1	
Plasma	INT −, env −, gag (+)	1	nested gag PCR −, mtQPCR −, IAP PCR−	1	1 contamination*
Plasma	INT −, env −, gag −	47			
PBMC	INT −, env −, gag (+)	1	nested gag PCR −, mtQPCR −, IAP PCR−	1	No confirmatory sequencing done
PBMC	INT −, env −, gag −	84			
Blood donors(168 sera)					
Serum	INT (+), env −, gag −	1			
Serum	INT −, env −, gag (+)	11	nested gag PCR +, mtQPCR −, IAP PCR −	1	11 contamination*
			nested gag PCR −, mtQPCR +, IAP PCR −		1
			nested gag PCR −, mtQPCR −, IAP PCR−		9
Serum	INT −, env −, gag −	156			

(+) means weak and not repeatable positive.

*Contamination from synthetic target DNA proven by sequencing.

#### *env* RTQPCR

None of the 85 ME/CFS/FM patients from Gothenburg and 168 Blood donor serum samples from Uppsala were positive in the *env* RTQPCR, neither were they weakly reactive.

#### INT RTQPCR

Similarly, none of 168 blood donor serum samples from Uppsala, PBMC and plasma samples from 85 ME/CFS/FM patients in Gothenburg were positive in the INT RTQPCR. However, one out of 168 blood donor serum samples from Uppsala was weakly initially reactive with Ct = 41.92. One plasma sample from the 85 ME/CFS/FM patients from Gothenburg was also weakly initially reactive, with Ct = 41.41. None of these two was repeatable.

### Assessment of amplifiable nucleic acid in the human samples

As seen in figure 6, the histone 3.3 RTQPCR revealed a variable amount of amplifiable nucleic acid in the samples. The 85 ME/CFS/FM PBMC samples (median 197 ng/PCR reaction) contained most amplifiable nucleic acid, followed by the 168 blood donor sera (median 12.3 ng/PCR reaction) and the 49 ME/CFS/FM plasma samples (median 2.5 ng/PCR reaction).

In order to avoid false negative results, a desirable amount of DNA per PCR reaction is 100 ng (DNA from approximately 15000 cells). This limit is plotted in figure 6, using an approximate conversion from amplifiable nucleic acid found by the His3.3 RTQPCR. In the PBMC samples, around 40% were estimated to contain more than 500 ng DNA per PCR reaction. The weakly reactive RTQPCR results in the three sets, with the exception of the gag RTQPCRs which were shown to be due to contamination (Table 1) are shown as red arrows. The sample positive for mouse DNA contamination is shown with a blue arrow.

### Further control PCRs

The sensitivities of the mouse mtDNA QPCR and IAP PCR were tested by using serial 10-fold dilutions of mouse (C3H and Balb/c) DNA. Both PCRs could detect mitochondrial DNA at a quantity corresponding to 0.1–1 genome copy per PCR reaction. There are around 10 000 mitochondrial DNA copies per mouse cell [31]. The mouse genome (mm8 assembly) contains 3361 more or less complete IAP proviruses (data from RetroBank [20]), and probably an at least tenfold higher amount of IAP LTRs. The IAP PCR was targeted to IAP LTRs. Given this high target frequency, these mouse DNA tests must have had a roughly 1000- fold lower sensitivity for mouse DNA than the XMRV/HMRV PCRs (*gag* and *env* RTQPCR, and nested *gag* PCR; see above) which could detect 1–10 copies per PCR reaction. The mitochondrial DNA PCR was positive in one of eleven tested blood donor sera and in none of two tested PBMC samples. The IAP PCR was positive in none of eleven tested blood donor sera and in none of two PBMC samples. Thus, we got evidence for mouse DNA contamination in only 1 of 13 tested samples. Finally, the nested *gag* PCR of Lo et al [3] was not positive in 11 tested blood donor sera and in none of two tested PBMC samples. These control PCRs were used only for the weakly reactive samples from the screening RTQPCRs. However, the eleven weakly *gag* reactive samples were proven to arise from contamination with a synthetic target DNA. The two weakly INT RTQPCR reactive samples were not positive in the mouse DNA control PCRs. Thus, although our single mtDNA positive sample confirmed that mouse DNA contamination can occur in blood samples from humans [8], [9], [10], [12], [32] it did not change our XMRV detection result.

## Discussion

ME/CFS is a common disease [33]. It is often debilitating, but despite several decades of research its clinical manifestations still need to be more studied. All information which can contribute to the understanding of this disease is important.

The retroviruses which we study occur both in exogenous and endogenous form. The nonidentity between the human-derived XMRV and its closest relatives in the mouse genome is 5–7%, according to a BLAST search performed by JB (unpublished). Assuming that mice were the source of XMRV (a likely supposition) this small deviation, and the known high rate of variation in exogenous retroviruses, indicates a rather recent transmission to humans. Alternatively, all XMRV findings are due to contamination from a common source. The design of tests (PCR and serology) for infection with these viruses is highly dependent upon information regarding the possible spectrum of target viral sequences. A valuable resource is the not yet publicly available “RetroBank” [20]. It is based on the computer program RetroTector© [21], [34]. The prototype of RetroBank currently contains c∶a 40 000 retroviral sequences from 30 vertebrate genomes. The availability of this rich sequence source allowed us to evaluate current XMRV/HMRV nucleic acid based tests for expected range of retroviral detection. The result indicated that the *gag* and *env* RTQPCRs described here, as well as the nested *gag* PCR [3] should be able to detect most retroviruses related to MLVs in group G3 in the tree in Figures 1 and 3, i.e. they should have a similar detection range. The detection range of the INT RTQPCR [35] seems to be much more narrow, which fits with the absence of amplification from mouse DNA with this RTQPCR. Nevertheless, the INT RTQPCR did not give a positive result, according to our criteria of repeatability with several different XMRV/HMRV specific PCRs. However, we got two initially weakly reactive results in two samples. The reason for the weakly reactive INT RTQPCR results is unknown. Despite that the nested *gag* PCR [3] should have a similar detection range as both *gag* and *env* RTQPCRs it did not become positive when samples weakly reactive with these RTQPCRs were retested with the nested *gag* PCR. An explanation could be that the nested *gag* PCR was not quite as sensitive as the RTQPCRs, which reached sensitivities of 1–10 target DNA copies, or that retroviral NA was somewhat degraded during subsequent freezing and thawing of the sample. The amplification range predictions indicate that the MLV related retroviral sequences, which are the ones reported in ME patients [2], [3], should be detectable with the *gag* and *env* RTQPCRs, whereas the INT RTQPCRs should be confined to the most XMRV-like targets, and should be less prone to false positivity due to mouse DNA contamination.

It should also be emphasized that a substantial portion of the 300 high scoring murine gammaretroviral proviruses belong to the here defined murine gammaretroviral groups G1 and G2. Both of these contain some proviruses which are completely intact and are strong candidates for being infectious. None of these should be detectable with current PCRs. Thus, we may not have seen the entire range of murine gammaretroviruses with zoonotic potential for humans.

The mouse retroviruses which have been reported to occur in humans are potentially pathogenic. This kind of viruses can give cancer (especially leukemia), encephalitis and immune deficiency in mice and other natural hosts. It is therefore logical to investigate their presence in patients with ME/CFS (encephalitis and immune deficiency) and in cancer (prostate cancer). However, the absence of a link to leukemia in humans is puzzling. Alternatively, XMRV/HMRVs could be passenger viruses without disease consequences. But the situation is interesting and should be followed up. Because of the great variation in results, methodological optimization is a high priority.

Contamination is an omnipresent hazard whenever supersensitive tests are employed. The three screening PCRs used here can detect 1–10 target molecules. There are three possible sources of contamination, i.e. false positivity in the PCRs, which should be considered, PCR amplimers, positive control DNA, and mouse DNA. In the first case, the controls for PCR amplimer contamination were non-template controls (PCR water; 1 to 4 samples per PCR round). None of them were positive. The three RTQPCRs are non-overlapping, and therefore cannot contaminate for each other. In the second case, the control for positive control DNA contamination, e.g. the XMRV VP62 clone, was sequencing. We sequenced amplimers from the few weakly reactive *gag* and INT RTQPCRs. The *gag* RTQPCR amplimers had exactly the sequence of the synthetic positive control DNA for the *gag* RTQPCR, shown in figure 4. It had the sequence of XMRV VP62, but contained a characteristic 10 bp deletion. These weak reactions must have been due to contamination with this artificial DNA. Obviously, the number of non-template controls per PCR round was too small to detect this low frequency and low level of contamination. The sequences from the weakly and not repeatably positive INT RTQPCRs were identical to the target XMRV clone VP62 sequence for this PCR. The clone sequence is identical in many XMRVs. This makes it hard to distinguish true from false positivity due to contamination by sequencing. The third case, contamination with mouse DNA, was tested using the mouse mtDNA PCR and the IAP PCR. Likewise, MLV-like proviruses, high and low scoring, predicted to be detectable with most of the XMRV/HMRV PCRs, but not the INT RTQPCR which is rather strictly XMRV specific, occur in around 500 copies in the mouse genome (JB, data not shown). Therefore, even a slight mouse DNA contamination in samples subjected to XMRV/HMRV PCRs could cause a false positivity. Biologicals may contain much vertebrate DNA, which in its turn contains thousands of ERVs, possibly confounding sensitive and broadly targeted PCRs. Heparin, for example, contains DNA from the source animals (mostly pig and cow). The pig and cow genomes do however not contain proviruses expected to react in the PCRs used in this paper (JB, information from RetroBank [20]). Moreover, patient samples (whole blood) analyzed in this paper were collected in EDTA tubes (PBMCs) or plain glass tubes (sera) and should not contain heparin. In a parallel titration, the sensitivities of the mouse DNA PCRs were lower than that of the *gag* and *env* RTQPCRs. The absence of XMRV/HMRV positive results in our samples indicates that mouse DNA contamination was a small problem in our samples. However, the results of our bioinformatic search for MLV-like RV in vertebrate genomes, plus our experience of this issue, allows a few comments. There are similarities between the XMRV/HMRV and the “Human Retrovirus 5” (HRV5) stories [36]. HRV5 is one of the so-called “rumor viruses” [37]. It turned out to be a rabbit retrotransposon (RERV-H) whose DNA is abundant in rabbit sera [38]. The rabbit genome contains around 700 copies of RERV-H [38]. Any laboratory which handles rabbit sera is at risk of RERV-H contamination. In analogy with this, a low level of mouse DNA could be present in laboratory reagents or in the laboratory environment [8], [9], [10], [12], [32]. However, non-template controls should also be positive then. They were not. A source of mouse DNA in our laboratory is uncertain and unlikely. As shown here, the likelihood of mouse DNA contamination causing reactions in the INT RTQPCR, where we had a few weak reactions, is lower than that of the other RTQPCRs.

### Conclusions

The mouse genome contains three groups of high scoring gammaretroviral proviruses. Some of them may have zoonotic potential. The third group contains the highest proportion of structurally intact proviruses, and is the target of most PCRs used to detect XMRV/HMRV. Two new broadly targeted XMRV/HMRV PCRs were developed. They were employed on samples from ME/CFS patients and blood donors. False reactions due to contamination with synthetic target DNA were encountered. These could be classified as false by sequencing. The few remaining reactions did not fulfill our criteria for positivity, because they were not repeatable. The few weak and uncertain PCR reactivities encountered by us are very different from the high detection frequencies reported by others [2], [39], [40]. It is possible that a higher amount of nucleic acid used per PCR could have given a higher frequency of XMRV/HMRV detection. However, many samples contained levels of nucleic acids similar to those used in other studies [2], [3], [5], [39], [40]. Under the conditions used by us, we could not corroborate that XMRV/HMRV is frequent in Swedish ME/CFS/FM patients and blood donors.

## Methods

### Computer program for identification of conserved portions in a set of aligned sequences

The program ConSort (JB, unpublished) displays the variation and the number of contributing sequences are displayed. ConSort was used for selection of target sequences for primers and probes of the two new XMRV/HMRV-directed real-time PCRs described in this paper.

### Gammaretroviruses of C57BL/6J mice (genome assembly mm8)

This assembly was analyzed with the RetroTector^©^ system (ReTe) [21]. The results were stored in a prototype version of RetroBank^©^ [20]. Gammaretroviruslike proviruses which scored more than 2000 were selected. This yielded 300 proviruses. Examples of the most structurally intact gammaretroviruslike proviruses from the cow, pig, guinea pig, rabbit, tupaia, marmoset, rhesus, baboon, chimpanzee and human genomes were also included as references. Alignments were made using ClustalW [41] version 1.83, and MUSCLE [42], [43]

Phylogenetic trees were either the Neighbour-Joining (NJ) guide trees from ClustalW, or Minimum Evolution (ME) and Maximum Likelihood (ML) trees, with bootstrapping where appropriate, embodied in the MEGA [44] program suite.

### Bioinformatic assessment of amplification range of the QPCRs

The forward and reverse primers, and the probes, were aligned to the *gag* and *env* alignments of the 300 gammaretroviruslike proviruses. The degree of fit between target sequence and primers and probe was calculated using a modified version of the NucZip algorithm [28]. The predicted detection ability (PDA) was calculated using the product of primer scores, and probe score. PDAs were displayed for each member of the aligned sequences, and mapped onto a Neighbor-Joining (NJ) tree of *gag*, integrase and *env* nucleotide sequences targeted by the respective PCR system.

### Real-time PCR systems; *gag* RTQPCR with a minor groove binding probe

The forward primer 5′- AGAAGGTAGGAACCACCTAGT -3′, Reverse-primer 5′-TTTACCTTGGCIAAATTGGTG -3′ (I = inosine), and the Probe: 5′ - 6FAM-CGCCAGTTGCTCTTAGCGGGTCTCC MGB-NFQ-3′, (6FAM, 6-carboxyfluorescein; MGB-NFQ is a minor groove binding tail with nonfluorescent quencher) (Applied Biosystems.Warrington, Cheshire, UK).

The PCR reaction contained 1 µl nucleic acid extract, 5 µl 5xQiagen OneStep RT-PCR Buffer from QIAGEN® OneStep RT-PCR kit (Catalog no. 210212; QIAGEN, Hilden, Germany), 1 µl dNTP mix (containing 10 mM of each dNTP), 300 nM forward and reverse primers, 100 nM MGB-NFQ probe; RNase-free water; and 1 µl QIAGEN OneStep RT-PCR enzyme mix (total volume 25 µl). The subsequent RT step (cDNA synthesis) was performed at 50°C for 30 min, immediately followed by an initial denaturation at 95°C for 15 min. A total of 45 cycles were then performed, each consisting of a denaturation step at 95°C for 10 sec and an annealing-extension step in which the annealing temperature was 50°C for 45 sec and extension at 60°C for 20 sec. *gag*, *env*, INT and His3.3 RTQPCRs as well as the mitochondrial DNA QPCR were performed using the Corbett Research RotorGene Real Time Amplification system (RG 2000; Corbett Research, Mortlake, NSW Australia). The RotorGene™ software version 6.4 (Corbett Research) was used for threshold selection and standard curve interpolation to derive approximate RNA and DNA concentrations relative to DNA standards. Artificial synthetic targets for this and the other PCRs were ordered from Biomers.net, Ulm, Germany.

### *env* RTQPCR with MegaBeacon probe

The forward primer 5′-GAGARGGCTACTGTGSYDMATGGG -3′, Reverse-primer 5′- CGGGTCARRGAGAACMGGGTC -3′, and the XMRV_MegB2 Probe: 5′- CAATCCCCTAGTCCTAGAATTCACTGACGCGGGTAAAAAtaggggattg-3′

The MegaBeacon probe was labeled with the fluorescent reporter dye JOE at the 5′-end and the quenching Dabcyl at the 3′-end position (Eurogentec Seraing, Belgium). The underlined sequence at the 5′ and 3′ ends identifies the arm sequences of the MegaBeacon that is the stem, (10 bp). The 10 nucleotides at the 3′ end (taggggattg) are not complementary to the target (Information S4). The PCR reaction contained 1 µl nucleic acid extract, 12.5 µl 2× RT-PCR Step RT-PCR kit for Probes (BioRad, Sundbyberg, Sweden), 400 nM forward and reverse primers, 200 nM MegaBeacon probe; nuclease-free water; 0.5 µl iScript reverse transcriptase enzyme (total volume 25 µl). The subsequent RT step (cDNA synthesis) was performed at 50°C for 30 min, immediately followed by an initial denaturation at 95°C for 15 min. A total of 45 cycles were then performed, each consisting of a denaturation step at 95°C for 30 sec and an annealing-extension step in which the annealing temperature was 46°C for 45 sec and extension at 72°C for 20 sec. Fluorescence intensity was measured at the end of the extension step in each cycle.

### INT RTQPCR

This was performed as in the paper of Schlaberg et al. [35], with slight modifications. Briefly, the reaction mix consisted of 10 µl 2× iScript RT-PCR reaction mix buffer from iScript™ One-Step RT-PCR kit for Probes (BioRad, Sundbyberg, Sweden), 900 nM XMRV4552F (5′-CGAGAGGCAGCCATGAAGG-3′; *forward primer*), 450 nM XMRV4653R (5′-GAGATCTGTTTCGGTGTAATGGAAA-3′; *reverse primer1*), 450 nM XMRV4673R (5′-CCCAGTTCCCGTAGTCTTTTGAG-3′; *reverse primer2*), 250 nM XMRV 4572MGB probe (5′- 6FAM - AGTTCTAGAAACCTCTACACTC MGB-NFQ -3′) (like for the *gag* RTQPCR, MGB-NFQ is a minor grove binder with nonfluorescent quencher) (Applied Biosystems,Warrington, Cheshire, UK); nuclease-free water; 0.5 µl iScript reverse transcriptase enzyme and 6 µl of template per PCR reaction in a total reaction volume of 20 µl. The subsequent RT step (cDNA synthesis) was performed at 50°C for 30 min, immediately followed by an initial denaturation at 95°C for 15 min. A total of 45 cycles were then performed, each consisting of a denaturation step at 95°C for 10 sec and an annealing step at 60°C for 45 sec.

### XMRV/MLV *gag* Nested PCR

A wide range of XMRV-related mouse viruses (HMRV) were detected in ME patients [3]. In the first round PCR, the PCR reaction contained 1 µl nucleic acid extract (corresponding to up to 1017 ng of total cellular nucleic acid/reaction as judged by the histone 3.3 RTQPCR result) in total reaction volume, 20 µL, 1× Taq buffer, 2.5 mM MgCl2, 0.2 mM dNTP, 0.25 pmol/µL of 419F 5′-ATCAGTTAACCTACCCGAGTCGGAC-3′ primer, 0.25 pmol/µL of 1154R 5′-GCCGCCTCTTCTTCATTGTTCTC-3′ primer (outer primers) (biomers.net, Ulm, Germany), and 0.5 units of AmpliTaq Gold Taq (Applied Biosystems.Foster City, CA). For the second round PCR, the PCR reaction contained 2 µL of round 1 PCR product 1× Taq buffer, 2.5 mM MgCl_2_, 0.2 mM dNTP, 0.25 pmol/µL of NP116 5′-CATGGGACAGACCGTAACTACC-3′ primer or GAG-I-F 5′-TCTCGAGATCATGGGACAGA-3′ primer, 0.25 pmol/µL of NP117 5′-GCAGATCGGGACGGAGGTTG-3′ primer or GAG-I-R 5′-AGAGGGTAAGGGCAGGGTAA-3′ primer, and 0.5 units of.

The cycles for both PCRs were 4 min at 94°C (1 min at 94°C, 1 min at 57°C, 1 min at 72°C)×40 cycles and 10 min at 72°C. Following amplification, 5 µl of PCR-product was separated by electrophoresis on 2% agarose gel. The first-round PCR amplifies a fragment of ∼730 bp from the *gag* gene. The second-round PCR gives a product of 413 bp, using the GAG-I-F and GAG-I-R primers, or a product of 380 bp using the NP116 and NP117 primers.

### Control samples for the XMRV/HMRV PCRs

Positive controls for *gag*, INT and *env* PCRs consisted of nucleic acid extracts from supernatants of the 22Rv1 XMRV-producing prostate cancer cell line (ATCC CRL 2505), and 3 DNA extracts from ME patient PBMC samples found positive for XMRV at the Whittemore-Peterson Institute (WPI) in Reno, Arizona (a kind gift from dr Judy Mikovits). Two of them were weakly positive in the INT RTQPCR, with Ct 41.8 and 37.7, respectively). A positive control for *gag*, *env* and mouse mtDNA QPCRs was DNA extracted from an adult female Balb/c laboratory mouse (Charles River, Denmark) (a gift from dr Ylva Molin, Uppsala; The DNA was obtained from a laboratory mouse reared in Uppsala. The mouse was reared according to the recommendations in “Guide for the Care and Use of Laboratory Animals” of the Swedish National Board for Laboratory Animals (CFN). The rearing and taking of samples from the mouse was approved (C127/4) by the local Ethical Committee for Experimental Use at the Faculty of Medicine, Uppsala University.)

The INT QPCR did not amplify from these mouse DNAs (as reported earlier by dr Singh [35]). Negative (non-template) control was DEPC-Treated Water (Ambion, INC. Austin, USA) and from nuclease-free water included in both the iScript™ One-Step RT-PCR kit for Probes (BioRad, Sundbyberg, Sweden) and the QIAGEN® OneStep RT-PCR kit (QIAGEN, Hilden, Germany).

### Histone 3.3 (His3.3) RTQPCR

A reverse transcription real-time histone 3.3 RNA and DNA QPCR [45] was always run in parallel with the other PCRs to ensure amplifiability of the samples with slight modifications. Briefly, the reaction mix (25 µl) consisted of 1 µl nucleic acid extract, 12.5 µl 2× RT-PCR Step RT-PCR kit for Probes (BioRad), 200 nM histone forward primers 5′-CCTCTACTGGAGGGGTGAAGAA- 3′; 200 nM histone reverse primers 5′- TGCCTCCTGCAAAGCACCGATA- 3′; 200 nM Probe: 6FAM-CTCTGGAAGCGCAGATCTGTTTTAAAGTCCT- MGB-NFQ-3′, (Applied Biosystems.Warrington, Cheshire, UK); nuclease-free water and 0.5 µl iScript reverse transcriptase enzyme. The subsequent RT step (cDNA synthesis) was performed at 50°C for 30 min, immediately followed by an initial denaturation at 95°C for 15 min. A total of 55 cycles were then performed, each consisting of a denaturation step at 95°C for 15 sec and an annealing step at 54°C for 60 sec. Serial dilutions of a histone 3.3 plasmid containing 10^6^–10^0^ copies per PCR reaction were used in the experiment as quantitative standards. Results were expressed as “Histone 3.3 equivalents” (HIEQ). In twenty samples, HIEQ were correlated with DNA concentration determined with a NanoDrop® Spectrophotometer (NanoDrop Technologies inc. Wilmington, USA). On average, 716 HIEQ/uL corresponded to 1 ng/uL of DNA determined by photometry.

### Sequencing of PCR amplimers

PCR amplimers were purified by QIAquick PCR Purification kit (QIAGEN, Hilden, Germany) and cloned using the TOPO™ TA Cloning Kit (Invitrogen, Stockholm, Sweden). The plasmids DNA were isolated by using QIAprep® Spin Miniprep Kit (QIAGEN, Hilden, Germany). The concentration of plasmid DNA was quantified by using the NanoDrop® Spectrophotometer and then using with M13 primers and the fluorescent dye terminator reagents, ABI PRISM® Big Dye™ Terminator v3.1 Cycle Sequencing kit (Applied Biosystems, Foster City, CA) and on an ABI PRISM® 310 genetic analyzer according to the manufacturer's recommendations (Applied Biosystems, Foster City, CA, USA).

### Mouse mitochondrial DNA (mtDNA) QPCR

Mouse DNA contamination in reagents and patient samples is a possibility [9], [10]. This control PCR was used with samples reactive in any of the gammaretrovirus-targeted PCRs. The primer and probe sequences for murine mitochondrial cytochrome oxidase, *cox2*, were kindly provided by dr William Switzer, Centers for Disease Control, Atlanta, USA. The reaction mix consisted of 12.5 µl 2× iScript RT-PCR reaction mix buffer from iScript™ One-Step RT-PCR kit for Probes (BioRad, Sundbyberg, Sweden), 320 nM MCox2-F2 (5′-TTCTACCAGCTGTAATCCTTA-3′), 320 nM MCox2-R1 (5′- GTTTTAGGTCGTTTGTTGGGAT-3′) primers, and 160 nM MCox2-PR1 (5′- FAM-CGTAGCTTCAGTATCATTGGTGCCCTATGGT-BHQ-3′),160 nM MCox2-P1 (5′- FAM-TTGCTCTCCCCTCTCTACGCATTCTA-BHQ -3′) probes (biomers.net, Ulm, Germany); nuclease-free water, 5 µl of template per PCR reaction in a total reaction volume of 25 µl. Thermocycling conditions were 95°C for 10 min, followed by 55 cycles of 95°C for 30 sec and 60°C for 30 sec. Serial 10-fold dilutions of Balb/c and C3H/HeJ 9384 mouse DNA extracts were used to validate the assay.

### PCR assay for Mouse intercisternal A-type particle (IAP) LTR DNA

The primer sequences, which are targeted to the long terminal repeats of the retrotransposon intracisternal type A particle, were kindly provided by dr Oya Cingöz (Tufts, Massachussetts, USA). The PCR reaction was carried out in an total volume of 50 µL containing 1 µl nucleic acid extract, 1×PCR buffer minus Mg, 1.5 mM MgCl_2_, 0.2 mM dNTP mixture, 200 nM of IAP-F 5′-ATAATCTGCGCATGAGCCAAGG -3′ forward primer, 200 nM of IAP-R 5′- AGGAAGAACACCACAGACCAGA -3′ reverse primer (Thermo Fisher Scientific, Ulm, Germany), nuclease-free water and 1 U *Taq* DNA polymerase (Invitrogen, Lidingö, Sweden). Thermocycling conditions were 40 cycles of 95°C for 30 sec, 59°C for 30 sec and 72°C for 30 sec. The expected and observed product sizes were 235–350 bp.

### RNA and DNA extraction

The total nucleic acid was extracted from PBMCs of EDTA blood of ME/CFS/FM patients and sera from blood donors as described by the manufacturer (EasyMag®, bioMérieux, Boxtel, Netherlands). The samples were eluted in 60 µl and stored at −70°C. For samples from ME/CFS patients from Gothenburg, whole blood samples were obtained in CPT tubes (Becton Dickinson, Stockholm, Sweden), and centrifuged as specified by the manufacturer, at 1700 g for 20 min at room temperature. The PBMC fraction (1 ml) was then taken, and 500 µl of it was used for nucleic acid extraction with the EasyMag. Plasma from 49 out of the 85 ME/CFS/FM patients was analyzed. Two hundred µl of it were used for nucleic acid extraction with the EasyMag.

### Efforts to reduce the likelihood of PCR contamination

To avoid false positive results due to DNA or RNA contamination filtered pipette tips, PCR hoods with ultraviolet light and separate rooms for PCR preparation and product analysis were used. One to four negative (non-template) controls were also included in every experiment. To detect any mouse DNA contamination of the extraction reagents, 300 µl of NucliSens® Lysis Buffer was blindly extracted in eight samples. They came out negative in the *gag* RTQPCR and the mouse DNA PCR.

### Clinical samples

The 85 patients included 48 patients with the diagnoses ME/CFS according to the Canadian criteria [46] and 30 patients with both ME/CFS and FM diagnosis. Seven patients only fulfilling the criteria for FM were also included. The FM diagnosis was according to ACR classification [47]. IBS was diagnosed in 40% of the total group of 85 patients with no significant difference in the subgroups. All patients were rated by the FibroFatigue scale [48]. The mean score was 41±9 points indicating moderate to severe degree of disorder. The total variance of the scale is 0–72. Diagnosis was made by three doctors, all M.D. and PhD, well trained in the use of the rating scale and in the diagnosis of the disorders. RNA and DNA were extracted from peripheral blood mononuclear cell (PBMC) of the 85 ME/CFS/FM patients. RNA and DNA were also extracted from plasma of 49 ME/CFS/FM patients.

All patients from the Gothenburg study gave written consent according to a permit from the Ethical Committee of University of Gothenburg (Dnr 680-09), which allowed the samples reported here to be taken.

### Blood donor samples

Sera from 168 consecutive anonymous blood donors were obtained from the blood bank at Uppsala Academic Hospital, Sweden.

The blood donors gave written consent to the use of their serum for analysis of blood-borne viruses according to the routine of the Academic Hospital in Uppsala, and a general permit for this purpose from the Ethical Committee of the Medical Faculty of the Uppsala University (2004).

### Criteria for XMRV/HMRV PCR positivity

Samples were interpreted as “positive” if repeatable signals with at least two different XMRV/HMRV PCRs were obtained. Samples were interpreted as “weakly reactive” if they were reactive only once in one of the three screening RTQPCRs.

## Supporting Information

Table S1 **Detailed list of the members of the G1–G3 groups.**
(PDF)

Information S1 **Phylogenetic trees supporting the G1–G3 groups, and the G3 subgroups (poly-, modified poly- and xenotropic), as well as ecotropic, MERVs.** Relationship to other MLV-related gammaretroviruses.
(PDF)

Information S2 **Xenotropic cell culture contaminating retroviruses and the uniqueness of 22RV1/XMRV.**
(DOC)

Information S3 **The G1–G3 groups. Properties and consensus sequences.**
(DOC)

Information S4 **Development of the** ***gag*** **and** ***env*** **RTQPCR; Evolutionary conservation of target sequences.**
(PDF)
