# Supplementary material for: Murine Gammaretrovirus Group G3 Was Not Found in Swedish Patients with Myalgic Encephalomyelitis/Chronic Fatigue Syndrome and Fibromyalgia
Source: PLoS One. 2011 Oct 12;6(10):e24602. doi: 10.1371/journal.pone.0024602 (PMC3192035; doi:10.1371/journal.pone.0024602)
Supplement: Table S1 — Detailed list of the members of the G1–G3 groups. (PDF) [file pone.0024602.s001.pdf]

Table S4. The 300 murine proviruses of groups G1-G3.

The most intact proviruses were selected from the *Mus musculus* (C57Black/6J) genome assembly mm8 portion of a RetroBank prototype by selecting for a RetroTector score of 2000 (essentially complete proviruses, with no or a few frameshifts or stops) and genus gammaretrovirus (estimated by RetroTector). 306 proviruses fulfilled these criteria. Of them, six were duplicates. RetroTector may produce duplicates when alternative proviral interpretations occur. The lowest scoring of each duplicate was removed, giving 300 proviruses. Three major branches were evident in all phylogenies. They were named G1, G2 and G3 (for gamma 1, gamma 2 and gamma 3). Some of their properties are listed below. Although some exogenous gammaretroviruses cluster with the groups (2-3 rat endogenous proviruses cluster with G2 and many ecotropic exogenous MLVs cluster with G3), only endogenous proviruses from assembly mm8 are shown below. The envelopes of a few G1 and G2 proviruses cluster differently from the rest of those proviruses, probably due to recombination. The subject cannot be treated extensively here.

The columns are:

1. Group: The allocation of groups G1-G3, based on *gag* and *pol* Clustal alignments and phylogenetic trees based on them.
2. Jern subgroups. These are from Jern et al, Plos Genetics 2007. We used BLAT at the UCSC site to search the most highly similar sequence in the mm8 assembly. Not all G3 proviruses could be given a Jern subgroup assignment, for unknown reasons. Several of the concatenated gag/pro/pol/env sequences were not sufficiently different from each other, and yielded ambiguous calls. This uncertainty is reflected in the column.
3. THIS, the G3 subgroup inferred from the clustering in gag, pol and env based trees in this paper.
4. Chromosome in mm8.
5. Chainstart, the start position of the provirus. The 5’ end is always given as the start.
6. Chainend, the end position of the provirus. The 3´end is always given as the end.
7. Sense, which can be the primary (P) or secondary (S) strand. The primary strand is the one used for numbering the chromosome in question.
8. Score, the score calculated by RetroTector©. It is a rough representation of proviral completeness.
9. Genus, in this case always C (gammaretrovirus-like).
10. GagOrf, the sum of frameshifts and stops in the *gag* gene. -1 means that the *gag* gene could not be interpreted.
11. ProOrf, annotated in the same way as GagOrf.
12. PolOrf, annotated in the same way as GagOrf.
13. EnvOrf, annotated in the same way as GagOrf.
14. Estimated PBS. Upper case means “good fit to a PBS sequence”, lower case means “moderate fit to a PBS sequence”. All PBS entries were checked against the tRNAdb in Leipzig.
15. The PBS sequence.
16. LTRdivergence, the % divergence between the two LTRs, a rough measure of time since integration.

GROUP	JERN	THIS	CHR	CHAINSTART	CHAINEND	STRAND	SCORE	GENUS	GAGORF	PROORF	POLORF	ENVORF	PBS	PBSSEQ	LTRDIV, %
G1			12	7167658	7177389	P	2286	C	0	1	2	1		gatgattgtcagcagctt	7,097
G1			19	13997386	13990627	S	2025	C	2	0	9	-1	G	tggtgcattggccgggaa	0,879
G1			5	76673772	76682636	P	2226	C	2	0	2	1	G	tggtgcattggccgggaa	0,701
G1			5R	2907883	2899019	S	2222	C	2	0	2	1	G	tggtgcattggccgggaa	0,701
G1			2	84699377	84708075	P	2410	C	0	0	1	2	G	tggtgcattggccgggaa	0,255
G1			6	10346803	10355554	P	2533	C	0	0	1	0	G	tggtgcattggccgggaa	0,816
G1			5	6489438	6498284	P	2503	C	0	0	2	2	G	tggtgcattggccgggaa	0,704
G1			8	75759184	75767847	P	2254	C	0	0	1	2	G	tggtgcattggccgggaa	0,514
G1			2	68347295	68356977	P	2541	C	0	0	1	1	G	tggtgcattggccgggaa	0
G1			18	42601670	42610283	P	2372	C	2	0	2	3	G	tggtgcattggccgggaa	0,255
G1			11	10344713	10335993	S	2403	C	0	1	0	0	G	tggtgcattggccgggaa	1,106
G1			16	19516342	19507962	S	2262	C	0	0	2	3	G	tggtgcatgggccgggaa	0,664
G1			2	181848717	181857413	P	2572	C	0	0	0	0	G	tggtgcattggccgggaa	0,228
G1			8	20181859	20171871	S	2440	C	0	0	7	1		tcgtgggtttgagtccca	0,141
G1			7	56788381	56798130	P	2389	C	0	0	2	2	G	tggtgcattggctgggaa	0,186
G1			X	69002389	68993727	S	2551	C	0	0	0	0	G	tggtgcattggccgggaa	0,463
G1			17	32782428	32791114	P	2363	C	2	0	6	2	G	tggtgcattggccgggaa	0,847
G1			4	40848866	40840270	S	2446	C	0	0	1	0	G	tggtgcattggccgggaa	0,3
G1			5	58614499	58605862	S	2511	C	2	0	1	0	G	tggtgcattggccgggaa	0,221
G1			6	129778341	129769867	S	2480	C	0	0	1	1	G	tggtgcattggccgggaa	0
G1			9	6804768	6813543	P	2496	C	1	0	0	1	P	tgggggctcgtccgggat	1,444
G1			9	6804768	6813543	P	2483	C	1	0	0	1	P	tgggggctcgtccgggat	1,444
G1			18	6355349	6346758	S	2256	C	0	1	2	0	P	tgggggctcgtccgggat	1,282
G1			6	9818958	9810806	S	2151	C	1	0	2	0	s	tgggggctcggtccggga	0,362
G1			11	121136108	121127122	S	2337	C	1	2	0	0	P	tgggggctcgtccgggat	1,773
G1			13	68825110	68836983	P	2358	C	1	0	2	0		gccctattgcgagatcgt	6,356
G1			7	16826508	16817957	S	2416	C	1	0	1	0	P	tgggggctcgtccgggat	0,348
G1			YR	13247086	13255724	P	2327	C	1	0	3	2	P	tgggggctcgtccgggat	1,677
G1			YR	3831942	3840414	P	2328	C	1	0	3	2	P	tgggggctcgtccgggat	3,859
G1			19	29751613	29760358	P	2455	C	2	0	2	1	P	tgggggctcgtccgggat	0,899
G1			4	31158729	31167425	P	2335	C	0	0	1	0	s	tgggggctcggtccggga	0,906
G1			X	36617602	36608866	S	2245	C	1	0	2	1	s	tgggggctcggtccggga	1,576
G1			6	13410749	13402057	S	2225	C	1	0	1	0	s	tgggggctcggtccggga	0,907
G1			9	19707318	19715654	P	2295	C	0	0	2	1	P	tgggggctcgtccgggat	1,026
G1			17	18094247	18085592	S	2169	C	0	0	0	1	s	tgggggctcggtccggga	0,375
G1			15	32809701	32824366	P	2510	C	0	0	3	1		tgaaaaaatgatgagact	4,112
G1			X	28089696	28081149	S	2529	C	0	1	2	3	P	tgggggctcgtccgggat	1,966
G1			X	29493445	29484899	S	2458	C	0	1	2	4	P	tgggggctcgtccgggat	1,966
G1			X	31327254	31335800	P	2438	C	0	1	2	4	P	tgggggctcgtccgggat	1,966
G1			X	26939449	26930902	S	2499	C	0	1	2	3	P	tgggggctcgtccgggat	1,966
G1			X	26583153	26574602	S	2502	C	0	1	2	3	P	tgggggctcgtccgggat	2,247
G1			X	27741119	27732569	S	2510	C	0	1	2	3	P	tgggggctcgtccgggat	2,247
G1			X	28668814	28677360	P	2535	C	0	1	2	5	P	tgggggctcgtccgggat	2,528
G1			12	20963947	20955602	S	2266	C	1	0	4	0	P	tgggggctcgtccgggat	0,602
G1			3	96458134	96449462	S	2321	C	1	0	1	0	s	tgggggctcggtctggga	2,511
G1			5	144029052	144037580	P	2498	C	1	0	1	0	G	tggtgcattggccgggaa	0
G1			6	104192593	104184002	S	2431	C	3	1	2	0	G	tggtgcattggccgggaa	0,322
G1			18	86334894	86326313	S	2275	C	0	0	1	0	G	tggtgcattggccaggaa	0,643
G1			13	3180552	3171969	S	2400	C	2	0	0	1	G	tggtgcattggctgggaa	0
G1			4	142945865	142954456	P	2399	C	0	0	1	0	G	tggtgcattggccggaaa	0,643
G1			1	87307992	87299408	S	2471	C	1	1	0	0	G	tggtgcattggccgggaa	0,643
G1			7	42172126	42161853	S	2451	C	2	0	3	0	G	tggtgcattggccgggaa	0,322
G1			9	4855377	4846794	S	2480	C	1	0	0	1	G	tggtgcattggctgggaa	0,965
G1			X	4473889	4465301	S	2519	C	1	0	1	1	G	tggtgcattggccgggaa	1,608
G1			7	10085860	10077270	S	2245	C	1	0	1	0	G	tggtgcattggctgggaa	0,322
G1			12	69841080	69832546	S	2492	C	0	0	0	0	G	tggtgcattggccgggaa	0
G1			7	20421984	20413392	S	2420	C	0	0	0	0	G	tggtgcattggccgggaa	0,958
G1			4	39044302	39052894	P	2576	C	1	0	2	0	G	tggtgcattggccgggaa	0,319
G1			9	17870138	17878692	P	2390	C	1	0	1	1	g	tggtgcattggccggaac	0
G1			5R	2061443	2070048	P	2098	C	1	1	3	1	G	tggtgcattggccgggaa	0
G1			5R	353440	344834	S	2084	C	1	1	3	1	G	tggtgcattggccgggaa	0
G1			5	94676896	94668303	S	2218	C	1	0	1	1	G	tggtgcattggccgggaa	0
G1			6	19594284	19602882	P	2520	C	1	0	2	0	G	tggtgcattggccgggaa	0
G1			8	116462276	116446820	S	2500	C	0	0	2	0	G	tggtgcattggccgggaa	0,321
G1			12	24421810	24430369	P	2476	C	0	0	1	1	G	tggtgcattggccgggaa	0,656
G1			7	106162675	106171227	P	2568	C	0	0	2	3	G	tggtgcattggccgggaa	0,656
G1			X	85272635	85264080	S	2458	C	0	0	2	0	G	tggtgcattggccgggaa	0,328
G1			8	28769833	28778387	P	2443	C	1	0	0	1	G	tggtgcgttggccgggaa	0
G1			13	20666097	20657536	S	2518	C	0	0	1	1	G	tggtgcattggccgggaa	0,656
G1			12	23063728	23055192	S	2552	C	2	0	0	0	G	tggtgcattggccgggaa	0,328
G1			15	49007539	48999023	S	2505	C	3	0	1	0	G	tggtgcattggccgagaa	0,656
G1			1	197059344	197050771	S	2547	C	0	0	0	0	G	tggtgcattggccgggaa	0
G1			7	19466915	19475429	P	2431	C	0	0	2	0	G	tggtgcattggccgggaa	0,639
G1			7	22012805	22021319	P	2444	C	0	0	2	0	G	tggtgcattggccgggaa	0,639
G1			X	22753149	22744591	S	2567	C	1	0	1	2	G	tggtgcattggccgggaa	0
G1			11	75181662	75173122	S	2279	C	1	0	4	4	G	tggtgcattggccgggaa	1,286
G1			6	9246408	9254960	P	2574	C	1	0	3	2	G	tggtgcattggccgggaa	0,643
G1			10	3947641	3939089	S	2476	C	0	0	1	0	G	tggtgcattggctgggaa	0,321
G1			7	27736610	27728060	S	2361	C	1	0	3	0	G	tggtgcattggccgggaa	0
G1			12	23639259	23647879	P	2450	C	1	0	0	2	G	tggtgcattggccgggaa	0,637
G1			12	24232686	24224140	S	2523	C	0	0	0	2	G	tggtgcattggccgggaa	0,637
G1			5	76663911	76672491	P	2130	C	2	1	2	0	P	tgggggctcgtccgggat	0
G1			5R	2917745	2909164	S	2096	C	2	1	3	0	P	tgggggctcgtccgggat	0
G1			5R	2051415	2059994	P	2056	C	3	1	2	0	P	tgggggctcgtccgggat	0,324
G1			5R	363468	354889	S	2109	C	3	1	2	0	P	tgggggctcgtccgggat	0,324
G1			5	95293762	95302351	P	2134	C	1	1	2	1	P	tgggggctcgtccgggat	7,541
G1			5R	23133	14545	S	2172	C	1	1	3	1	P	tgggggctcgtccgggat	7,541
G1			5R	698499	707087	P	2160	C	1	1	3	1	P	tgggggctcgtccgggat	7,541
G1			5	94686917	94678345	S	2338	C	2	0	2	2	P	tgggggctcgtccgggat	0,647
G1			5R	1005041	1013599	P	2166	C	1	1	0	1	P	tgggggctcgtccgggat	7,018
G1			5	95611246	95619819	P	2244	C	2	1	2	1	P	tgggggctcgtccgggat	8,525

G1	7	32382271	32373741	S	2428	C	3	1	1	1	P	tgggggctcgtccgggat	1,544
G1	7	32609167	32600637	S	2405	C	3	1	1	1	P	tgggggctcgtccgggat	1,544
G1	17	58525679	58534201	P	2277	C	1	0	4	1	P	tgggggctcgtctgggat	0,386
G1	6	59821337	59812831	S	2342	C	2	0	1	1	p	tgggggctcatccgggat	0,772
G1	13	68413662	68422165	P	2416	C	2	0	3	0	P	tgggggctcgtccgggat	0,388
G1	X	25720491	25711975	S	2091	C	0	0	4	1		tgggtccgcggctgtccc	1,931
G1	4	143392767	143401269	P	2195	C	1	0	4	2	P	tgggggctcgtccgggat	1,556
G1	10	47444316	47435762	S	2180	C	0	0	1	1		tggtctcggtgtcttctt	1,163
G1	X	122485908	122494467	P	2422	C	0	0	0	0	P	tgggggctcgtccgggat	0,388
G1	10	4266211	4257656	S	2384	C	1	0	1	1	P	tgggggctcgtccgggaa	0,974
G1	3	3249466	3241071	S	2366	C	0	0	0	0	P	tgggggctcgtccgggat	0
G1	6	10870226	10878802	P	2075	C	1	0	3	0	P	tgggagcttgtccgggat	1,623
G1	7	31649885	31641310	S	2253	C	1	0	1	0	P	tgggggctcgtccgggat	0,977
G1	11	47262836	47278499	P	2429	C	1	0	2	0	P	tgggggctcgtccgggat	4,531
G1	19	9616180	9624756	P	2432	C	0	0	1	1	P	tgggggctcgtccgggat	0
G1	10	55324861	55316286	S	2409	C	0	0	2	4	P	tgggggctcgtccgggat	0,325
G1	13	112849620	112858177	P	2465	C	3	0	0	0	P	tgggggctcgtccgggat	0,325
G1	7	42296817	42288241	S	2197	C	1	0	1	1	P	tgggggctcgtccgggat	0,325
G1	16	3107315	3098757	S	2157	C	2	0	1	1	P	tgggggctcgtccgggat	0,984
G1	16	3275036	3283649	P	2215	C	1	0	1	3	p	tgggggcttgtccgggat	0,984
G1	14	52643653	52652217	P	2319	C	0	0	0	1	P	tgggggctcgtccaggat	0,326
G1	2	41034625	41043182	P	2320	C	0	0	1	0	P	tgggggctcgtccgggat	0
G1	5	9854828	9863388	P	2350	C	1	0	2	1	P	tgggggctcgtccgggat	2,303
G1	19	40015556	40024100	P	2356	C	0	0	3	2	P	tgggggctcgtctgggat	0,656
G1	18	74384552	74393074	P	2441	C	0	0	2	2	P	tgggggctcgtccgggat	0,325
G1	4	90367080	90375191	P	2459	C	3	0	2	0	P	tgggggctcgtccgggat	0,647
G1	12	19930599	19922104	S	2025	C	0	0	4	0	p	ttggggctcgtccgggat	1,361
G1	1	100117419	100125800	P	2325	C	0	0	2	2	P	tgggggctcgtccaggat	0,733
G1	3	16432285	16423863	S	2375	C	0	0	0	1	P	tgggggctcgtccgggat	0,352
G1	9	6703246	6694842	S	2361	C	0	0	3	0	P	tgggggctcgtccgggat	0
G1	YR	14435559	14427157	S	2156	C	0	0	1	0	P	tgggtgctcgtccgggat	2,606
G1	17	20451942	20460372	P	2302	C	2	0	2	2	P	tgggggctcgtctgggat	1,316
G1	3	67513064	67504620	S	2300	C	4	0	0	0	P	tgggggctcgtccgggat	0
G1	8	76141904	76150343	P	2348	C	0	0	0	0	P	tgggggctcgtccgggat	0,658
G1	1	142780526	142788957	P	2475	C	1	0	2	0	P	tgggggctcgtctgggat	0,329
G1	9	25914164	25906750	S	2354	C	0	0	3	1	P	tgggggctcgtccgggat	0
G1	14	8217491	8209046	S	2380	C	1	0	2	1	P	tggaggctcgtccgggat	0,326
G1	2	55605859	55597411	S	2430	C	1	0	2	0	P	tgggggctcgtccgggat	0,977
G1	X	45216113	45207676	S	2421	C	2	0	3	0	P	tgggggctcgtccgggat	0,329
G1	X	51971219	51979669	P	2230	C	2	1	3	2	p	tgggggctcttccgggat	0,326
G1	9	3947717	3939274	S	2431	C	0	0	1	0	P	tgggggctcgtccgggat	0,658
G1	2	138262497	138253902	S	2403	C	0	0	1	1	P	tgggggctcgtccgggat	0,327
G1	13	35021135	35012682	S	2294	C	0	0	3	2	P	tgggggctcgtccgggat	0
G1	3	11123338	11131784	P	2314	C	1	0	1	0	P	tgggggctcgtccggaat	0,329
G1	7	85330013	85338444	P	2396	C	1	0	1	0	P	tgggggctcgtccgggat	0,987
G1	7	20210541	20202039	S	2155	C	2	0	1	0	P	tggggactcgtccgggat	1,948
G1	7	22620924	22629418	P	2003	C	2	0	2	1	P	tggggactcgtccgggat	1,299
G1	4	123354415	123345896	S	2334	C	0	0	2	0	G	tggtgcattggccgggaa	0,327
G1	13	50732587	50724092	S	2452	C	1	0	3	1	P	tgggggctcgtccgggat	0,649
G1	8	4959420	4967904	P	2074	C	1	0	2	1	P	tgggggctcgtccaggat	0
G1	X	161151110	161159604	P	2346	C	0	0	0	0	P	tgggggctcgtccgggat	0,974
G1	3	10857898	10866412	P	2158	C	0	0	2	1	P	tgggggctcgtccgggat	0,325
G1	1	27674279	27682528	P	2288	C	1	0	3	1	P	tgggggctcgtccgggat	0,833
G1	10	78270986	78262676	S	2308	C	0	0	1	0	P	tgggggctcgtccaggat	0,588
G1	2	25549391	25541036	S	2052	C	1	0	6	1	P	tgggggctcgtccgggat	0
G1	7	20210495	20202042	S	2128	C	2	0	1	0	P	tggggactcgtccgggat	2,317
G1	2	67930798	67939218	P	2267	C	2	0	4	0	P	tgggggctcgtccgggat	0
G1	12	44540601	44549025	P	2271	C	2	0	1	1	P	tgggggctcgtccgggat	0
G1	1	119978260	119969841	S	2348	C	0	1	1	1	P	tgggggctcgtccgggat	0,775
G1	19	13109265	13117705	P	2405	C	1	0	3	0	P	tgggggctcgtccgggat	0
G1	13	78107530	78099089	S	2275	C	1	0	0	0	P	tgggggctcgtccgggat	0,388
G1	15	15004671	14996247	S	2321	C	1	0	3	2	P	tgggggctcgtccgggat	0,4
G1	16	61201576	61193150	S	2398	C	0	0	1	0	P	tgggggctcgtccgggat	1,261
G1	12	77663152	77654702	S	2400	C	0	0	2	1	P	tgggggctcgtccgggat	0
G1	4	143285616	143294099	P	2223	C	0	0	2	2	P	tgggggctcgtccgggat	0
G1	7	62535812	62550466	P	2471	C	3	0	2	1	P	tgggggctcgtccgggat	0,388
G1	3	27214964	27206590	S	2480	C	0	0	1	0	G	tggtgcattggccgggaa	0
G1	14	37020689	37029018	P	2481	C	2	0	1	1	G	tggtgcattggccgggaa	0
G1	5	10627682	10635871	P	2477	C	0	0	2	0	G	tggtgcattggccgggaa	0
G1	X	116205149	116196774	S	2363	C	0	0	2	0	G	tggtgcattggccgggaa	0,98
G1	1	147966500	147958131	S	2505	C	0	1	0	0	G	tggtgcgttggccgggaa	0,99
G1	X	75140573	75148991	P	2573	C	2	0	2	2	G	tggtgcattggccgggaa	0
G1	X	110244634	110253003	P	2372	C	0	0	4	0	G	tggtgcattggccgggaa	0
G1	1	85024403	85016029	S	2443	C	1	1	0	2	G	tggtgcattggccgggaa	0
G1	9	7407328	7415688	P	2543	C	1	0	1	2	G	tggtgcattggccgggaa	0
G1	X	3017764	3009388	S	2183	C	1	0	3	1	G	tggtgcattggccgggaa	1
G1	X	3107092	3115469	P	2505	C	2	0	1	1	G	tggtgcattggccgggaa	3
G1	7	103168275	103175762	P	2419	C	0	0	1	3	G	tggtgcattggccgggaa	0,565
G1	9	7407606	7414943	P	2253	C	1	0	1	-1	G	tggtgcattggccgggaa	
G1	X	3107370	3114722	P	2210	C	2	0	1	1	G	tggtgcattggccgggaa	
G1	X	117175129	117167525	S	2177	C	0	0	0	1			24,176
G1	13	86668875	86656476	S	2163	C	2	0	2	2	p	tggaggctcgtccaggat	
G1	7	13722716	13714420	S	2371	C	2	0	2	0	P	tgggggctcgtccgggat	1,869
G1	X	73147008	73155387	P	2584	C	0	0	3	0	G	tggtgcattggccgggaa	0
G1	13	68917752	68909459	S	2618	C	1	0	0	0	G	tggtgcattggccgggaa	0
G1	4	143351516	143359835	P	2530	C	0	0	0	0	G	tggtgcattggccgggaa	0
G1	14	39042987	39034705	S	2287	C	1	0	2	0	G	tggtgcattggccaggaa	2
G1	9	90737869	90729581	S	2531	C	0	0	2	0	G	tggtgcattggccgggaa	2,941
G1	12	52231624	52240129	P	2467	C	1	0	1	0	G	tggtgcattggccgggaa	5,755
G1	13	68562177	68570978	P	2285	C	0	0	2	1	G	tggtgcattggccgggaa	13,158
G1	12	88605018	88613305	P	2422	C	1	0	2	1	P	tgggggctcgtccgggat	0
G1	7	23024544	23016319	S	2465	C	0	0	1	1	P	tgggggctcgtccgggat	3,846

G1			9	12101539	12091225	S	2049	C	2	0	8	1	P	tgggggctcgtccgggat	2,305
G1			2	27317207	27325386	P	2123	C	4	1	5	-1	G	tggtgcattggccgggaa	6,759
G1			5	105161106	105169841	P	2105	C	6	0	6	1	G	tggtgcattggctgggaa	1,893
G1			7	33735121	33743847	P	2134	C	3	0	6	6	P	tgggggctcgtccgtgat	0,787
G1			11	9790554	9782416	S	2051	C	5	1	12	2	G	tggtgcattggccgggaa	7,113
G2			4	34538284	34530059	S	2471	C	2	0	1	1	Q	tggaggtcccaccgagat	0,418
G2			7	52202011	52193788	S	2486	C	1	0	0	1	Q	tggaggtcccaccgagat	0
G2			16	55879954	55888171	P	2312	C	1	0	2	1	T	tggaggccccagcgagat	0,837
G2			13	104833012	104841220	P	2476	C	0	0	2	0	Q	tggaggttccaccgagat	0,418
G2			15	19950298	19942117	S	2420	C	3	0	2	2	Q	tggaggtcccaccgagat	0,971
G2			10	86106187	86114386	P	2487	C	1	0	1	0	Q	tggaggtcccaccgagat	0,431
G2			13	68140880	68149001	P	2546	C	1	0	2	1			0
G2			14	112984891	112976666	S	2339	C	0	0	1	1	Q	tggaggtcccaccgagat	0,837
G2			3	16984867	16992988	P	2328	C	0	1	2	7	Q	tggaggtcccaccgagat	2,174
G2			6	132935927	132927704	S	2459	C	0	0	0	2	Q	tggaggttccaccgagat	1,674
G2			14	52521890	52529974	P	2403	C	2	0	0	0	T	tggaggccccagcgagat	0
G2			4	94746451	94754671	P	2317	C	0	1	2	1	Q	tggaggtcccaccgagat	0,837
G2			9	25612912	25604689	S	2455	C	0	0	1	0	Q	tggaggtcccaccgagat	0
G2			4	74538467	74546694	P	2372	C	1	0	3	2	Q	tggaggtcccaccgagat	0
G2			X	72524699	72532822	P	2024	C	1	0	2	0			0,725
G2			4	51892727	51900951	P	2510	C	1	0	0	0	Q	tggaggtcccaccgagat	0
G2			8	124718935	124727354	P	2282	C	0	0	0	2	Q	tggaggtcccaccgagat	0
G2			1	83868369	83860146	S	2516	C	0	0	0	0	q	tggaggttccactgagat	0
G2			10	17014683	17007273	S	2500	C	0	0	0	3	Q	tggaggttccaccgagat	0,418
G2			10	26624616	26616401	S	2529	C	1	0	0	0	Q	tggaggtcccaccgagat	0,418
G2			12	44831985	44823762	S	2401	C	0	0	2	2	T	tggaggccccagcgagat	0
G2			15	83021703	83029926	P	2458	C	1	0	1	0	Q	tggaggtcccaccgagat	0
G2			2	94602089	94610210	P	2541	C	0	0	1	2			0
G2			4	53968674	53960264	S	2427	C	0	0	1	0	Q	tggaggtcccaccgagat	0,418
G2			3	55674149	55665938	S	2471	C	1	0	1	1	Q	tggaggtcccaccgagat	0
G2			6	7669279	7677490	P	2514	C	2	0	0	1	Q	tggaggtcccaccgagat	0,392
G2			11	9350834	9342609	S	2470	C	0	0	0	0	Q	tggaggttccaccgagat	1,25
G2			1	52257434	52265662	P	2403	C	3	0	4	2	Q	tggaggtcccaccgagat	0
G2			X	41488765	41480534	S	2070	C	1	1	7	0	Q	tggaggtcccaccgagat	1,255
G2			1	58585489	58595457	P	2220	C	0	1	2	15	T	tggaggccccagcgagat	2,51
G2			13	118292973	118284583	S	2010	C	1	2	4	3	q	tggaggttccaccaagat	1,897
G2			12	41784059	41775843	S	2347	C	2	0	1	1	Q	tggaggtcccaccgagat	1,255
G2			Y	1872546	1864565	S	2035	C	2	0	2	2		tggaggccccagcaagat	0
G2			18	24683321	24675169	S	2500	C	0	1	1	0	Q	tggaggttccaccgagat	0,837
G2			10	25585205	25593428	P	2495	C	0	0	0	0	q	tggaggtcccagcgagat	0
G2			X	156453233	156445044	S	2050	C	4	0	11	1	Q	tggaggtcccaccgagat	1,478
G2			7	12068859	12077015	P	2095	C	6	0	5	0	q	tggaggtcccactgagat	1,258
G2			4	3291272	3282926	S	2240	C	5	0	12	2		tgggggtttccccactag	12,979
G2			5	82708425	82700203	S	2436	C	0	0	1	0	Q	tggaggttccaccgagat	0
G2			6	58755056	58746837	S	2495	C	0	0	1	1	Q	tggaggttccaccgagat	0
G2			17	34368311	34360087	S	2296	C	3	0	2	0	T	tggaggccccagcgagat	2,092
G2			8	26596457	26588328	S	2023	C	4	1	4	5			5,926
G2			2	20307110	20298885	S	2516	C	0	0	0	1	Q	tggaggtcccaccgagat	0
G2			Y	2041229	2049312	P	2026	C	4	1	8	3	q	tggaggtcccaccaagat	7,113
G2			4	39571122	39579646	P	2433	C	0	1	3	1	Q	tggaggtcccaccgagat	1,852
G2			12	20481297	20472878	S	2293	C	1	0	0	1	P	tgggggctcgtccgggat	0
G2			X	110081585	110090064	P	2496	C	5	0	2	2		tgggggctcgtcctggat	1,347
G2			12	21890390	21898565	P	2002	C	10	1	10	5	p	tggggcctcgtccgggat	3,834
G2			18	18992414	19000833	P	2020	C	5	0	9	12	p	tggaggctcatccgggat	3,043
G2			3	94762160	94770494	P	2196	C	4	1	6	6	G	tggtgcattggccaggaa	2,41
G2			4	11111258	11103136	S	2316	C	7	2	6	7		tgggggctcgttcgggat	0,758
G2			4	145841008	145846614	P	2116	C	0	0	2	-1	P	tgggggctcgtccgggat	
G2			4	146511565	146505959	S	2092	C	0	0	3	-1	P	tgggggctcgtccgggat	
G2			4	145569452	145576406	P	2153	C	0	0	4	-1	P	tgggggctcgtccgggat	
G2			14	41247460	41238878	S	2194	C	1	1	2	1		tgggggctcgtcccggga	0,805
G2			17	94976514	94968006	S	2323	C	4	0	1	0		tgggggctcgccccagga	0,201
G2			1	102614371	102606111	S	2178	C	1	0	8	0		tgggggctcgtcccggga	1,954
G2			14	40907571	40899181	S	2192	C	1	2	1	1		tgggggctcgtcccggga	0
G2			1	104629573	104637967	P	2102	C	7	0	17	8	p	agggggctcatccgggat	6,034
G3	PMV6	PMV	X	51501683	51510157	P	3088	C	0	0	0	0	Q	tggaggtcccaccgagat	0
G3	PMV19 10? 1	PMV	4	107652725	107643982	S	2628	C	0	0	0	0	Q	tggaggttccaccgagat	0,218
G3	PMV8	PMV	7	29324348	29332665	P	2648	C	0	0	0	0	Q	tggaggttccaccgagat	0
G3	PMV22	PMV	11	8820301	8829280	P	2837	C	0	0	0	0	Q	tggaggttccaccgagat	0
G3	PMV5 1? 11	PMV	5	44422900	44431622	P	2950	C	1	0	0	0	Q	tggaggtcccaccgagat	4,11
G3	PMV11	PMV	5	78005469	78014272	P	2945	C	0	0	0	0	Q	tggaggtcccaccgagat	0
G3	PMV1	PMV	X	14631230	14639971	P	2977	C	0	0	1	0	Q	tggaggtcccaccgagat	0
G3	PMV16	PMV	16	93594754	93586013	S	2927	C	1	0	0	0	Q	tggaggtcccaccgagat	0
G3		PMV	4	33126333	33118069	S	2394	C	1	0	3	-1	Q	tggaggttccaccgagat	
G3	PMV21	PMV	1	193634451	193643192	P	2565	C	0	0	2	1	Q	tggaggtcccaccgagat	0
G3	PMV14 8?	PMV	16	76026481	76017739	S	2929	C	0	0	1	1	Q	tggaggttccaccgagat	0
G3	PMV13	PMV	10	41125197	41134072	P	2937	C	0	0	0	0	Q	tggaggtcccaccgagat	2,196
G3	PMV23	PMV	4	101369892	101361089	S	2594	C	0	0	1	0	Q	tggaggtcccaccgagat	0,193
G3	PMV7 2?	PMV	2	57038510	57029318	S	2828	C	0	0	0	0	Q	tggaggttccaccgagat	
G3	PMV24	PMV	1	184094018	184102759	P	2564	C	0	0	1	0	Q	tggaggtcccaccgagat	0
G3	PMV12	PMV	5	144923629	144932809	P	2818	C	1	0	0	0	Q	tggaggttccaccgagat	
G3	PMV2	PMV	11	6658083	6649425	S	2387	C	0	0	2	0	Q	tggaggttccaccgagat	0
G3	PMV15	PMV	7	30397623	30406364	P	2584	C	0	0	1	0	Q	tggaggttccaccgagat	0
G3		PMV	12	55693276	55702271	P	2054	C	5	0	6	-1			
G3		PMV	10	8241473	8234255	S	2096	C	5	0	4	-1	Q	tggaggtcccaccgagat	
G3	PMV20	PMV	18	82828273	82836858	P	2874	C	1	0	0	0	Q	tggaggtcccaccgagat	0
G3	PMV9	PMV	13	100095189	100086448	S	2936	C	0	0	1	0	Q	tggaggtcccaccgagat	0
G3		PMV	19	38440157	38447227	P	2106	C	2	0	2	-1	Q	tggaggtcccaccgagat	0,429
G3	MPMV14	MPMV	11	76365341	76374029	P	2940	C	0	0	0	0	Q	tggaggttccaccgagat	0
G3	MPMV9	MPMV	3	152260864	152269551	P	2906	C	0	0	0	0	Q	tggaggttccaccgagat	0
G3		MPMV	13	21819875	21827661	P	2591	C	2	0	0	-1	Q	tggaggttccaccgagat	0
G3	MPMV3	MPMV	2	15946952	15938265	S	2937	C	1	0	0	0	Q	tggaggtcccaccgagat	0
G3	MPMV13	MPMV	5	24741102	24749719	P	2861	C	0	0	1	0	Q	tggaggtcccaccgagat	0

G3		MPMV	8	126052021	126060437	P	2473	C	0	0	2	12			
G3		MPMV	11	60402203	60394839	S	2265	C	1	0	2	-1	Q	tggaggtcccaccgagat	0
G3		MPMV	10	22424071	22432738	P	2929	C	0	0	0	0	Q	tggaggtcccaccgagat	0
G3	MPMV7	MPMV	5	43496527	43505213	P	2960	C	0	0	0	0	Q	tggaggtcccaccgagat	0
G3		MPMV	6	73223771	73213956	S	2547	C	0	0	2	-1			43,519
G3	MPMV6	MPMV	1	133470452	133479146	P	2862	C	1	0	0	0	Q	tggaggttccaccgagat	0
G3		MPMV	5	110148316	110140851	S	2459	C	1	0	2	-1	Q	tggaggtcccaccgagat	0
G3	MPMV8	MPMV	11	102900091	102908778	P	2865	C	1	0	1	0	Q	tggaggttccaccgagat	0
G3	MPMV4	MPMV	11	86698849	86707535	P	2944	C	0	0	0	0	Q	tggaggtcccaccgagat	0
G3	MPMV1	MPMV	7	64005512	64014552	P	2947	C	0	0	0	0	T	tggaggccccagcgagat	0
G3	MPMV11	MPMV	12	70465730	70474228	P	3000	C	1	0	0	0			0
G3		MPMV	5	122453776	122460838	P	2230	C	2	0	3	-1	Q	tggaggttccaccgagat	1,357
G3	MPMV5	MPMV	10	4628507	4620011	S	2728	C	1	0	3	2	Q	tggaggtcccaccgagat	0
G3	XMV18	XMV	19	60988354	60980003	S	2906	C	0	0	0	0	Q	tggaggttccaccgagat	0
G3	XMV15	XMV	9	62237522	62245905	P	2929	C	0	0	0	0	Q	tggaggtcccaccgagat	0
G3	XMV12?	XMV	8	44818939	44810580	S	2936	C	0	0	0	0	Q	tggaggttccaccgagat	0
G3	XMV19	XMV	14	53484913	53492360	P	2707	C	3	0	0	0	Q	tggaggttccaccgagat	0
G3	XMV9	XMV	4	146062965	146071316	P	3063	C	0	0	0	0	Q	tggaggtcccaccgagat	0
G3	XMV13	XMV	13	68392368	68384024	S	2914	C	0	0	0	0	Q	tggaggtcccaccgagat	0
G3	XMV16	XMV	9	41663510	41656480	S	2282	C	10	0	0	0			
G3	XMV8	XMV	4	145371516	145363002	S	2924	C	0	0	0	0	Q	tggaggttccaccgagat	0
G3	XMV41	XMV	1	173317855	173326267	P	2985	C	0	0	2	0	P	tgggggctcgtccgggat	0
G3	XMV17 ?	XMV	5	23221035	23212626	S	3147	C	0	0	0	0	P	tgggggctcgtccgggat	0
G3	XMV42 43?	XMV	1	172778230	172786635	P	3099	C	0	0	0	0	P	tgggggctcgtccgggat	0
G3	<eco>	EMV	8	126320498	126311126	S	2984	C	0	0	0	0	P	tgggggctcgtccgggat	
