# Supplementary material for: Murine Gammaretrovirus Group G3 Was Not Found in Swedish Patients with Myalgic Encephalomyelitis/Chronic Fatigue Syndrome and Fibromyalgia
Source: PLoS One. 2011 Oct 12;6(10):e24602. doi: 10.1371/journal.pone.0024602 (PMC3192035; doi:10.1371/journal.pone.0024602)
Supplement: Information S1 — Phylogenetic trees supporting the G1–G3 groups, and the G3 subgroups (poly-, modified poly- and xenotropic), as well as ecotropic, MERVs. Relationship to other MLV-related gammaretroviruses. (PDF) [file pone.0024602.s002.pdf]

Supplemental information S1

Analysis of variation and phylogeny of *gag*, *pol*, integrase and *env* genes, as well as of Gag, Pol and Env proteins.

Sequences from the 306 (later pruned to 300) gammaretroviral proviruses from assembly mm8 which scored 2000 and higher with RetroTector were aligned and subjected to phylogenetic analysis using the ClustalW and MUSCLE alignment programs, and the Neighbor Joining (NJ), Minimum Evolution (ME) and Maximum Likelihood (ML) algorithms for phylogenetic reconstruction. Bootstrap analysis was conducted with 500 bootstraps. MLV-like proviruses from non-mouse vertebrates are included as references. Trees **(Figure S1 A-E**) were rooted either on a sequence from rabbit (oryCun), or the ERVfc like sequences of dogs (Blikstad et al (2008), Cell Mol Life Sci. 65:3348-3365). Proviruses with ORF in *gag, pro, pol* and *env* are marked green. Reference sequences are marked magenta.

The *gag* based phylogenetic tree which is shown as Figure 1 in the main paper is here inspectable at high resolution (Figure S7a).

NJ Pol amino acid based Clustal guide tree

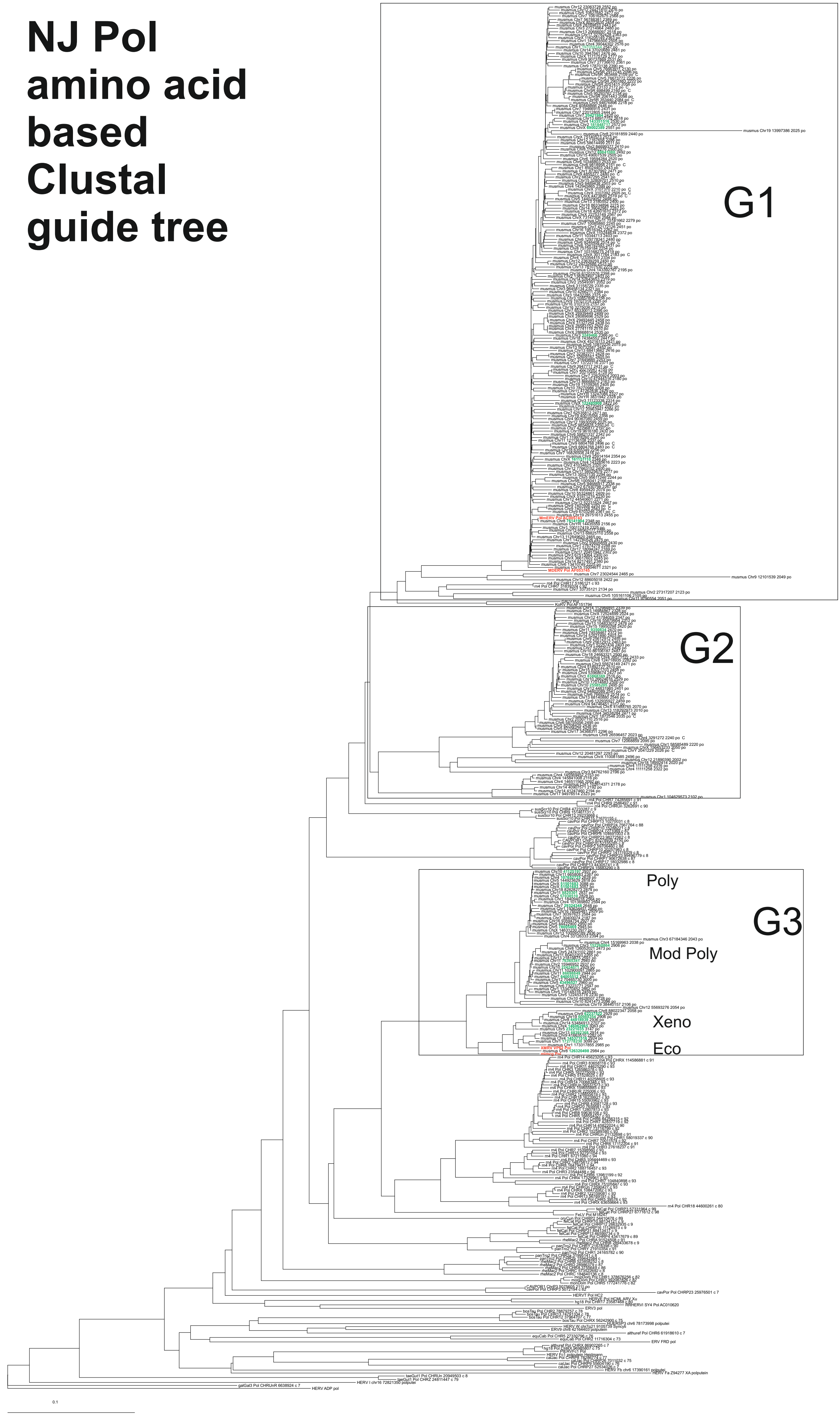

Fig. S1A

NJ Pol amino acid based tree, Clustal alignment with bootstrap values

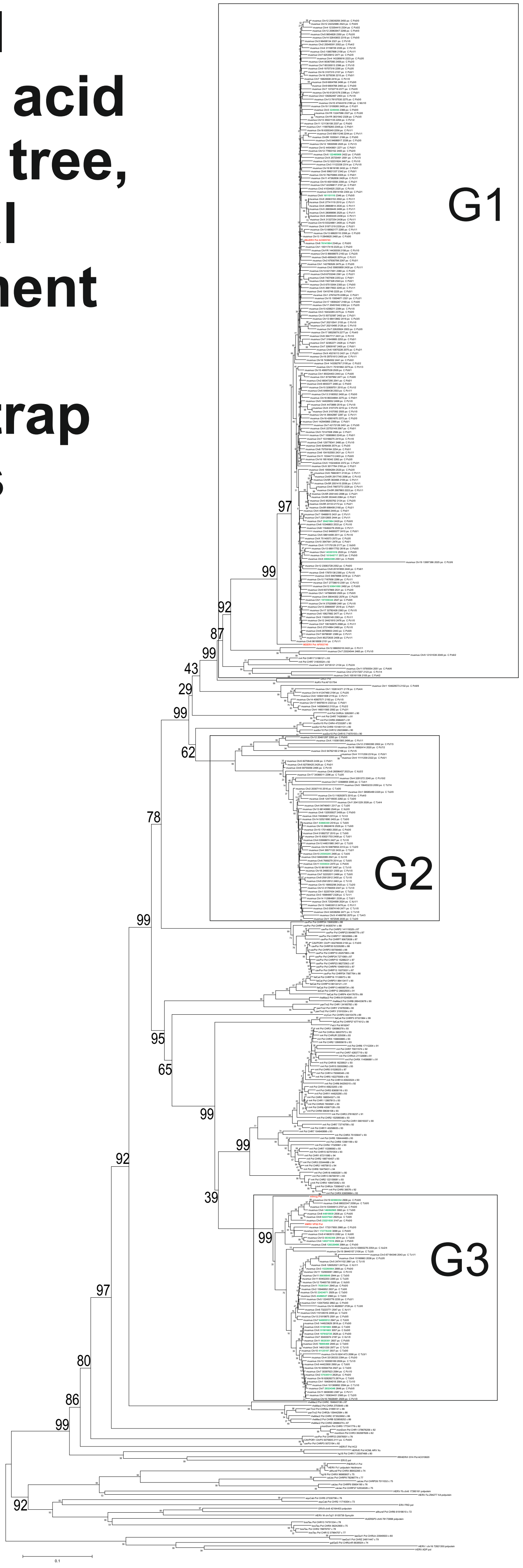

Fig. S1B

ME Pol amino acid based tree Muscle alignment, with bootstrap values

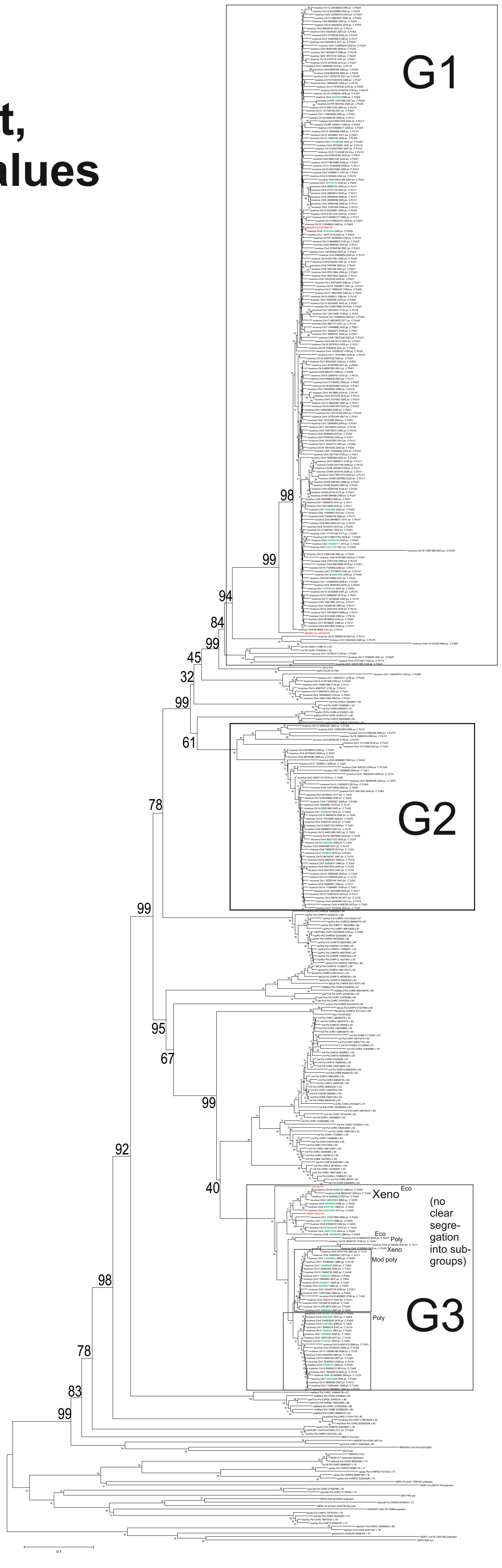

Fig. S1C

Clustal guide NJ tree of Env pro- teins, with a few refe- rence Envs

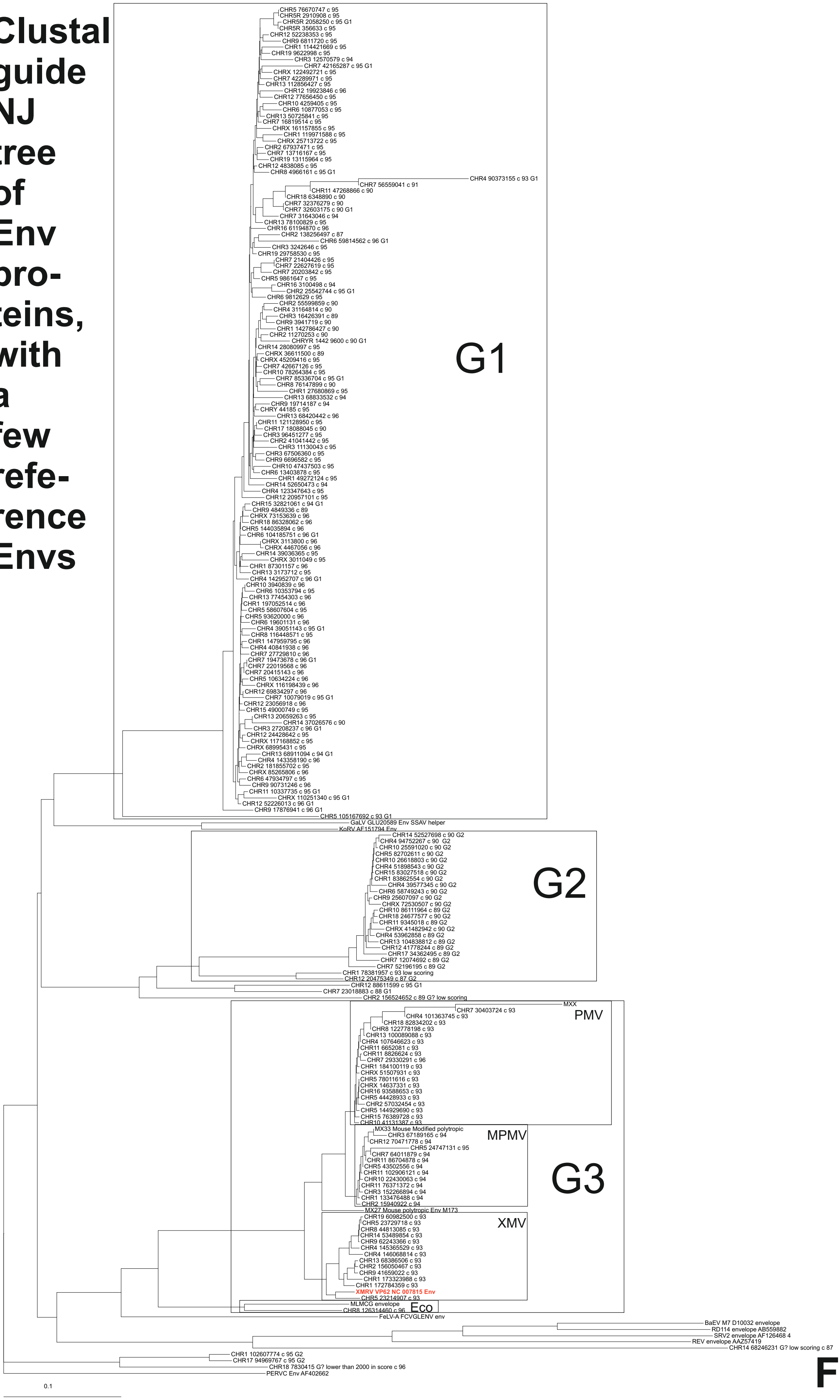

Fig. S1D

NJ *gag* nt based Clustal guide tree

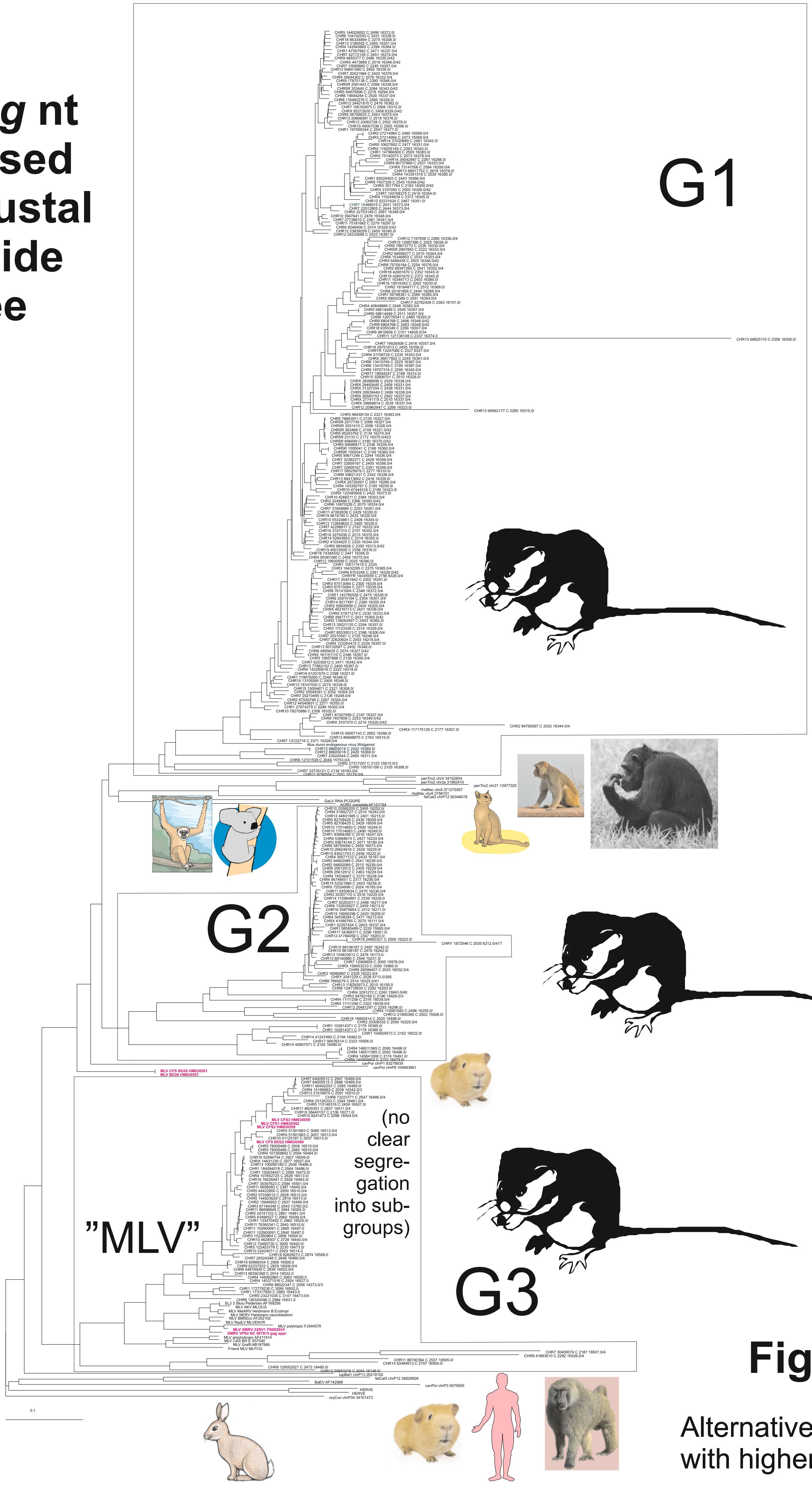

Fig. S1E

Alternative to figure 1, with higher resolution

Prediction of detection range for five XMRV/HMRV specific PCRs.

Figure S1 Fis a higher resolution version of Figure3 in the main paper. It allows detailed inspection of all branch labels, and the histogram of predicted amplification. Some branches were predicted to not be amplifiable because the sequences contained gaps or truncations affecting the target area. In the case of the gag amplimers from the Lo/Alter study (see reference in the main paper) the primer sequences were removed from the amplimers. They are shown in the trees (magenta) but scored 0 because of lack of primer target sequences.

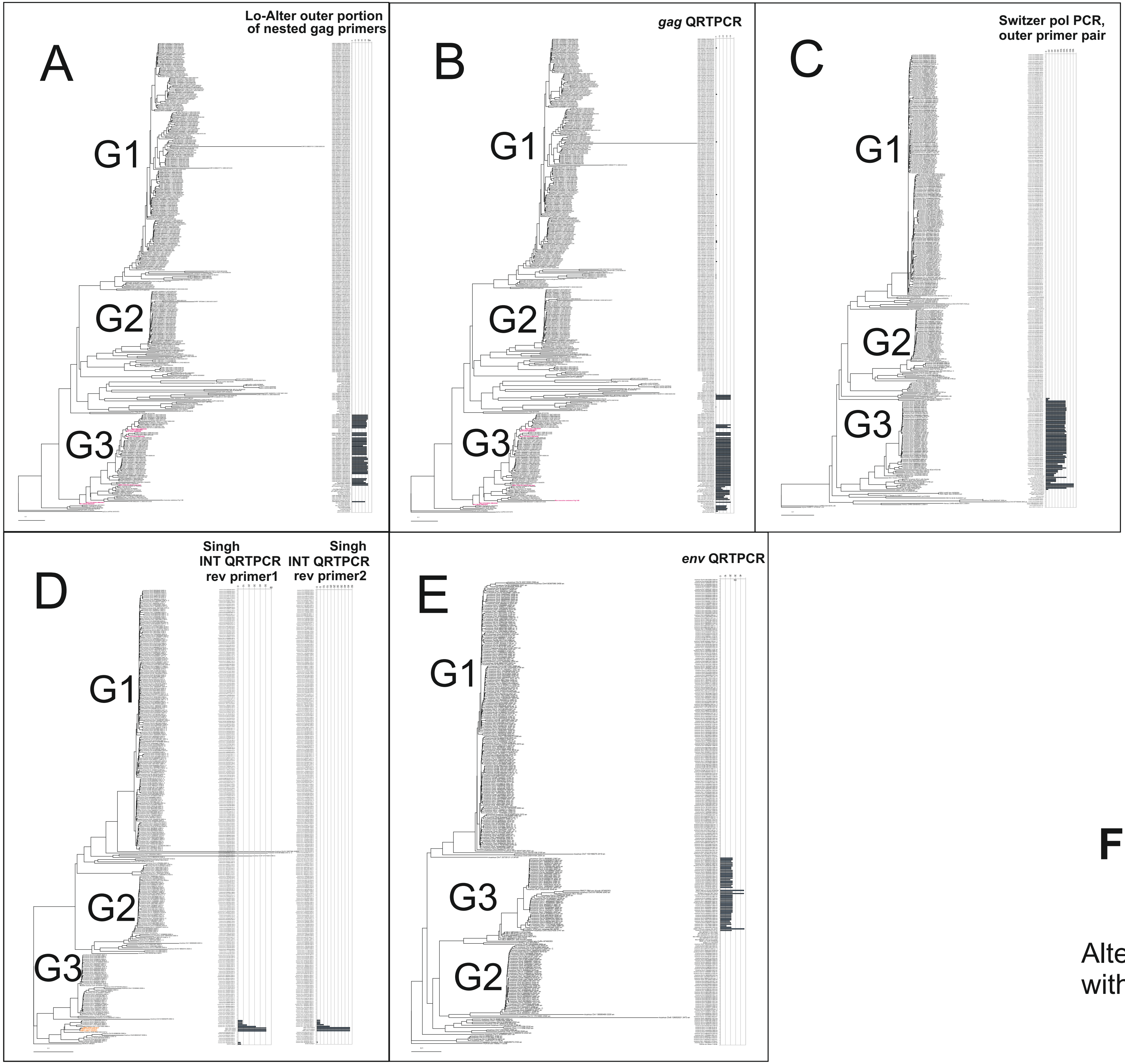

Fig. S1 F

Alternative to figure 3, with higher resolution
