# Supplementary material for: Murine Gammaretrovirus Group G3 Was Not Found in Swedish Patients with Myalgic Encephalomyelitis/Chronic Fatigue Syndrome and Fibromyalgia
Source: PLoS One. 2011 Oct 12;6(10):e24602. doi: 10.1371/journal.pone.0024602 (PMC3192035; doi:10.1371/journal.pone.0024602)
Supplement: Information S2 — Xenotropic cell culture contaminating retroviruses and the uniqueness of 22RV1/XMRV. (DOC) [file pone.0024602.s003.doc]

**S10. A closer look at the origins of 22Rv1 and other retroviral cell culture contaminants**

Several infectious murine gammaretroviruses have been discovered. Some are adventitious contaminants of cell cultures. We compared the genetic background of some infectious murine gammaretroviruses, of which some were xenotransplantation-associated xenotropic cell culture contaminating retroviruses, with that of 22RV1/XMRV. .


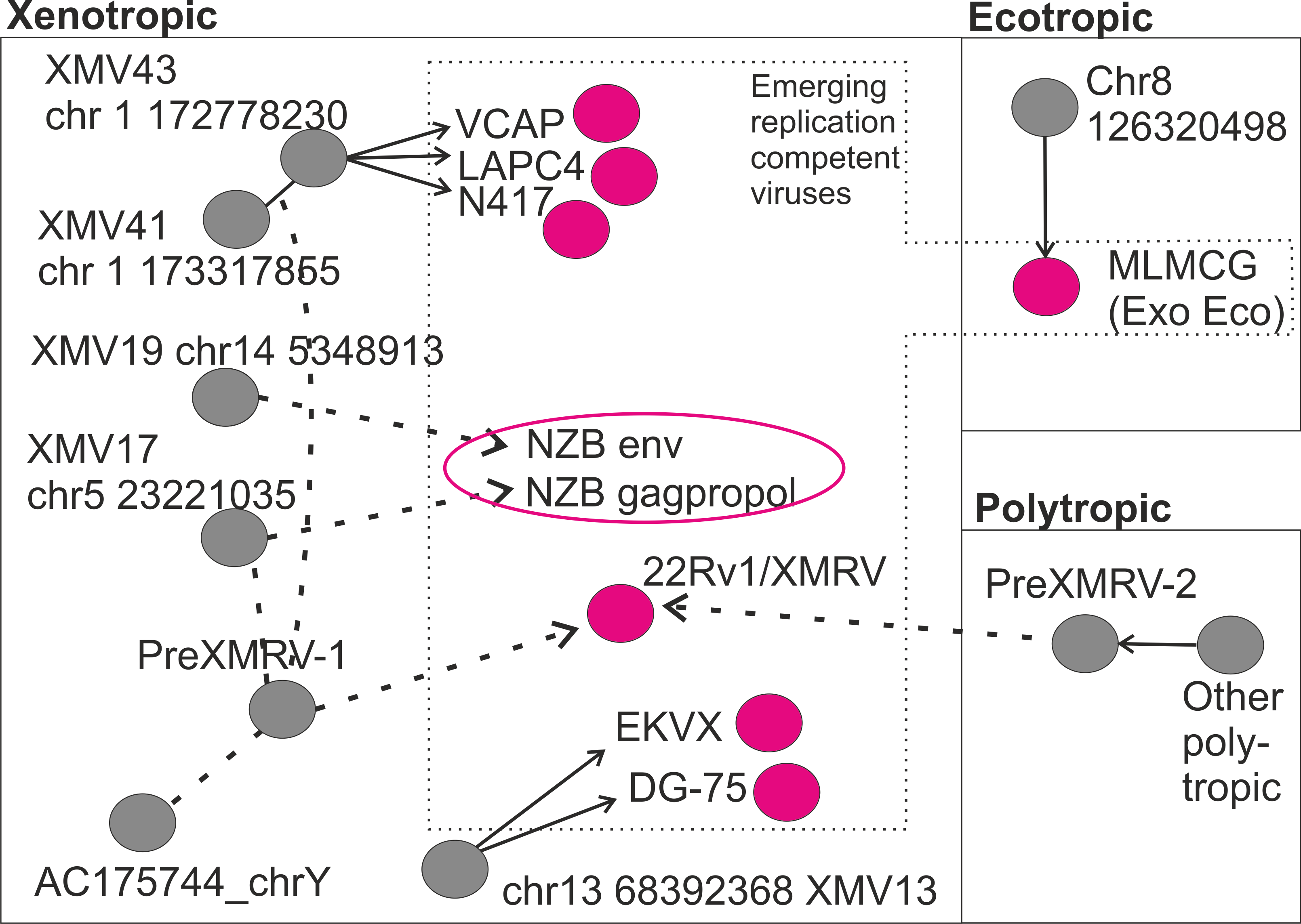


Figure S10_1. Approximate relationships between some endogenous and exogenous murine gammaretroviruses. Relationships due to gradual evolution from an ancestor without clear evidence of recombination are shown by a solid line. Relationships which involve partial contributions to a recombinant virus are shown by a stippled line. Line lengths do not correlate with degree of similarity, but the figure is based on a ClustalW alignment of full length proviral nucleotide sequence, and Simplot studies of their interrelation. Gray dots symbolize murine endogenous retroviruses, with their chromosomal position, and Jern subgroup (Jern et al, Plos Genetics 2007), if known. The magenta dots symbolize exogenous retroviruses which were discovered as cell culture contaminants (Raisch et al, Virology 2003, Zhang et al Cancer Biology and Therapy 2011, and Sfanos et al Plos One 2011, see reference list for the main paper).The figure was based on an alignment of the mm8 proviruses from the prototype of RetroBank and gammaretroviral proviruses from GenBank : VCAP; JF908815.1, LAPC4; JF908816, N417; HQ246218.1, NZB-9-1 gagpropol; EU035300.1, NZB env; EU334447.1, PreXMRV-1; NC_007815, EKVX; JF908817.1, DG75; AF221065.1, PreXMRV-2; FR871850, XMRV_VP62; EF185282.1, 22Rv1; FN692043.2

.

The exogenous ecotropic MLVs (like Moloney MLV; MLMCG) are similar to an endogenous G3 provirus at mm8 chr8 126320489, (from the prototype of RetroBank, “RetroBank”).

The recombination between PreXMRV-1 and PreXMTV-2, which is the likely source of 22RV1 and XMRV, is entirely different from the likely recombination(s) which gave rise to NZB. NZB is a prototypic xenotropic virus which also seems to be a recombinant (data not shown). The other replication competent xenotropic retroviruses have evolved without major recombination events from endogenous precursors, judging from an even distribution of dissimilarity in plots between them and their probable ancestors. These xenotropic viral sequences are also distinct from 22Rv1/XMRV.

The figure was based on similarity plots between endogenous and exogenous proviruses selected for similarity to PreXMRV-1 and PreXMRV-2. No exact endogenous counterparts of these sequences were found in RetroBank, or in GenBank. However, 14 highly related sequences were found, and subjected to similarity analysis (Simplot). Some of the Genbank hits were from genomic clones of 100 000 – 200 000 nt. The proviral information was extracted from them by using RetroTector online (<http://www.neuro.uu.se/fysiologi/jbgs/>). PreXMRV-1 was almost identical (>95%) to a provirus in AC175744.2 from mouse chromosome Y (GenBank) between 0-810, 1050-4200, 5790 – 6530 and 6990-7630, The same pattern was observed with mm8 chr5 23221035 XMV17 (RetroBank). mm8 chr1 173317855 XMV43 and mm8 chr1 172778230 XMV41 (RetroBank) were almost identical (>95%) to PreXMRV1 between 1 and 1800, and between 3400 and 4400. This indicates that PreXMRV-1 had a complex recombinatorial origin from several xenotropic G3 proviruses. PreXMRV-2 was almost identical (>95%) to mERV_XL_JF71465 (Mendoza et al, J Virol 2011) over the entire provirus. It was also almost identical (>95%) to the consensus of group G3 (this paper), except for a sharp peak of dissimilarity between 5000 and 5500. mm8 chr4 107652725 (PMV 10 or PMV19; the Jern subgroup assignments are sometimes ambiguous), mm8 chrX 14631230 PMV1 (RetroBank), and a provirus in CU407131.8 from mouse chr11 (GenBank), had a high similarity (>95% identity) to the G3 consensus and most of PreXMRV-2 throughout the provirus. PreXMRV-2 seems to be a “mainstream” polytropic group G3 provirus. The majority of the group G3 proviruses are polytropic **(**Jern et al, Plos Genetics 2007).
