# Supplementary material for: Murine Gammaretrovirus Group G3 Was Not Found in Swedish Patients with Myalgic Encephalomyelitis/Chronic Fatigue Syndrome and Fibromyalgia
Source: PLoS One. 2011 Oct 12;6(10):e24602. doi: 10.1371/journal.pone.0024602 (PMC3192035; doi:10.1371/journal.pone.0024602)
Supplement: Information S3 — The G1–G3 groups. Properties and consensus sequences. (DOC) [file pone.0024602.s004.doc]

**S9. The three groups of murine endogenous gammaretroviral sequences**

1. **General.** The groups are based on the 306 proviruses detected by RetroTector in assembly mm8 from C57Black/6J. The 306 were reduced to 300 after removal of duplicate proviruses reported by RetroTector. The groups were defined with the help of a tree-branch defining grouping program written by JB . The program also calculates a group majority consensus from a ClustalW183 alignment, and basic group characteristics. Only mm8 sequences are included in these calculations.

Abbreviations:

Gln1 PBS sequences: TGGAGGTTCCACCGAGA,

Gln2 PBS sequences: TGGAGGTCCCACCGAGAT .

The PBS sequence, and its interpretation, of each group member are given in the S8 group table.

The groups are further defined in the table S8, and in the phylogenetic trees, Figure 1 in the main text, and supplementary figures S6A-D and S7a. The groups were defined from the pol nucleotide alignments and trees. However, the three groups were consistent, regardless if nucleotide or amino acid sequences from *gag*, *pol* or *env* were used. However, a few discrepancies occurred in the Env amino acid trees compared to the trees based on *gag* and *pol* (see below). The subgroups within G3 (PMV, MPMV, XMV or Ecotropic) only segregate clearly, and as expected, in trees S6A (Neighbor joining with pol nucleotides) and S6D (envelope amino acids).

In general, exogenous ecotropic sequences clustered with mm8 chr8 12634098 within group G3. Amphotropic sequences behaved ambiguously. Sometimes they clustered within group G3, sometimes outside of it, coming out ancestral to groups G2 and G3. *Mus musculus castaneus* virus FRG1 (AB050720.1, Ikeda et al, J Virol 2001) clustered with some of the XMVs. Only a subset of the trees are shown here.

Tree S6D (Env proteins) has several interesting features. All mm8 gammaretroviral Envs with ORF or near-ORF (maximum of one stop or shift) were included in this alignment and tree. The envelope proteins of two G1 sequences and two G2 sequences clustered differently from the *gag* and *pol* based trees, thus giving evidence for intergroup recombination. The G1 sequences chr12 88611599 and chr7 23018883 clustered away from the rest of the G1 proviruses, together with a Env of a low scoring provirus, chr2 156524652. The G2 sequences Chr1 102607774 and Chr17 94969767 clustered together with Env of a low scoring provirus (chr18 7830415) away from the rest of the G2 sequences, together with a PERV C Env (AF402662).

1. **The groups**

**Group 1, mmERV and MDERV like:** 188 members.

The number of members were identical regardless if the group was defined from a *gag* nt or a *pol* nt tree. As defined in tree S6A it includes two rat sequences: rn4 chr17 5186121 and rn4 chr7 31839324 from the prototype of RetroBank. In the Env tree S6D chr7 31839324 was also found in G1, but not chr17 5186121. Two additional rat sequences were included in G1 in Env tree S6D: chr7 83495068 and chr3 161735203. Together with the discrepancies between *gag* or *pol* based trees on one hand versus env based trees mentioned above on the other These aberrations illustrate that envelope genes sometimes are subject to recombination. The details behind these recombinations are beyond the scope of this paper. The previously described mmERV (Bromham et al, J Virol 2001; here shown to be identical to mm8 chr8 76141904; it has ORF in *gag*, *pro*, *pol* and *env*) and MDERV AF053745 from GenBank were also included. The highly related GaLV and KoRV were not included.

Further information on mmERV can be found in <http://acb.qfab.org/acb/retrovirus/>

Average within-group % identity (Clustalw1.83) : 94.31+/- 0.53 (SEM)

Average % identity to consensus sequence (Clustalw1.83) : 94.95+/- 0.44 (SEM)

PBS usage : Q 0, (Gln1 0, Gln2 0), P 91, S? 6, T 0, G 79, and 7 with unknown PBS assignment. Sum 188 (S? means a questionable identification of a Serine tRNA PBS).

The here described frequent use of a glycine tRNA as PBS in this group seems to be a novelty.

**Group 2, GLN MERV like:** 59 members.

This group consists entirely of mm8 sequences. It contains the previously recognized sequences GLN1, GLN2 and GLN3 (Ribet et al J Virol 2007, see the reference list in the main paper). They were here defined, using RetroTector online, from the cosmid sequences AC136922, AC153548 and AL669853 (Genbank), respectively . As mentioned above, a few G2 envelopes branched with a PERVC envelope, away from the rest of the G2 envelopes.

GLN1 is identical to mm8 chr1 83868369 and has *gag*, *pro*, *pol* and *env* ORFs.

GLN2 is identical to mm8 chr10 25585205 and has *gag*, *pro*, *pol* and *env* ORFs.

GLN3 is identical to mm8 chr11_9350834 and has *gag*, *pro*, *pol* and *env* ORFs.

These three reference proviruses are the three only bioinformatically completely intact proviruses of group G2 in mm8. Group 2 can therefore also be referred to as “GLN MERV”.

Average within-group % identity (Clustalw1.83) : 85.28+/- 2.03 (SEM)

Average % identity to consensus sequence (Clustalw1.83) : 90.42+/- 1.92 (SEM)

PBS usage : Q 34 (Gln1 10, Gln2 24), P 7, T 5, G 1, 4 with no PBS and 9 with unknown PBS sequence. Sum 59.

**Group 3, MLVs:** 53 members

This group closely corresponds to the endogenous representatives of the murine leukemia viruses (MLVs). As shown in Figure 1 of the main paper, and supplementary figures S6A-F, it can be considered to include the highly related exogenous ampho- and ecotropic MLVs, although the status of the amphotropic sequences is somewhat uncertain.

Average within-group % identity (Clustalw1.83) : 92.55+/- 0.83 (SEM)

Average % identity to consensus sequence (Clustalw1.83) : 93.30+/- 0.84 (SEM)

PBS usage : Q 44 (Gln1-TGGAGGTTCCACCGAGAT**-**18, Gln2 –TGGAGGTCCCACCGAGAT- 23, Q? 1), P 4, T? 1, 6 had no detected PBS sequence. Sum 53

The assignments with the Jern nonecotropic subgroups can be seen in table S8. PMV1, PMV10, PMV17, PMV4, PMV8, XMV10 and XMV42 were not found. Judging from the branch patterns of *gag* nucleotide, *pol* nucleotide and Env protein trees (see supplementary material) group G3 contains 23 polytropic, 18 modified polytropic and 11 xenotropic proviruses, and one ecotropic provirus (chr8 126320498). The concordance between our G3 members and the Jern subgroups is high, but not complete. This remains to be sorted out. The subgroups were only evident in some trees. The clustering most consistent with the Jern subgroups was most consistent in the Env based tree S6D. The eco-, xeno-, poly- and modified polytropic properties are all envelope dependent, so this behavior was expected.

1. **Consensus sequences**

In the following ,the consensus nucleotide and protein sequences of the three groups are given. A few frame shifts occurred in the original consensus sequences. These were manually corrected using the sequence of the most similar mm8 member as a template. In large phylogenetic analyses, the consensus sequences can be used as representatives of the groups. They may also reveal some features of the ancestors of the three groups.

**Corrected G1 consensus :**

>ConsG1

TGAGATAAGGGCCTCCTGGAACAACCTCAGAATGAACCGGGTACATTGCCAAATAATAGGACATGACCCCTTAGTTACGTAGAATCCCTTGGCAGAACCCCTTGTCCCTTGGCAGAACCCCTTAGTTATGTAAACTTGTACTTTCCCTGCCCCGCTCTCCCCCCTTGAGTTTTCCTATATAAGCCTGTGAAAAATTTTGGCTGGTCGTCGATTCTCCTCTACACCACTAGGTGCATGAGTTTCGACCCCAGAGCTCTGGTCTATGTGCTTTCTTGCTGTTGCTTTATTAAATCTTGCCTTCTACATTTTGAGTTCGGTCTCAGTGTCTTCTTGGGTCCGCGGCTGTCCCGAGGCTTGAGTGAGGGTCTCCCTTCGGGGGTCTTTCATTTGGGGGCTCGTCCGGGATCAGCGCGACCACCCAGAGGTCCTAGACCCACTTAAAGGTAAGATTCTTTGTTCTGTCTTGGTCTGGTGTCTGTGTTCTGTTTCTAAGTTTGGTGCGATCGCAGTTTCGGTTTTGCGGACGCTCAGTGAGACCGCGCTCCGAGAGGGAACGCGGGGTGGATAAGGATAGACGTGTCCAGGTGTCCACCGTCCGTTCGCCCTGGGAGACGTCCCAGGAGGAACAGGGGAGGACCAGGGACGCCTGGTGGACCCCTTTGGAGGCCAAGAGACCATCTGGGGTTGCGAGATCGTGGGTTCGAGAGTCCCACCTCGTGCCGTTGCGAGATCGTGGGTTCGAGTCCCACCTCGCGTTTTGTTGCGAGATCGTGGGTTCGAGTCCCACCTCTTTTCCTCGCGCCTTGTTGCGAGACCGTGGGTTCGAGTCCCACCTCGGTTTGTGTTACGGGATCGTGGGTTCGAGTCCCACCTCGTGCAGAGGGTCTCAATCGGCCGGCCTTAGAAAGGCCATCTGATTCTTTGAGTTGCTTGTGGTCGACGCGAAGTCGCCGCCGTTTTTGGTTTCTTTTTTGTCTTAGTCTCGTGTTCGCTCTTGTTGTGTCTACTATTATTCTAGAAATGGGACAATCTGTGTCCACTCCCCTTTCTCTAACTCTGGAGCATTGGAAGGAGGTGCAGGTCAGAGCCCACAACCAGTCGGTGGAGGTCAGAAAGGGTCCGTGGCAGACCTTTTGCGCCTCCGAGTGGCCGACGTTTGGAGTGGGCTGGCCACCAGAAGGTGCTTTTGACTTGTCACTAATCGCCGCCGTCAGGCGAATTGTTTTTCAGGAGGAAGGGGGTCACCCTGATCAGATCCCCTACATTGTGACCTGGCAGAATCTCGTCCAATTCCCACCTCCGTGGGTCAAGCCTTGGACCCCAAATTCTTCGAAACTGACGGTCGCGGTTGCCCAGTCTGATGCAGCCGGAAAGTCCGGCCCGTCAGCACCCCCCAAGATCTATCCAGAGATTGACGACCTCCTCTGGATGGACTCCCAACCTCCCCCTTACCCCCTGCCCCAGCAGCCACCTGCAGCCGCCCCACCACAGGGACCAATAGCGAGAGGGGCTCAGGGACCGGCGGGGGGGACTCGGAGCCGCCGAGGCCGAAGCCCCGGGGAGGAAGGGGGGCCAGATTCAACAGTTGCCTTGCCACTTAGAGCACATGTGGGAGGGCCAGCGCCAGGACCTAATGATCTCATTCCTTTACAGTACTGGCCTTTTTCCTCTTCTGATTTATATAATTGGAAAACTAACCACCCTCCCTTCTCAGAGAACCCCTCTGGGCTTACTGGGCTCCTTGAGTCACTTATGTTCTCCCATCAACCCACTTGGGATGATTGTCAGCAGCTTTTGCAGGTTCTTTTTACCACAGAAGAAAGAGAAAGAATCCTGATGGAGGCGAGAAAAAATGTTCTGGGAGAGGACGGCACACCCACTGCCCTCCCTAACCTCGTGGACGAGGCTTTCCCCTTGAACCGCCCCAACTGGGACTACAACACCGCGGAAGGTAGGGGACGCCTCCTTGTCTATCGCCGGACTCTAGTGGCAGGTCTCAGAGGAGCCGCTAGACGGCCCACCAATTTGGCTAAGGTAAGAGAGGTCTTGCAGGGGCAGACTGAACCACCCTCAGTCTTCCTTGAGCGTCTAATGGAGGCATATAGGAGGTACACCCCTTTTGACCCCTTGTCAGAGGGGCAGAGAGCCGCTGTAGCCATGGCCTTCATTGGTCAGTCCGCTCCCGACATTAAGAAAAAGCTGCAAAGGCTGGAGGGGCTCCAAGATCATACGCTCCAAGATTTAGTAAAAGAAGCAGAGAAAGTCTATCATAAGAGGGAAACAGAAGAAGAGAGGCAGGAGAGAGAGAAGAAAGAAATGGAGGAGAGGGAAAATAGACGGGATCGCCGTCAGGAGAGAAATTTGAGTAAAATTTTGGCCGCAGTTGTAAATGATAGACAGTCAGGAAAAGGTAAAATAGGGCTCCTGGGCAACAGGGCAGTGAAACCGCCAGGTGGCAGAAAGATACCACTGGAAAAAGACCAATGCGCCTATTGCAAAGAGAAAGGACACTGGGCTAGAGATTGCCCTAAAAACCGGGAGCGATCCAAGGTCCTGACCCTAGAAGATGATTAGGGAAGTCGGGGCTCAGACCCCCTCCCTGAGCCTAGGGTAACTTTGTCCGTGGAGGGGACCCCCGTCAACTTCCTGATAGACACCGGAGCAGAGCATTCAGTACTCACTAGCCCCCTAGGCAAGCTAGGCTCTAAAAAGACCATGGTGATTGGAGCCACTGGTAGTAAATTTTACCCCTGGACGACCGAACGAGCCCTACAGATAAACAAGAACATAGTGACCCACTCCTTCCTGGTGATACCTGAGTGTCCTGCTCCCCTCTTGGGGCGCGATCTGCTAACCAAACTAAAGGCTCAAGTCCAATTTACTTCAGAAGGCCCACAAGTAAGCTGGGGAAAAGCCCCCGTTGCCTGCCTTGTCCTCAACACAGAGGAAGAGTACCGGTTGCATGAAGAGCAACCCAAAAATGCAGTCTCTTCAGGCTGGCTAACTGCGTTCCCCAATGTCTGGGCAGAACAAGCAGGAATGGGGTTGGCTAAACAAGTGCCTCCGGTTGTGGTAGAACTTAAAGCTGATGCCACCCCCATCTCGGTAAGACAATACCCCATGAGCAAGGAAGCTAGGGAGGGCATCCGGCCTCATATCCAGAGGTTGCTAGACCAAGGAGTTTTAGTGGCCTGTCAGTCCCCCTGGAATACACCACTTCTGCCGGTTCGAAAACCAGGGACCAATGACTATCGCCCGGTGCAAGACCTCCGGGAAGTTAACAAAAGGGTCCTGGACATTCACCCCACAGTCCCGAACCCGTACAATTTATTAAGCTCTCTCCCACCTGAGAGAACATGGTATACAGTCCTGGACTTAAAAGATGCCTTCTTTTGCCTGCGTTTGCACCCTAAGAGTCAGCTCCTGTTTGCCTTTGAATGGAGGGACCCAGAGGGCGGACAGACTGGTCCAACTAACTGGACTAGGCTACCACAGGGGTTCAAAAATTCCCCCACCCTGTTTGACGAGGCCCTCCATCGGGATCTCGCGCCTTTTCGCGCTCGAAACCCTCAGCTTACCCTACTACAGTATGTGGATGATCTCTTGGTCGCGGCGGCCTCGAAGGAGCTGTGTCACCAGGGAACTGAGAGGCTCCTCGCAGAACTGAGTGACTTGGGGTATCGAGTTTCGGCTAAAAAGGCACAAATCTGTCAAACTGAGGTAACCTACCTGGGGTATACCCTCCGAGGGGGTAAAAGATGGCTCACAGAAGCCCGGAAGAAGACTGTTATGATGATCCCATCGCCAACCACCCCACGGCAGGTACGTGAGTTTCTGGGGACTGCTGGCTTTTGTAGACTCTGGATTCCAGGCTTTGCAACCCTAGCGGCACCTCTATATCCTTTGACTAAGGAAGGGGTTCCTTTTGAGTGGAAAGAAGAGCACCAAAGAGCTTTTGAGGCTATCAAGTCGTCTCTAATGACTGCCCCCGCGCTAGCATTACCAGACTTGACTAAGCCTTTCGTCCTATATGTGGACGAGAGAGCGGGTGTAGCCAGGGGAGTGTTGACACAAGCACTGGGACCCTGGAAGAGACCTGTAGCCTATTTGTCAAAAAAATTAGATCCCGTTGCTAGTGGATGGCCCACATGTCTGAAAGCTATTGCAGCAGTAGCCCTGCTGATCAAAGATGCTGACAAATTGACAATGGGACAACAGGTGACTGTTGTAGCCCCTCATGCCTTGGAAAGTATCGTGCGGCAGCCACCTGACAGATGGATGACAAATGCCCGAATGACACACTATCAGAGCCTGCTGCTAAATGAGCGTGTAACCTTTGCGCCCCCTGCCATCCTCAACCCAGCTACCCTTCTCCCTCTAACAAATGATTCCGTCCCAGTACATCAATGTATGGACATCCTCGCTGAAGAAACTGGGACCAGAAGGGACCTGACTGACCAACCCTGGCCTGGAGCTCCCAGTTGGTATACGGACGGCAGCAGTTTCCTGATAGAGGGGAAGCGAAAGGCTGGAGCTGCGGTGGTGGACGGGAAAAAGGTAATTTGGGCAAGCGCTTTGCCTGAAGGAACATCGGCACAAAAGGCTGAACTTATAGCGCTTATACAAGCCCTCCGAGAGGCTAAAGGTAAGATCGTTAACATCTACACTGACAGCCGCTATGCTTTTGCTACCGCACACATCCATGGGGCCATCTACAGGCAGCGAGGGCTATTGACTTCGGCTGGTAAAGACATTAAAAACAAAGAAGAAATTCTGGCCCTGTTGGAAGCCATACATGCACCTAAGAAGGTAGCCATCATCCACTGCCCCGGCCACCAAAGAGGAGAAGACTTGGTGGCCAAGGGCAACCGAATGGCAGACTCAGTGGCAAAACAAGTTGCTCAAGGGGCCATGATCTTAACTGAAAAAGGTGATCCGCCCAAAAGCCCTGAGGATGAGAGGTATAACATAAAAGAGCTATTGTGGACCAGTGATCCCCTCCCATACTTTTTTGAAGGGAAAATAGAATTGACTCCCGAAGAAGGAATAAAATTTGTGAAAGGACTACACCAATTCACCCACCTGGGAGTTGAAAAAATGATGAGACTAATTAAAAATTCCCGATACCAAGTCCCCAACCTGAAGTCAGTGGCTCAAAAGATTATAGACTCCTGCAAACCATGTGCATTCACTAATGCGACTAGAGCCTACAAAGAACCTGGAAAGAGACAACGGGGAGACCGTCCTGGAGTGTATTGGGAGGTAGATTTTACTGAAGTTAAACCTGGAATGTATGGTAACAAGTATCTGTTAGTATTTGTAGACACCTTTTCAGGATGGGTTGAAGGCTTTCCCACTAAAACTGAGACTGCCCAGATTGTGGCCAAGAAGATCCTTGAAGAAATCCTGCCAAGATTTGGAATCCCTAAGGTAATCGGGTCCGACAATGGACCAGCCTTTGTTGCCCAGGTAAGTCAGGGCTTGGCCACTCAGTTGGGCATCGATTGGAAATTACACTGTGCTTACCGCCCTCAAAGCTCAGGACAGGTAGAGAGGATGAATAGGACCTTAAAAGAGACCTTGACTAAATTAGCCATTGAGACCGGCGGGAAAGACTGGGTGGCTCTCCTCCCTCTTGCGCTCTTCCGAGCCCGAAACACCCCTGGACGTTTCGGGCTCACTCCTTTTGAAGTTCTGTATGGAGGACCTCCCCCCTTAATGGAAGCTGGTGGAACATTGGTTTCCGACTCTGACCCTGTCTTACCCTCCTCTTTGCTTATTCATTTAAAGGCCCTAGAAGTGATTAGGACCCAGATTTGGGACCAACTGAAGGCAGCCTATACCCCAGGGACCACCGCAGTACCCCACGGGTTCCGAGTTGGAGACAAAGTCTTGGTCAGACGGCATCGAACCGGCAGCCTCGAGCCACGGTGGAAGGGACCCTATTTGGTGTTACTGACAACCCCTACTGCGGTAAAAGTTGACGGGATTGCCTCCTGGATCCACGCCTCCCACGTCAAGAGGGCCGCCAGTCAAGATGAAGAAAACCACGAAGACAATTGGACAGTGGCAGCCACTGACAATCCTCTTAAGCTTCGTTTGCGCCGCAGGCGCCACCCTGAGCCTAGGGAACCATAACCCTCATGCTCCAATTCAACAGTCCTGGGAAGTGCTTAATGAGGAGGGAAACATTGTATGGGCAACCACTGCAGTCCATCCCCTCTGGACTTGGTGGCCTGATCTCACACCTGACATCTGTAAGTTAGCGGCAGGATCCCCCAATTGGGACCTCCCTGATCATACTGATCTTAGCAACCCACCCCCTGAAGAGCGGTGTGTCCCAAACGGGATAGGGAGCACATATGGGTGTTCGGGGCAGTTCTACCGAGCTAATCTTAGAGCTGCACAATTTTATGTTTGCCCTGGTCAGGGTCAGAGCAAAAGGCTTCAACAAGAATGTGGGGGGGCATCAGATTACTTTTGTGGTAAATGGACATGTGAAACGACAGGGGAAGCTTACTGGAAGCCCTCCTCTGACTGGGACCTAATCACGGTAAAACGAGGTAGTGGCTATGATAAGTCAAACCAAGGAGAAAGAAACCCCTATAAATATCCCAGAAATGGGTGCGCTTTTAAAAACAGCCCCCCAGGACCATGCAAAGGTAAATACTGCAACCCCCTACTTATAAAGTTCACCGAGAAAGGGAAACAACACCGTCTAAGTTGGCTTAAAGGAAATAGGTGGGGTTGGCGAGTATACATTCCACTAAGAGATCCTGGGTTCATTTTCACGATCAGACTGACAGTGAGAGACCCGGCGGTGACACTCGTAGGGCCCAACAAGGTCCTTATAGAACAGGGCCCCCCAGTCGTACCGGCTCCCCCAAAGGTCCCGGCCGTACCAGCTCCACCAACTCCACAGCCCAACACAGTGGTACCCTCCCTAGGGACTAATACTCCCCTCATAAAGCCTACCTTGGCTTCCCCACCGCCCCTAGGTACAGAGAACCGTCTGGTCAGTCTAGTCCAGGGAGCTTTTTTAGCTTTAAATAGAACTAACCCTAATATGACTCAATCATGCTGGTTATGCTATGCCTCTAGCCCCCCTTATTATGAAGGAATAGCTCAGATCAGGACTTATAATATTACTTCAGATCATTCTCAATGCCTTTGGGGAGAAAACAGAAAGTTGACTCTAGCAGCAGTTTCAGGAAGACGGGCTTTGTTGGGCCAGGTACCTCAGGATAAAGGGCACCTCTGTAATCAGACCCAGAACATCCAGTCTAGCAAAAGTGGTCAGTATCTAGTGCCCCCCCTAGACACAGTGTGGGCTTGCAATACCGGTCTCACTCCTTGTGTGTCTATGTCTGTTTTTAATAGTTCCAAAGATTTCTGCATTTTGGTTCAGCTTATTCCTAGACTCCTGTATCATGATGATAGCTCCTTTTTAGACAAATTTGAGCATCGGGTCCGCTGGAGAAGAGAACCCGTTACCTTAACTTTGGCAGTTCTATTAGGATTGGGAGTAGCGGCTGGAGTAGGTACAGGAACCGCTGCCTTAATTAAGACCCCCCAATACTATGAAGAACTACGTGCAGCTATGGATGTTGATCTTAGAACTATAGAACAGTCTATAACCAAATTAGAAGAATCTTTAACTTCCCTGTCCGAAGTGGTGCTACAGAATAGAAGGGGATTAGACTTATTATTCCTTAAAGAAGGAGGACTCTGTGCTGCCCTAAAAGAAGAATGTTGTTTTTATGTTGACCATTCAGGAGTAATCAAAGATTCTATGGCCAAACTTAGAGAACGCCTAGATATACGTAAAAGAGAAAGAGAAAGCCAACAAGGATGGTTCGAAAGCTGGTTTAATAAGTCCCCTTGGCTCACCACTCTCCTCTCCACCATAGCAGGACCTTTAATTACACTTATGCTTTTGCTTACTTTTGGCCCCTGCATCCTTAATAAGTTAGTAGCTTTTATTAGAGAAAGGATAAATGCAGTACAAGTTATGGTACTAAGGCAACAATATCGGGTCCTTCAGGAGGTTGAAAACTCGCTCTAAGATTAGAGCTATTTCTTAAAAAGAGTGGGGAATGAAGAATAAAAAATTACTGAACTCTTCCTCACCCCAGAGCCCGACCCCTCCCATCTAGAGATTGTTCCCAGAACACTCCTGAACTCTTCACCCCAGAATGCATTCCTGAACTCCTCACCCTAGAGTTCGAACCCTCCCAACTAAAGACTGTTCCAAGAACATTTTTGAGATAAGGGCCTCCTGGAACAACCTCAGAATGAACCGGGTACATTGCCAAATAATAGGACATGACCCCTTAGTTACGTAGAATTCCCTTGGCAGAACCCCTTGTCCCTTGGCAGAACCCCTTAGTTATGTAAACTTGTACTTTCCCTGCCCCGCTCTCCCCCCTTGAGTTTTCCTATATAAGCCTGTGAAAAATTTTGGCTGGTCGTCGATTCTCCTCTACACCACTAGGTGTATGAGTTTCGACCCCAGAGCTCTGGTCTATGTGCTTTCTTGCTGTTGCTTTATTAAATCTTGCCTTCAACA

**Corrected G2 consensus:**

>ConsG2

TGTTTAAATGGACCAATCATGTGAAACCGCGCCAATTCCTCCCCCAGCCCCACTCCTTTTCTATAAAAACCCCTAGCTTCCAAGCCTCGTGGTCGAATCCACTGTCTCCTGTTGTGTGAGATACGTTTCGACCCGGAGCTCCGCCATTAAAAAACCTCTTGTTGTTACATCAAGGTGTTGTGTTCTATTCGTGATTCTTGGGTGCACGCCGAATCGGGAGCTGAGTGGGGGTTTCCCCACTGAGTTCTTTCATTTGGAGGTCCCACCGAGATCTGCGTGACACCCAGGAACCCCGAAGGACCCCTTGGAGGTGCGTTTGTTTGTGTGAGTCTTGTTATGTTGTCTGTTGTCTAAGTGTCTAAGTGTGGCACTGCTGAATTTGTGTCTTAGTTTTTCAGTTCTGAGATTGTGGGTTTGAGCCCCACCTGTGTTACCAGTTCTGGTATTCTGTATTCTGGCAGCTGCCACTGCGGACCGTAAGGACCCTAGTGGCTGTGGGAAGACGACGGTCTATTTCCCCACAGGCTGCACCCTTGGAAGACATTCCGAGGGAGACCCTGGAGTGCCCGGGGTACGGAACAGTCAGGAGGACCTGGCTGTTGCCTGGCAGAGTGAAGAAGAGTGAGTGCTCTTCCTGCCAGAGGAGTGGAGCGGAATCCCACTCCATCAGAGGTAGCGTTTGGCTGGTTGTGTAAGTCCAGACGCAGACGAGTGTGCTTGGATGTCTTAGTGTTTTCCGTCTCTGTCATTGTGTTGTGTTTACTCTTATTCTTCACTATGGGACAGACCGTGTCTACTCCTTTATCTTTGACTAAGGACCATTGGACGGACGTTAGGGCTAGAGGACAAAATTTGTCAGTAAAAGTGAAGAAAAAGCCATGGATGACTTTCTGTTCCTCAGAATGGCCTGTTTTTGGAGTAGGTTGGCCAGCAGAAGGAACTTTTTACTTACCCACCATAAGGGCTGTGAAGGCCATTGTTTTTCAGGAAGGACCAGGGTCGCATCCAGACCAACAACCGTACATCATGGTATGGGAGGACTTGGCACGCTACCCACCCCCGTGGGTTCGCCCATTCCTCCCGCCTCTCCGCCCTGGCACCAAGATTCTAGCCATCCGAGAAAATGGTGAGAAAGAGAAACCGAAACCACCGCTCGGGAGAGATGATGATCGCAGCACACCAGTGACGAAACCCCCCAAGATCTATCCAGAGATTGAAGAACCCCCTGAGTGGCCCGAACCCCCTCAACCCCCACCGTATGCTCCCCAGCCCCAACCTTCAGCTCCCTCGGGACCCCTGCCTCAGGCCCCGGCCGGAGGAGGGGGTCCCTCCACAGGAACAAGGAGCCGGCGAGGAGTCACCCCTGAGGGGCCTGCGGATTCAACTGTGGCGCTCCCCCTCAGGGCTATTGGGGCTCCCCCTGCCGATCCAAATAGTCTACAGCCCCTACAGTATTGGCCTTTTTCCTCTTCTGACCTTTATAACTGGAAAGCTAATCACCCCCCTTTTTCAGAAAACCCTGCAGGACTCACTGGGTTGGTTGAATCATTAATGTATTCACACCAGCCGACCTGGGATGACTGCCAGCAGCTTCTGCAGACTCTATTCACAACCGAGGAGAGAGAGAGGATTCTCCTCGAGGCTCGGAAAAACGTCCGAGACGAGGCTGGGCGCCCTGTCCAAACTCCAGCTGAGATAGATGAAGGATTTCCGCTAACCCGGCCCCGATGGGATTATAATACGGCATCAGGTAGGGAACGACTGTCCAATTATCGCCGGGTCCTAGTGGCGGGTCTCAGAGGTGCTGCCCGGCAGCCCACGAATCTGGCCAAGGTAAGAGAGGTTATGCAGGGAGCGACTGAGCCCCCCTCAGTCTTCCTTGAAAGGCTCATGGAGGCTTATAGGAGATATACCCCATTCGACCCCACGTCTGAGGGTCAAAGGGCCTCAGTAATTATGGCCTTCATTGGCCAGTCAGCTCCTGACATTAGGAAGAAGTTACAGCGAATTGAGGGCTTGCAGGATTACACCATAAGGGATGTAGTTAGAGAGGCAGAGAAAGTGTATCATAGGAGAGAAACAGAAGATGAAAAGTTAGAGAGAGAGAAAAGAGAGAAAAGAGAAGAGGAGGATAGGAGAGACAGGAGGCAAGAAAAGGTTTTGACTAGGATCCTGGCCGCAGTAGGAGAAAGAGATAATGGAAGAAGAGGTAGACAGTCAGGGAACCTGGGAGACAAAAGGCAGCAGGGACCAAGGAGACCCAGAGAAGGCGGGCAGCGCCTGGAGAGGAACCAATGCGCGTATTGCAAGGAAATGGGCCACTGGAAGAGCGACTGTCCGAAAAAAAAACAAGAGGTAAAGGTGCTTTCTCTTGGAGAAGATGAAGACTAGGGGGAACGGGGCTCGGCCCCCCTCCCCGAGCCTAGGGTAACTTTAGAAGTGGAGGGGACCCCTGTGGACTTTCTAGTTGACACGGGAGCCGAATTTTCAGTACTCAAAACACCTCTAGGAAAAGTAAAGAAAAATGAAAAAACCTTGGTGATCGGGGCCACGGGACAAAAATCGTATCCATGGACCACATCCCGAGTAGTAGACATAGGGCGAAATCGAGTAACTCATTCGTTTCTAGTCATTCCAGAGTGTCCTATGCCTTTATTGGGGAGAGACTTACTAACCAAGTTAAAAGCACAAATAACTTTCACCTCTCATCGACCGGAGGTTTTCTGGGGAATAAAAGCGCCCCAGACTCTAGAGCTGTCTTTACAACTAGGGGAGGAATATCGACTTTACCAAAATAAAGTAAAGCCCCCTGAGGGATTACAGGACTGGTTGAATCGATACCCTCAGGCGTGGGCAGAGACGGGAGGAGTGGGGATGGCAAAACTGGTCCCCCCCGTGGTGATTGAACTTAAGTCCGGGGCCACCCCTATAGGGGTCCGACAATATCCCATGAGCAGAGAAGCTCAAGAGGGTATACGCCCCCAAATTAACAAACTGCTCCAACAAGGGATTTTGGTCCCATGCAAATCCCCTTGGAACACTCCTCTACTTCCAGTAAAAAAACCAGGGACCAGGGACTACCGTCCAGTACAGGACCTTAGAGAAGTCAACAAGAGAGTTCAGGACATACACCCCACGGTGCCAAATCCTTATAACCTCCTCAGCACCTTGCCACCTGGTCGGACATGGTACACAGTCCTGGATCTCAAAGAGCTTTTTTTCTGTTTGAGGTTACACCCCAACAGCCAGCCCTTGTTCGCTTTCGAATGGCGAGACTCCGAGAGTGGACAAGCCGGACAGCTCACATGGACGAGGCTGCCTCAGGGATTCAAGAACTCGCCCACTTTGTTCGATGAAGCCCTACACCGAGATCTTGCTCTTTTCCGAGCCAATAACCCACAGGTGACTCTTCTGCAATATGTAGATGACCTGCTCCTAGCTGCAGAAACACGCGAGGACTGTGAAATTGGGACCCAAAACCTCCTGGGCGAGTTAGGTAAGCTGGGGTATCGGGCCTCTGCTAAAAAGGCTCAGTTATGCCAGATAGAAGTGACCTACCTAGGATATGTCTTGAGAGATGGACAACGGTGGCTCACAGAAGCCAGAAAACAAGCTGTTATGCAGATCCCGACCCCAACCACTGCTCGCCAGGTAAGAGAGTTCCTGGGGACCGCCGGGTTTTGCAGACTCTGGATTCCCGGATTTGCCACACTGGCAGCTCCCTTGTATCCACTAACCAAAGAGAAAGGGGAATTCACCTGGACCAGAGAACATCAGCTAGCCTTTGAAACTCTCAAAAAGGCACTGCTGCAGGCTCCGGCATTGGCCCTGCCAGATTTAAACAAACCTTTCACCCTATACATTGATGAAAGAAATGGAGTGGCAAGGGGAGTCCTTACCCAGGTTTTGGGACCATGGAAGCGCCCGGTAGCCTACTTATCAAAGAAACTGGACGCTGTGGCCAGTGGATGGCCCTCCTGCCTGCGAGCGATAGCAGCCACGGCTGTGCTAGTAAAAGATGCTGACAAACTGACTATGGGCCAGAATGTTACTATAGTGGCCCCACACTCTCTTGAGAGCATCATCAGGCAACCACCGGACCGCTGGATGACCAACGCCCGAATGACGCACTACCAGAGCCTATTGCTGACAGAGCGAGTAAGTTTTGCACCCCCAGCCATTCTCAACCCCGCCTCCTTACTACCTGAGGCTGACGAGGCCCCTGCACATAAGTGTGAAGAAATACTGGCAGAAGAGACTGGAATCCGGCCAGACCTCACAGACCAACCTTGGCCAGGGGCGATGACTTGGTTCACGGACGGAAGCAGCTTTGTGGTAGAAGGTAAGCGGAAGGCTGGGGGCGCAGTAGTGGATGGAAAGGCTGTCATATGGGCCAGCAGTCTGCCGGAGGGTACATCAGCTCAAAAAGCGGAACTAATCGCATTAATTCAAGCCTTAAGGCTGGCAGAAGGAAGGGCTCTTAATGTCTATACCGACAGCCGGTACGCTTTTGCCACGGCTCATGTTCACGGAGCAATATACCGACACCGTGGACTGCTGACGTCTGCCGGCAAAGATATCAAAAATAAAGAAGAAATTCTCAGCTTATTAGAAGCTGTTCATTTGCCCCGTAGGGTGGCAATTATCCATTGCCCAGGACACCAGAAGGGAACTGGGCCCGTTGAAAAGGGAAATCAAATGGCAGACCAAGAAGCTAAAAAAGCAGCCCAAGGGCCAATGACTCTGGTGGTGAGAACCCAACAGCCCGCTGCTGAGGAAATAAATAAAAGAACCCTCACAGAAGAAGAGGGGCGAGATTACTTAGCTAACATACACCATCTGACTCATTTAGGAACTAAAAAATTACTAAAATTGGTTAGTAAGTCCCCCTATTACATTCCTGGATTAAAAGGAATTGTGGAAGAGATAGTAAAAAACTGCCGTGCTTGTGCACTTACCAACGCTGGGTCTAGCAGGCTCCAGGAAGGAAAACGACTGCGAGGAGACAGACCTGGAGCCTACTGGGAAACTGACTTCACTGAGGTGAAACCGGCTAGGTATGGAAATAAATATCTCCTAGTTTTTATAGACACCTTTTCAGGATGGGTCGAAGCGTTCCCCACCAAGAAAGAAACGGCTAATGTAGTGGTCAAGAAGATACTTGAAGAAATCCTTCCCCGCTTTGGGATACCTAAGGTAATGGGGTCAGACAACGGACCTGCCTTCGTCTCCCAGGTAAGTCAGGGATTGGCCAGACAACTGGGGACAAATTGGAAATTACATTGTGCATACAGACCCCAGAGTTCAGGACAGGTAGAAAGGATGAACAGAACGCTAAAGGAGACTCTGACTAAAATAGCCTTAGAATCCGGCGGAAGCGATTGGACAGCCGTTCTCCCTTATGCCTTGTTCAGGGTTCGGAATACACCTGGACCCCTTGGCCTAACTCCATTTGAATTAATGTATGGGGCGCCCCCACCCATTTTTATGACCGTAGGGGATAAGAATCGCCCGGATGTGTCTTTCTCTCCTTCTTCTAGTCTTTTGGCTCGATTAAAAGCTCTCGAAATAGTAAGAAAAGAGGTCTGGGAACAGCTAAAAGAAACCTATGTTGCTGGTGACACACAGGTGCCGCATCAGTTTGAAGTAGGAGACGCAGTCCTGGTGAGGAGACACCGAGCGGGAAACCTAGAACCGAGGTGGAAGGGACCCTACTTGGTGCTACTGACAACGCCCACCGCGGTCAAAGTGGAAGGAATCCCCACTTGGGTCCACGCATCCCACGTCAAGAGAGCACCCCCTGGAGTCAGCCATGATGAGTGGACTTTGGAGAAGACTACTAATCCTTTTAAGTTGCGCCTGCTTCGTAGGAGCGATCCCAAAAGACTTCAACCCCCACAGTCCTGTTCAACAAACGTGGGAGGTACTCAATGAGGGGGGTAGGGCTGTATGGACAATCGCCGAGGTACACCCTCTGTGGACTTGGTGGCCTGATCTTTTCCCTGACATCTGTAAGTTGGCTATAGGAGCCCCTCCTGGATGGGACTTGGAGGGGTACTCTGACATTCAGAGGGCACCTTTAACACCCCCTCCGTACGTAGAAAAACATTCGAGAGACCCATGGGGTGGTTGCTCTAACCAAAGGGATAGAAGTATGCTTCGAACCCATCCCTTCTATGTCTGCCCCGGGCCCCACCGAAGTCAGTCCCTCAATCCAACGTGTGGAGGTAAGGCTGACTTCTTTTGTAAGAGCTGGGGTTGCGAGACTTCAGGTACAGCCCGCTGGAAGCCCTCCTCGAGCTGGGACTATATTAGAGTAACAGCCAACTATTCCCTAGCGTCTTATGTACCTGGAGGATTTGACCTAGACGAGTGTACTGACTGGTGCCATCCGCTCCGTGTCACCTTCACTGAACCAGGGAAGAGAGCTCTGGGATGGACAAAAGGGTATACCTGGGGTCTTAGGATTTACAAGGAAAGATATGATGAGGGATTATTGTTCACTATCAGATTAAAAATAGAGACCCCCTACAATCCTTTAGGCCCCCCAACCAAGTTCACACCCCTCACCCATACAATTACTCAGCCTACTCCAGTGATTGCGGACCCCCTTAATATGGCCGCTATCACCCAACCTCCCACTCCTCAGGTACCTCTAACTATTACCCCCGCGATTCCTTCAAGACAGAGGATGTTTAACCTAGTGAGAGGAGCCTTTTATGCCCTTAACAGAACTGATCCAAGCGCTACTGAGGACTGCTGGCTATGCCTGTCCTCGGGTCCGCCTTATTATGAAGGAATCGCCTTCAATGGAGATTTCAACAGAACCAGCAGCCATACTTCCTGCTCTTGGGGTACAGGACAAAAACTGACCCTGACTGAAGTATCCGCGAGGAATCCAGGTCTCTGTATAGGTACCCCACCTTCCACTCACAAACACCTATGCGGACAAATTCAGTCCGTGTCCAGAACGGAAGCTAATTACTATCTTGTACCTTCCCCGGTTGGATGGTGGGCTTGCAATACAGGACTTACTCCCTGTGTATCAACTAAGGTTTTTAATTCATCTCATGATTTTTGTGTCATGATCCAGCTGTTACCCCGTGTATATTATCACCCTGCATCCAGTTTAGAAGAAAGCTATGCTGGCCGGCGGTCAAAAAGAGAACCAATTACTTTAACCCTGGCTGCATTCATGGGAATAGGTATGGCAGTAGGAGTGGGGACGGGAGTGTCAGCTTTGATAGAAGGAAGACAGGGAATTCAGTCTTTGAGGGATGCTGTCAATGAAGACCTAGCGGCAATAGAGAAGTCCATTGACGCTTTAGAAAAATCTTTGACCTCCCTGTCTGAGGTAGTTTTACAGAACAGGAGAGGTCTTGATTTGTTGTTCCTAAAGGAAGGAGGACTGTGTGCTGCCCTTAAAGAAGAGTGCTGCTTCTATGCAGATCATACAGGAATAGTTAGAGACTCTATGCAGAAACTGAGAGAAAGATTAGAGCGAAGGAAACGGGAACGGGATGCTCAACGGGGGTGGTTTGAGTCGTGGTTTGAATCACGACCATCTTGGATAACTTCTTTAATTTCCGCTGTAGCCGGACCAATCCTTATGATATGCTTAGCTTTAGTTTTCGGCCCTTGTATAATAAATAGAGGAATGGCTTTCATCCAGAGTAAAATTGATACAGTAAAACTCATGGTTCTTCAAAGGCAATATCAACCTATAGTTCAGGTAGATGAAGAGTTAGGGGACACCAATCTCTAAAATTCTATGATTAGAATTAGTCTAAACAGAAGAAGAGGGGAATGAAAGGAAATAAAACTGTAATTCATGTAATGTATGTTAAATAGCCCAAAGAGTTGTTTCTGAGCTTTGAAACCTGGGGCTGAGAACATAGCAGAACAGACCAGGACATGCCCGGGCAAGCCCATCGCCTCCCTAGCTCCCACCCCTCTGACCTAAGTTAAATGTTACAGGCTGCTGATGTTTAAATGGACCAATCATGTGAAACCGCGCCAATTCCTCCCCCAGCCCCACTCCTTTTCTATAAAAACCCCTAGCTTCCAAGCCTCGTGGTCGAATCCACTGTCTCCTGTTGTGTGAGATACGTTTCGACCCGGAGCTCCGCCATTAAAAAACCTCTTGTTGTTACATCAAGGTGTTGTGTTCTATTCGCGATTCTTGGGTGCACGCCGAATCGGGAGCTGAGTGGGGGTTTCCCCA

**Corrected G3 consensus:**

>ConsG3

TGGGCCCCCCATGACTGCCTCGAGATCTTGGCTGAGACACACGGAACCAGACCGGACCTCACGGACCTAGCCCCTCCCAGACGCCCAAGGATCTGGGCACTCCTTGAACCTGTGTGTCTGCCAATGTTCTGACCAGTTGTGTGCCCATTGTTGAACCTTCATTAGACCCTTTCCTCGTACCCCTCCCATACCCATTTCTTGAAAATAGACATTGTTTAGAACTAAAAAGTCCCACCTCAGTTTCCCCAAATGACCGAGAAATACCCCAAGCCTTATTCGAACTAACCAACCAGCTCGCTTCTCGCTTCTGTAACCGCGCTTTTTGCTCCCCAGCCCCAGCCCTATAAAAAGGGTAAAAACTCCACACTCGGCGCGCCAGTCCTCCGATAGACTGAGTCGCCCGGGTACCCGTGTTCCCAATAAAGCCTCTTGCTGTTTGCATCCGAATCGTGGTCTCGCTGATCCTTGGGAGGGTCTCCTCAGATTGATTGACTACCCACCTCGGGGGTCTTTCATTTGGAGGTCCCACCGAGATTTGGAGACCCCTGCCCAGGGACCACCGACCCCCCGCCGGGAGGTAAGCTGGCCAGCGGTCGTTTCGTGTCTGTCTCTGTCTTCGTGCGTGTTTGTGCCGGCATCTAATGTTTGCGCCTGCGTCTGTACTAGTTGGCTAACTAGATCTGTATCTGGCGGTTCCGCGGAAGAACTGACGAGTTCGTATTCCCGGCCGCAGCCCCTGGGAGACGTCCCAGCGGCCTCGGGGGCCCGTTTTGTGGCCCATTCTGTATCAGTTAACCTACCCGAGTCGGACTTTTTGGAGCTCCGCCACTGTACGTGGCTTTGTTGGGGGACGAGAGACAGAGACACTTCCCGCCCCCGTCTGAATTTTTGCTTTCGGTTTTACGCCGAAACCGCGCCGCGCGTCTGATTTGTTTGTTGTTCTTTTGTTCTTCGTTAGTTTTCTTCTGTCTTTAAGTGTTTTCGAGATCATGGGACAGACCGTAACTACCCCTCTGAGTCTAACCTTGCAGCACTGGGGAGATGTCCAGCGCATTGCATCCAACCAGTCTGTGGATGTCAGGAAGAGGCGCTGGATTACCTTCTGTTCCGCCGAATGGCCAACTTTCAATGTGGGATGGCCTCAGGATGGTACTTTCAATTTAAGTATTATCTCTCAGGTTAAGTCTAGAGTGTTTTGTCCTGGTCCCCACGGACACCCGGATCAGGTCCCATATATCGTCACCTGGGAGGCACTTGCCTATGACCCCCCTCCGTGGGTCAAACCGTTTGTGTCTCCTAAACTTCCTCCCTTGCCGACAGCTCCCGTCCTCCCGCCCGGTCCTTCTGCGCAACCTCCGTCCCGATCTGCCCTTTACCCTGCCCTTACCCCCTCTATAAAGTCCAAACCTCCTAAGCCCCAGGTTCTCCCTGATAGCGGCGGACCTCTCATTGACCTTCTCACAGAGGACCCCCCGCCGTACGGAGCACAACCTTCCTCCTCTGCCAGAGAGAACGACGAAGAAGAGGCGGCCACCACCTCCGAGGTTTCCCCCCCTTCTCCCATGGTGTCTCGACTGCGGGGAAGGAGAGACCCTCCCGCAGCGGACTCCACCACCTCCCAGGCATTCCCACTCCGCATGGGGGGAGATGGCCAGCTTCAGTACTGGCCGTTTTCCTCCTCGGATTTATACAATTGGAAAAATAATAACCCTTCCTTTTCTGAAGATCCAGGTAAATTGACGGCCTTGATTGAGTCCGTCCTCATCACCCACCAGCCCACCTGGGACGACTGTCAGCAGTTGTTGGGGACCCTGCTGACCGGAGAAGAAAAGCAGCGGGTGCTCCTAGAGGCTAGAAAGGCAGTCCGGGGCAATGATGGACGCCCCACTCAGTTGCCTAATGAAGTCAATGCTGCTTTTCCCCTTGAACGCCCCGATTGGGATTACACCACTACAGAAGGTAGGAACCACCTAGTCCTCTATCGCCAGTTGCTCTTAGCGGGTCTCCAAAACGCGGGCAGAAGCCCCACCAATTTGGCCAAGGTAAAAGGGATAACCCAGGGACCTAATGAGTCTCCCTCAGCCTTTTTAGAGAGACTCAAGGAGGCCTATCGCAGGTACACTCCTTATGACCCTGAGGACCCAGGGCAAGAAACCAATGTGTCTATGTCATTCATCTGGCAGTCTGCCCCGGATATCGGGCGAAAGTTAGAGCGGTTAGAAGATTTAAAGAGCAAGACCTTAGGAGACTTAGTGAGGGAAGCTGAAAAGATCTTTAATAAGCGAGAAACCCCGGAAGAAAGAGAGGAACGTATCAGGAGAGAAACAGAGGAAAAAGAAGAACGCCGTAGGGCAGAGGATGAGCAGAGAGAGAAAGAAAGGGACCGCAGAAGACATAGAGAGATGAGCAAGCTCTTGGCCACTGTAGTTATTGGTCAGAGACAGGATAGACAGGGGGGAGAGCGGAGGAGGCCCCAACTTGATAAGGACCAATGCGCCTACTGCAAAGAAAAGGGACACTGGGCTAAGGACTGCCCAAAGAAGCCACGAGGGCCCCGAGGACCGAGGCCCCAGACCTCCCTCCTGACCTTAGGTGACTAGGGAGGTCAGGGTCAGGAGCCCCCCCCTGAACCCAGGATAACCCTCAAAGTCGGGGGGCAACCCGTCACCTTCCTGGTAGATACTGGGGCCCAACACTCCGTGCTGACCCAAAATCCTGGACCCCTAAGTGACAAGTCTGCCTGGGTCCAAGGGGCTACTGGAGGAAAGCGGTATCGCTGGACCACGGATCGCAAAGTACATCTAGCTACCGGTAAGGTCACCCACTCTTTCCTCCATGTACCAGACTGCCCCTATCCTCTGCTAGGAAGAGACTTGCTGACTAAACTAAAAGCCCAAATCCACTTCGAGGGATCAGGAGCTCAGGTTGTGGGACCAATGGGACAGCCCCTGCAAGTGCTGACCCTAAACATAGAAGATGAGTATCGGCTACATGAGACCTCAAAAGAGCCGGATGTTTCTCTAGGGTCCACCTGGCTTTCTGATTTTCCCCAGGCCTGGGCGGAAACCGGGGGCATGGGACTGGCAGTTCGCCAAGCTCCTCTGATCATACCTCTGAAGGCAACCTCTACCCCCGTGTCCATAAAACAATACCCCATGTCACAAGAAGCCAGACTGGGGATCAAGCCCCACATACAGAGACTGTTGGACCAGGGAATACTGGTACCCTGCCAGTCCCCCTGGAACACGCCCCTGCTACCCGTTAAGAAACCAGGGACTAATGATTACAGGCCTGTCCAGGATCTGAGAGAAGTCAACAAGCGGGTGGAAGACATCCACCCCACCGTGCCCAACCCTTACAACCTCTTGAGCGGGCTCCCACCGTCCCACCAGTGGTACACTGTGCTTGATTTAAAGGATGCCTTTTTCTGCCTGAGACTCCACCCCACCAGTCAGCCTCTCTTCGCCTTTGAGTGGAGAGATCCAGAGATGGGAATCTCAGGACAATTGACCTGGACCAGACTCCCACAGGGTTTCAAAAACAGTCCCACCCTGTTTGATGAGGCACTGCACAGAGACCTAGCAGACTTCCGGATCCAGCACCCAGACTTGATCCTGCTACAGTACGTGGATGACTTACTGCTGGCCGCCACTTCTGAGCTCGACTGCCAACAAGGTACTCGGGCCCTGTTACAAACCCTAGGGGACCTCGGGTATCGGGCCTCGGCCAAGAAAGCCCAAATTTGCCAGAAACAGGTCAAGTATCTGGGGTATCTTCTAAAAGAGGGTCAGAGATGGCTGACTGAGGCCAGAAAAGAGACTGTGATGGGGCAGCCTACTCCGAAGACCCCTCGACAACTAAGGGAGTTCCTAGGGACGGCAGGCTTCTGTCGCCTCTGGATCCCTGGGTTTGCAGAAATGGCAGCCCCCTTGTACCCTCTCACCAAAACGGGGACTCTGTTTAATTGGGGCCCAGACCAGCAAAAGGCCTATCAAGAAATCAAACAGGCTCTTCTAACTGCCCCAGCCCTGGGATTGCCAGATTTGACTAAGCCCTTTGAACTCTTTGTCGACGAGAAGCAGGGCTACGCCAAAGGCGTCCTAACGCAAAAACTGGGACCTTGGCGTCGGCCGGTGGCCTACCTGTCCAAAAAGCTAGACCCAGTGGCAGCTGGGTGGCCCCCTTGCCTACGGATGGTAGCAGCCATTGCCGTTCTGACAAAAGATGCAGGCAAGCTAAACATGGGACAGCCGCTAGTCATCCTGGCCCCCCATGCAGTAGAGGCACTGGTCAAGCAACCCCCTGACCGCTGGCTATCCAACGCCCGCATGACCCACTATCAGGCGATGCTCCTAGATACGGACCGGGTTCAGTTCGGACCGGTGGTAGCCCTAAACCCGGCCACGTTGCTCCCCCTACCGGGGAAAGAGCCTCACCATGACTGCCTCGAGATCTTGGCCGAGACACACGGAACCAGACCAGACCTCACGGACCAGCCCCTCCCAGACGCCGACCACACCTGGTATACAGATGGAAGCAGCTTCCTGCAAGAGGGACAACGTAAGGCTGGAGCAGCGGTGACCACCGAGACCGAGGTAATCTGGGCCAAGGCGTTGCCAGCCGGGACATCCGCCCAGCGAGCTGAACTAATAGCACTCACCCAGGCCCTAAAGATGGCAGAAGGTAAGAAGCTAAATGTTTATACTGATAGCCGCTATGCCTTTGCTACCGCCCATGTCCATGGAGAAATATATAGGAGACGTGGGTTGCTCACCTCAGAAGGCAAGGAGATCAAGAACAAGGGCGAAATCTTGGCCTTACTGAAAGCTCTCTTTCTGCCCAAAAGACTCAGTATAATTCACTGCCCAGGACATCAGAAAGGCAATAGTGCTGAAGCTAAAGGCAACCGAATGGCGGACCAGGCAGCCCGGGAAGCAGCCATGGGGACTGACACAAAGGCCTCCTCACTTCTCATAGAGACCTCAACCCCGTACACTCCAGACTTCTTCCATTATACTGAGACAGATATAAAGAACCTACAAGAGTTGGGAGCCACATATGATAGAGAGAAAAAATATTGGGTCCTGCAAGGTAAACCTGTGATGCCTGACCAGTTCACCTTTGAATTATTAGACTTCCTTCACCAGCTCACCCACCTTAGCTATCAGAAGATGAGGGCACTTCTAGACAGAAAAGAAAGCCCCTATTACATGCTAAATAAAGATAAGATCCTCCACGAGGTGGCGGAATCATGCCAAGCCTGTGTCCAAGTAAATGCCAGTAAAGCTAAGATCGGGGCCGGGGTGCGAGTAAGAGGACATCGACCAGGCACCCATTGGGAAATTGACTTTACTGAAGTGAAGCCCGGACTGTATGGGTACAAGTATCTCCTGGTGTTCGTGGACACGTTCTCTGGCTGGGTTGAAGCCTTCCCAACCAAGCATGAGACTGCCAAAGTTGTGACCAAGAAGCTTCTGGAAGAAATATTTCCAAGGTTTGGAATGCCCCAAGTATTGGGGACTGATAATGGGCCTGCCTTCGTCTCCCAGGTAAGTCAGTCGGTGGCCAAGCTACTGGGGATTGATTGGAAACTACATTGTGCTTACAGACCCCAGAGTTCAGGTCAGGTAGAAAGAATGAATAGGACAATCAAGGAGACTTTGACCAAATTAACGCTTGCAACTGGCACTAGAGACTGGGTACTCCTACTTCCCTTGGCCCTCTACCGAGCCCGCAACACTCCGGGCCCCCATGGACTCACTCCGTATGAAATCCTGTATGGGGCGCCCCCGCCCCTTGTTAATTTCCATGATCCTGAAATGTCAAAGTTTACTAATAGCCCCTCTCTCCAAGCTCACTTACAGGCCCTCCAAGCAGTACAACGAGAGGTCTGGAAGCCACTGGCCGCTGCCTATCAGGACCAGCTGGACCAGCCAGTGATACCACACCCCTTCCGTGTCGGCGACACCGTGTGGGTACGCCGGCACCAGACTAAGAACTTGGAACCTCGCTGGAAAGGACCCTACACCGTCCTGCTGACCACCCCCACCGCTCTCAAAGTAGACGGCATCGCTGCGTGGATCCACGCCGCTCACGTAAAAGCGGCGACAACCCCTCCGGCCGGAACAGCATCAGGACCGACATGGAAGGTCCAGCGTTCTCAAAACCCCTTAAAGATAAGATTAACCCGTGGGGCCCCCCTATAGTCCTGGGGATCTTAATAAGGGCAGGAGTATCAGTACAACATGACAGCCCTCATCAGGTCTTCAATGTTACTTGGAGAGTTACCAACTTAATGACAGGACAAACAGCTAATGCTACCTCCCTCCTGGGGACAATGACCGATGCCTTTCCTAAACTGTACTTTGACTTGTGCGATTTAATAGGGGACGACTGGGATGAGACTGGACTCGGGTGTCGCACTCCCGGGGGAAGAAAAAGGGCAAGAACATTTGACTTCTATGTTTGCCCCGGGCATACTGTACCAACAGGGTGTGGAGGGCCGAGAGAGGGCTACTGTGGCAAATGGGGCTGTGAGACCACTGGACAGGCATACTGGAAGCCATCATCATCATGGGACCTAATTTCCCTTAAGCGAGGAAACACCCCTCGGAATCAGGGCCCCTGTTATGATTCCTCAGCGGTCTCCAGTGGCATCAAGGGCGCCACACCGGGGGGTCGATGCAATCCCCTAGTCCTGGAATTCACTGACGCGGGCAAAAAGGCCAGCTGGGATGGCCCCAAAGTATGGGGACTAAGACTGTACCGATCCACAGGGACCGACCCGGTGACCCGGTTCTCTTTGACCCGCCAGGTCCTCAATATAGGGCCCCGCGTCCCCATTGGGCCTAATCCCGTGATCACTGACCAGTTACCCCCCTCCCGACCCGTGCAGATCATGCTCCCCAGGCCTCCTCAGCCTCCTCCTCCAGGCGCAGCCTCTATAGTCCCTGAGACTGCCCCACCTTCTCAACAACCTGGGACGGGAGACAGGCTGCTAAACCTGGTAGATGGAGCCTACCAAGCTCTCAACCTCACCAGTCCTGACAAAACCCAAGAGTGCTGGTTGTGTCTGGTATCGGGACCCCCCTACTACGAAGGGGTTGCCGTCCTAGGTACTTATTCCAACCATACCTCTGCCCCAGCTAACTGCTCCGTGGCCTCCCAACACAAGCTGACCCTGTCCGAAGTGACCGGACAGGGACTCTGCGTAGGAGCAGTTCCCAAAACCCATCAGGCCCTGTGTAATACCACCCAGAAGACGAGCGACGGGTCCTACTATCTGGCTGCTCCCGCCGGGACCATTTGGGCTTGCAACACCGGGCTCACTCCCTGCCTATCTACCACTGTACTCGACCTCACCACCGATTACTGTGTCCTGGTTGAGCTCTGGCCAAAGGTGACCTACCACTCCCCTGGTTATGTTTATGGCCAGTTTGAGAGAAAAACCAAATATAAAAGAGAGCCGGTGTCATTAACTCTGGCCCTGCTGTTGGGAGGACTTACTATGGGCGGCATAGCTGCAGGAGTAGGAACGGGGACTACAGCCCTAGTGGCCACCAAACAATTCGAGCAGCTCCAGGCAGCCATACATACAGACCTTGGGGCTTTAGAAAAGTCAGTCAGTGCCCTAGAAAAGTCTCTGACCTCGTTGTCTGAGGTGGTCCTACAGAACCGGAGGGGATTAGATCTACTGTTCCTAAAAGAAGGAGGATTATGTGCTGCCCTAAAAGAAGAATGCTGTTTCTACGCGGACCACACTGGCGTAGTAAGAGATAGCATGGCAAAGCTAAGAGAAAGGTTAAACCAGAGACAAAAATTGTTCGAATCAGGACAAGGGTGGTTTGAGGGACTGTTTAACAGGTCCCCATGGTTCACGACCCTTATATCCACCATTATGGGCCCCTTGATAATACTTTTATTAATCCTACTCTTCGGACCCTGTATTCTCAACCGCTTGGTCCAGTTTGTAAAAGACAGAATTTCGGTAGTGCAGGCCCTGGTTTTGACCCAACAGTATCACCAACTCAAATCAATAGATCCAGAAGAAGTGGAATCACGTGAATAAAAGATTTTATTCAGTTTCCAGAAAGAGGGGGGAATGAAAGACCCCACCATCAGGCTTAGCAAGCTAGCTGCAGTAACGCCATTTTGCAAGGCATGAAAAAGTACCAGAGCTGAGTTCTCAAAAGTTACAAGAAAGTTCAGTTAAAGATTAACAGTTAAAGATTAAGGCTGAATAATACTGGGACAGGGGCCAAATATCGGTGGTCAAGCACCTGGGCCCCGGCTCAGGGCCAAGAACAGATGGCTCTCAGACGTCAGTGTTAGCAGAACTAGCTTCACTGATTTAGAAAAATAGAGGTGCACAGTGCTCTGGCCACTCCTTGAACCTGTGTGTCTGCCAATGTTCTGACCAGTGTGTGCCCATTGTGAACCTTCATTAGACCTTTCCTGTACCCCTCCCATACCCATTTCTTGAAAATAGACATTGTTTAGAACTAAAAAGTCCCACCTCAGTTTCCCCAAATGACCGGAAAATACCCCAAGCCTTATTCGAACTAACCAACCAGCTCGCTTCTCGCTTCTGTAACCGCGCTTTTTGCTCCCCAGCCCCAGCCCTATAAAAAGGGTAAAAACTCCACACTCGGCGCGCCAGTCCTCCGATAGACTGCGTCGCCCGGGTACCCGTGTTCCCAATAAAGCCTCTTGCTGTTTGCATCCGAATCGTGGTCTCGCTGGTCCTTGAGAGGGTCTCCTCAGATTGATTGACTACCCA

Consensus amino acid sequences for group G1:

Pre-Gag ORF:

<Group G1 had an in-frame ORF ahead of Gag>

lwrprdhlglrdrgfesptscrceivgssptsrfvarswvrvpplflapccetvgssptsvcvtgswvrvppraeglnrpalerpsdslsclwstrsrrrfwflfclslvfalvvstii

Gag:

MGQSVSTPLSLTLeHWKEVqvRAHNQSVEVRKGPWQTFCaSEWPTFGVGWPPEGAFDLSLIaAVrRIVFQEeGGHPDQiPYIVTWQnLVQfPPPWVKPWtPNSSKLTVAVAQSdAagkSgPSAPPkIYPEIDDLLWMdSQPPPYPLPQQPPAAAPPqGPiARGAqGpAGGTRSRRGRSpGEEGGPDSTVALPLRAhVGGPAPGPNdLIPLQYWPFSSSDLYNWKTNHPPFSENPsGLTGLLESLMFSHQPTWDDCQQLLQVLFTTEERERILmEARKNVLGEDGTPTaLPNLVDEAFPLNRPNWDYNTAEGRgRLLVYRRTLVAGLRGAARRPTNLAKVREVLQGqTEPPSVFLERLMEAYRRYTPFDPLSEGQRAAVAMAFIGQSAPDIKKKLQRLEGLQDHTLQDLVKEAEKVYHKRETEEERQEREKKEmEEREnRRDRRQERNLSKILAAVVndRqSGKGKIGlLGNRAvKpPGGRKIPLeKDQCAYCKEKGHWARDCPKNRERsKVLTLEDD

Pro:

GSrGSDPlPEPRVTLsVeGtPVnFLIDTGAEHSVLTsPLGkLgSKKTmVIGATGSKfYPWTTeRalQInKniVTHSFLViPECPaPLLGRDLLTKLKAQvqFTsEG

Pol:

PQVsWGkaPvaCLVLNtEEEYRLHEEqPKnaVSSgWLtaFPnVWAEqAGMGLAkQVPPVVVELKAdATPISVRQYPMSKEAREGIRPHIQRLLDQGVLVaCQSPWNTPLLPVrKPGTNDYRPVQDLREVNKRVlDIHPTVPNPYNLLSSLPPerTWYTVLDLKDAFFCLRLHPkSQLLFAFEWRDPEgGqTGpTnWTRLPQGFKNSPTLFDEALHRDLAPFRARNPQLtLLQYVDDLLVAAASkELChQGTeRLLaELSDLGYRVSAKKAQICQtEVTYLGYtLrgGKRWLTEARkKTVMmIPsPTTPRQVREFLGTAGFCRLWIPGFAtLAAPLYPLTKEGvPFeWkEEHQrAFEaIKssLmTAPALALPDLTKPFvLYVDERAGVARGVLTQaLGPWKRPVAYLSKKLDPVASGWPTCLKAiAAVALLiKDADKLTMGQqVTVvAPHALESIVRQPPDRWMTNARMTHYQSLLLNERVtFAPPAiLNPATLLPLtndSvPVHqCmDILAEETGTRRDLTDQPwPGAPsWYTDGSSFLiEGKRKAGAAVVDGKkVIWASALPEGTSAQKAELIALiQALReAKGKIvNIYTDSRYAFATAHIHGAIYRQRGLLTSAGKDIKNKEEILALLEAIHaPkKVAIIHCPGHQRGeDlVAKGNRMADsvAKQVAQgamILTEkgdPPKsPEDerynIkELLwTsdPLPYffEGKiELTPEeGiKFvKgLHQfTHLGvEKMmrLikNSrYqvPNLkSvAqKIidSCKpCAfTNATrAyKePGKRQRGDRPGVYWEVDFTEVKPGMYGNKYLLVFVDTFSGWVEgFPTKTETAqIVAKKILEEILPRFGIPKViGSDNGPAFVAQVSQGLATQLGIDWKLHCAYRPQSSGQVERMNRTlKETLTKLAiETGGKDWVaLLPLALfRARNTPGRFGLTPFEvLYGGPPPLmEaGgTLvSDsDpvLPSslLiHLKALEvIRTQIWDQLKAAYtPGTTaVPHgFRVGDkVLVRRHRTgSLEPRWKGPYLVLLTTPTAVKVDGIAsWIHASHVKrAAsQDEEnhEdnwTVaaTDNPLKLRLRRRRhpeprep

Env:

<a long non-ORF stretch (not shown) preceeds the consensus Env>

vglasihstkrswvhfhdqtdserpggdLVGPNKVLIEQGPPVVPAPPKVPAVPAPPTPQPNTVVPSLGTNTPLIKPTLASPPPLGTENRLVSLVQGAFLALNrTNPnmTQSCWLCyaSSPPYYEGIAqiRTYNITsDhSqCLWGenrKLTLaaVSGrRALLGqVPQDKgHLCNQTQNIQSSkSGQyLVPPldTVWACNTGLTPCvSmSVFNSskDFCILVQLIPRlLYHDDSSfLDKFEhRVrwrREPVtLTLAVLLGLGVAAGVGTGTAALIKTpQYYeeLrAAmdVDLrTIEQSITKLeESLTSLSEVVLQNRRGLDLLFLKEGGLCAALKEECCFYVDHSGVIKdSMAKLRERLDIRKrERESQqGWFEsWFNkSPWLTTLLSTIAGPLITLmLLLTFGPCILNKLVAFIRErINAVqvmvlrqqyrvlqevens

Consensus amino acid sequences for group 2:

Gag:

MGQTVSTPLSLTkDHWTDVRARGQnLSVkVKKKPWmTFCSSEWPvFGVGWPaEGTFyLpTIrAVKAIVFQEGPGSHPDQqPYImVWEDLARyPPPWVRPFLPPLrPGTKiLAIrENGeKEKPKPPlgRDddrsTPVTkPPkIYPEIEEPPeWPEPPQPPPYAPQPqPsAPSGPLPqAPAGGGGPStGTRSRRGvTPEGPADSTVALPLRAIGAPPADPNSLQPLQYWPFSSSDLYNWKANHPPFSENPAGLTGLVESLMySHQPTWDDCQQLLQTLFTTEERERILLEARKNVRDEAGRPvQtPAEIDEGFPLTRPrWDYNTAsGRERLSnYRRvLVAGLRGAARqPTNLAKVREVMQGATEPPSVFLERLMEAYRRYTPFDPTSEGQRASVIMAFIGQSAPDIRKKLQRIEGLQDYTIRDVvREAEKVYHRRETEdEKlEREKREKREEEDrRDRRQEKvLTRILAAVGERDnGRRgRQSGNLgdkRqQGPRRPREGGQrLeRNQCAYCKEmGHWKSDCPKKKQEVKVLsLGEDED

Pro:

gErGSaPlPEPRVTLEVeGtPVDFLVDTGAEfSVLkTPLGkVkKNeKTLVIGATGQKsYPWTTSRvVdIGrnRVTHSFLViPECPmPLLGRDLLTKLKAQItFTsH

Pol:

PeVfWGikaPqtLeLsLQLgEEYRLyqnKvKPPEgLqDWLNryPQAWAETGGvGMAklVPPVVIELKSGATPIgVRQYPMSrEAqEGIRPqInKLLqQGILVPCkSPWNTPLLPVKKPGTrDYRPVQDLREVNKRVQDIHPTVPNPYNLLSTLPPgrTWYTVLDLKelFFCLRLHPNSQPLFAFEWRDsESGqagQLTWTRLPQGFKNSPTLFDEALHRDLAlFRAnNPQVtLLQYVDDLLLAAeTREDCEiGTQnLLgELGKLGYRASAKKAQLCQiEVTYLGYvLrDGQRWLTEARKqAVMqIPTPTTaRQVREFLGTAGFCRLWIPGFAtLAAPLYPLTKEkGeFTwTrEHQLAFEtlKKALLqAPALALPDLnKPFTLYIDERnGVARGVLTQvLGPWKRPVAYLSKKLDaVASGWPsCLRAiAAtAVLVKDADKLTMGQNVTIvAPHSLESIiRQPPDRWMTNARMTHYQSLLLTERVSFAPPAiLNPAsLLPEadEAPaHkCEeILAEETGiRPDLTDQPwPGAmTWfTDGSSFvvEGKRKAGgAVVDGKaVIWASSLPEGTSAQKAELIALiQALRLAEGraLNVYTDSRYAFATAHVHGAIYRhRGLLTSAGKDIKNKEEILsLLEAvHLPRRVAIIHCPGHQKgtGPvEKGNqMADqEAKKAAqgpmtLvvRTqQPaaeEinkrTLTEeEGrdYLaniHHLTHLGtkKLLkLVSkSPYYIPgLkgiVeEIvknCRACAlTNAGSsRlQeGKRlRGDRPGaYWEtDFTEVKPaRYGNKYLLVFIDTFSGWVEAFPTKKETAnVVvKKILEEILPRFGIPKVmGSDNGPAFVSQVSQGLArQLGtNWKLHCAYRPQSSGQVERMNRTlKETLTKiALEsGGsDWTavLPyALfRVRNTPGPlGLTPFElmYGAPPPIFmtvGDknrPDVsFSPssSLlArLKALEIVRkEVWeQLKEtYvagDTqVPHqFeVGDaVLVRRHRagNLEPRWKGPYLVLLTTPTAVKVeGIptWVHASHVKrAPPgvShDEWtLEKTtNPfKLRLlRRSdpkrlqppqscstnvggtq

Env:

MSGLWrRLLILLScaCFVGAiPKdFNPHsPVqQTWEVLNeGGrAVWTiAEvHPLWTWWPDLfPDiCkLAigAPPGWDLEGYSDiqRAPlTPPPYvEKHSRDPwGgCSnqRDRSMLRTHpFYVCPGPHRSqSLnPTCGgKaDFfCKsWGCETsGTArWKPSSSWDyIrVTanYsLAsyVpGgfDLDecTdWChPLRvTFTEPGKRalgWTKGYtWGLRiykerYDEGlLFTIRLKIeTpyNpLGPPTKFTPlTHtiTQPTPViAdPLNmAAITQPPTpqVPLTITPAiPsRqRmFNLVrGAFyALNrTdPSATEDCWLCLSsGPPYYEGIAFnGDFNRTSShTsCSWGTgQKLTLTEVSARNpGLCIGtPPSTHkHLCgQIQSVSRTeaNYyLVPSpvGWWACNTGLTPCvSTKVFnSShDFCVmIQLlpRVYYHPASSLEESyagRRSKREpITLTLAAfmGIGmAVGVGTGvSALIeGrQGIQSLrdAVNeDLaAIEKSIDaLEKSLTSLSEVVLQNRRGLDLLFLKEGGLCAALKEECCFYADHTGIVRDSMqKLRERLERRkRErDAQrGWfESWFESRpSWiTSLISaVAGPILmICLaLvFGPCIINRgmAFIQSKIdTVKLMVLQrQYQPIVQVDeelgdtnl

Consensus amino acid sequences for group 3:

Gag:

MGQTVTTPLSLTLqHWgDVqRIAsNQSVDVRKRRWITFCSAEWPTFNVGWPqDGTFNLSIISQVkSRVFcPGPHGHPDQVPYIVTWEALAyDPPPWVKPFVSPKLPPLPtAPVLPPgPSAQPPSRSALYPALTPSikSKPPKPQVLPDSGGPLIDLLTEDPPPYGAqPSSsArEnDEEEAATTSEvSPPSPmVSRLRGrRdPPAADSTTSQAFPLRMGGdGQLQYWPFSSSDLYNWKNNNPSFSEDPGKLTALIESVLITHQPTWDDCQQLLGTLLTGEEKQRVLLEARKAVRGNDGRPTQLPNEVNAAFPLERPDWDyTTTEGRNHLVLYRQLLLAGLQNAGRSPTNLAKVKGITQGPNESPSAFLERLKEAYRRYTPYDPEDPGQETNVSMSFIWQSAPDIGRKLERLEDLKsKTLGDLVREAEKIFNKRETPEEREERIRRETEEKEERRRaEDEQrEKERDRRRHREMSKLLATVVIGQRQDRQGGERRRPQLDKDQCAYCKEKGHWAKDCPKKPRGPRGPRPQTSLLTLGD

Pro:

GGQGQEPPPEPRITLKVGGQPVTFLVDTGAQHSVLTQNPGPLSDKsAWVQGATGGKRYRWTTDRKVHLATGKVTHSFLHVPDCPYPLLGRDLLTKLKAQIHFEGSG

Pol:

AQVVGPMGQPLQVLTLNIEDEYRLHETSKEPDVSLGSTWLSDFPQAWAETGGMGLAVRQAPLIIPLKATSTPVSIKQYPMSQEARLGIKPHIQRLLDQGILVPCQSPWNTPLLPVKKPGTNDYRPVQDLREVNKRVEDIHPTVPNPYNLLSGLPPSHQWYTVLDLKDAFFCLRLHPTSQPLFAFEWRDPEMGISGQLTWTRLPQGFKNSPTLFDEALHRDLADFRIQHPDLILLQYVDDLLLAATSELDCQQGTRALLQTLGDLGYRASAKKAQICQKQVKYLGYLLKEGQRWLTEARKETVMGQPTPKTPRQLREFLGTAGFCRLWIPGFAEMAAPLYPLTKTGTLFNWGPDQQKAYQEIKQALLTAPALGLPDLTKPFELFVDEKQGYAKGVLTQKLGPWRRPVAYLSKKLDPVAAGWPPCLRMVAAIAVLTKDAGKLnMGQPLVILAPHAVEALVKQPPDRWLSNARMTHYQAMLLDTDRVQFGPVVaLNPATLLPLPGKePhHDCLEILAEtHGTRPDLTDQPLPDADHTWYTDGSSFLQEGQRKAGAAVTTETEVIWAKALPAGTSAQRAELIALTQALKMAEGKKLNVYTDSRYAFATAHVHGEIYRRRGLLTSEGKEIKNKgEILALLKALFLPKRLSIIHCPGHQKGNSAEAKGNRMADQAAREAAmgTdTkaSSLLIEtSTPYTPdFfHYTeTDIknLQELGATYDreKKyWVlQGKPVMPDQFTFELLDFLHQLTHLSyqKMrALLdRkESPYYMLNkDkiLhEVaESCQACvQVNASKaKiGaGVRVRGHRPGTHWEIDFTEVKPGLYGYKYLLVFVDTFSGWVEAFPTKHETAKVVTKKLLEEIFPRFGMPQVLGTDNGPAFVSQVSQSVAkLLGIDWKLHCAYRPQSSGQVERMNRTIKETLTKLTLATGtRDWVLLLPLALYRARNTPGPHGLTPYEILYGAPPPLVNFhDPeMSKFTnSPSLQAHLQALQaVQrEVWkPLAAAYQdQLDqPVIPHPFRVGDTVWVRRHQTKNLEPRWKGPYTVLLTTPTALKVDGIAAWIHAAHVKAATTppagtaSgPTWKVQRSQNPLKIRLTRGApl

Env:

MeGPAfSKPLKdKiNPWGPPIvLgILiRAGVSvQHDSPHQVfNVTWrVTNlMTGQTANATsLLGTmTDAFpkLYfdLCDLIGDDWDETGLGCRTPGGRKRARTfdFYVCPGHTVPtGCGGPrEgYCgKWGCETTGqAYWKPSSSWDLISLKrgNtPrNQGPCydSsaVSSGikGatpGgrCNPLVLeFTDAGKKaSWdGpKvWGLRLyRStGtDPVTrfsLTrQVLNiGPRVPIGPNPVitDQlPPsRpVQImLPrPPqPPPPgAASIVPeTApPSQQPGTGDRLLNLVDGAYQALNLTSPDKTQECWLCLVSgPPYYEGVAVLGTYSNHTSAPANCSVASQHKLTLSEVTGQGLCVGAVPKTHQALCNTTQkTSdGSYYLaAPAGTiWACNTGLTPCLSTTVLdLTTDYCVLVELWPkVTYHSPgYVYGqFErKTKYKREPVSLTLALLLGGLTMGGIAAGVGTGTTALvATkQFeQLQAAIhtDLgALEKSVSaLEKSLTSLSEVVLQNRRGLDLLFLKEGGLCAALKEECCFYADHTGvVRDSMAKLRERLNQRQKLFESGQGWFEGLFNRSPWFTTLISTIMGPLIILLLILLFGPCILNRLVQFVKDRISVVqalvltqqyhqlksidpeevesre
