# Supplementary material for: Murine Gammaretrovirus Group G3 Was Not Found in Swedish Patients with Myalgic Encephalomyelitis/Chronic Fatigue Syndrome and Fibromyalgia
Source: PLoS One. 2011 Oct 12;6(10):e24602. doi: 10.1371/journal.pone.0024602 (PMC3192035; doi:10.1371/journal.pone.0024602)
Supplement: Information S4 — Development of the gag and env RTQPCR; Evolutionary conservation of target sequences. (PDF) [file pone.0024602.s005.pdf]

# Supplemental information S5

## Design of two RTQPCRs for XMRV like murine leukemia viruses

### Identification of conserved portions of MLV-like retroviral genomes using the ConSort© program

The variation in MLV *gag* genes was studied by blasting the nr portion of GenBank with *gag* of XMRV VP62. BLASTN retrieved 109 MLV *gag* sequences. The result was treated with the variation analysis program ConSort (Blomberg et al, unpublished) (fig S1 A and B). The selected portion of *gag* corresponded approximately to the major homology region of Gag.

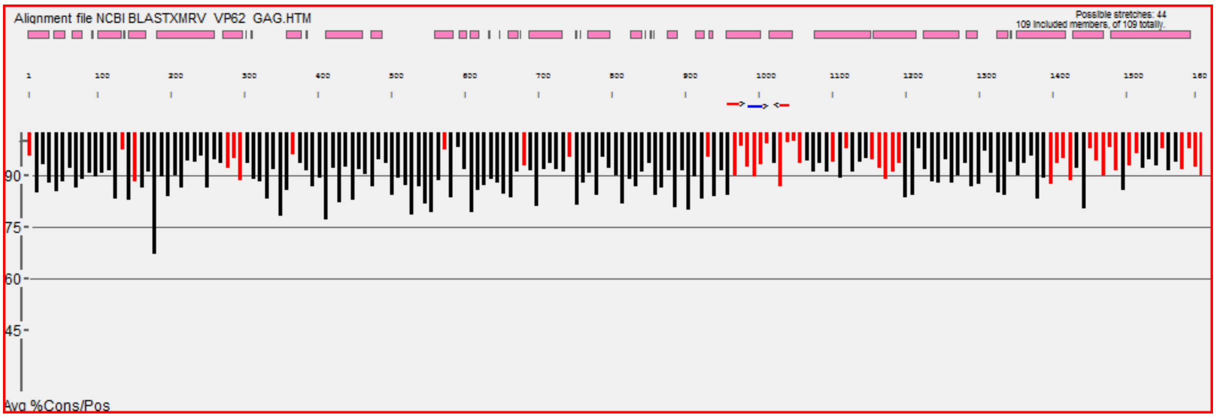


**Figure S5** A. ConSort representation of variation in the entirety of MLV-like *gag* sequences. The selected *gag* primers and probe are shown above the variation histogram. Red bars indicate sequence portions containing at least one invariant nucleotide position.

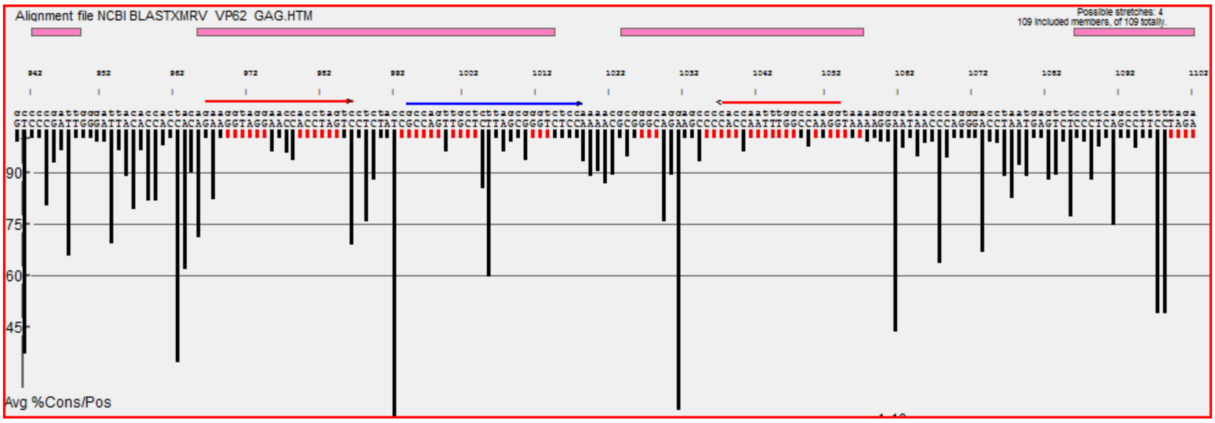


**Figure S5.** B. A close-up view of the ConSort representation of the *gag* portion selected for primer and probe construction. ConSort of entire MLV-like *gag* sequences. The mauve rectangles at the top indicate regions suggested as primer or probe targets by ConSort.

The selected primers and probe were tested in a QRTPCR against the XMRV VP62 plasmid kindly provided by prof Robert Silverman. Sensitivities of 1-10 copies per PCR reaction were obtained (Figure S5 C and D).

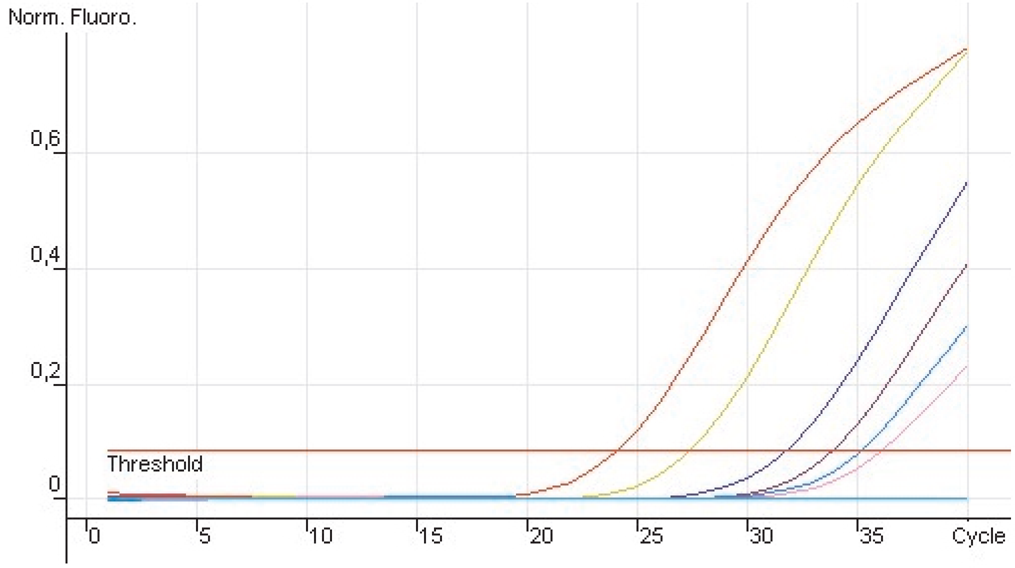


**Figure S5. C. Real time amplification from a dilution series of VP62 plasmid DNA.**

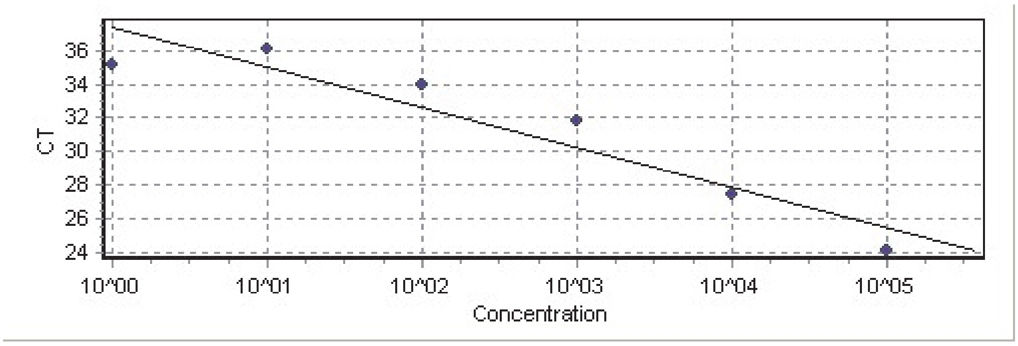


**Figure S5. D. Plot of number of copies per PCR reaction versus Ct value, same experiment as above.**

Similarly, variation in the MLV *env* gene was studied by retrieving sequences similar to XMRV VP62 env with BLASTN of the nr portion of GenBank. The primers and probe in the XMRV envelope gene (ConSort) were based on 484 MLV *env* sequences (Fig S5 E, F and G).

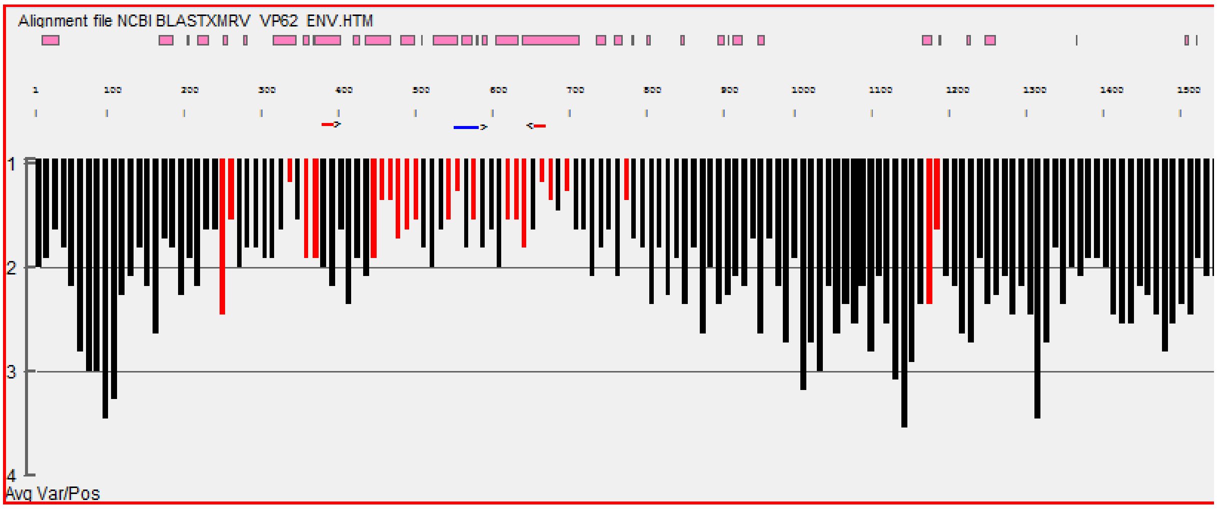


**Figure S5.** E. ConSort representation of variation in the entirety of MLV-like *env* sequences. The selected *env* primers and probe are shown above the variation histogram. Other conventions are as in figure S5A.

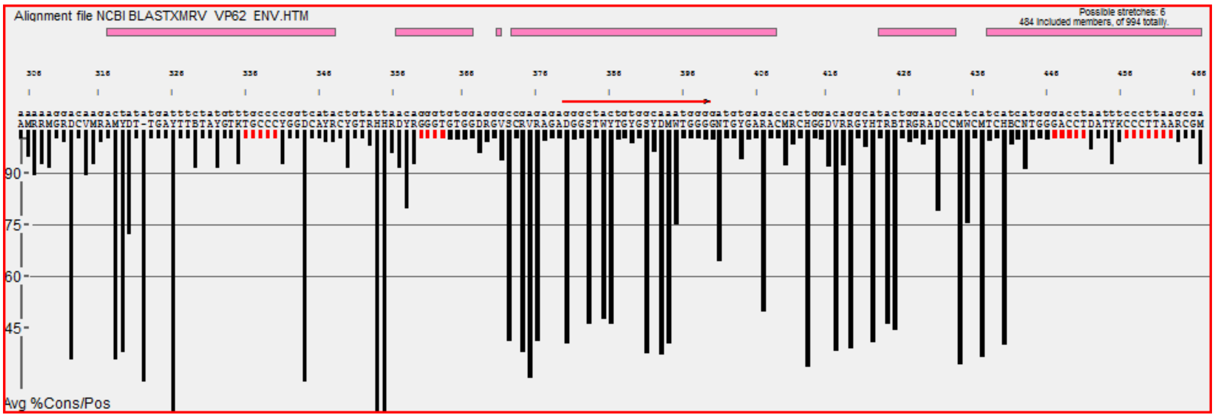


**Figure S5.** F. A close-up view of the variation in the target area for the forward primer. Conventions are as in figure S5A.

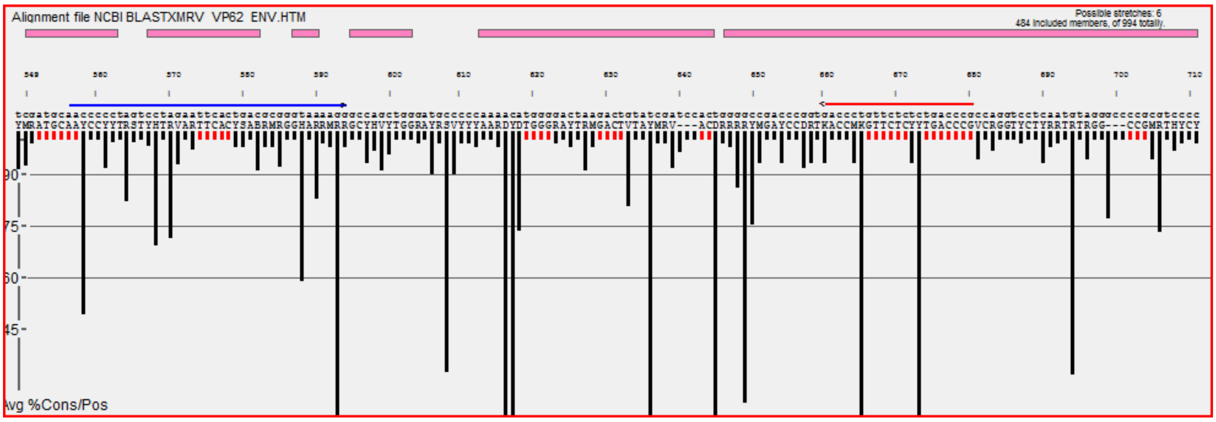


**Figure S5.** G. A close-up view of the variation in the target area for the probe and the reverse primer. Conventions are as in figure S1.

In an attempt to study the variation of a restricted set of target sequences, most highly similar to XMRV, the BLASTN hits were restricted to those with score >1000, and coming from a Xenotropic or a Polytropic MLV (60 hits) (Figure S5 H, I and J).

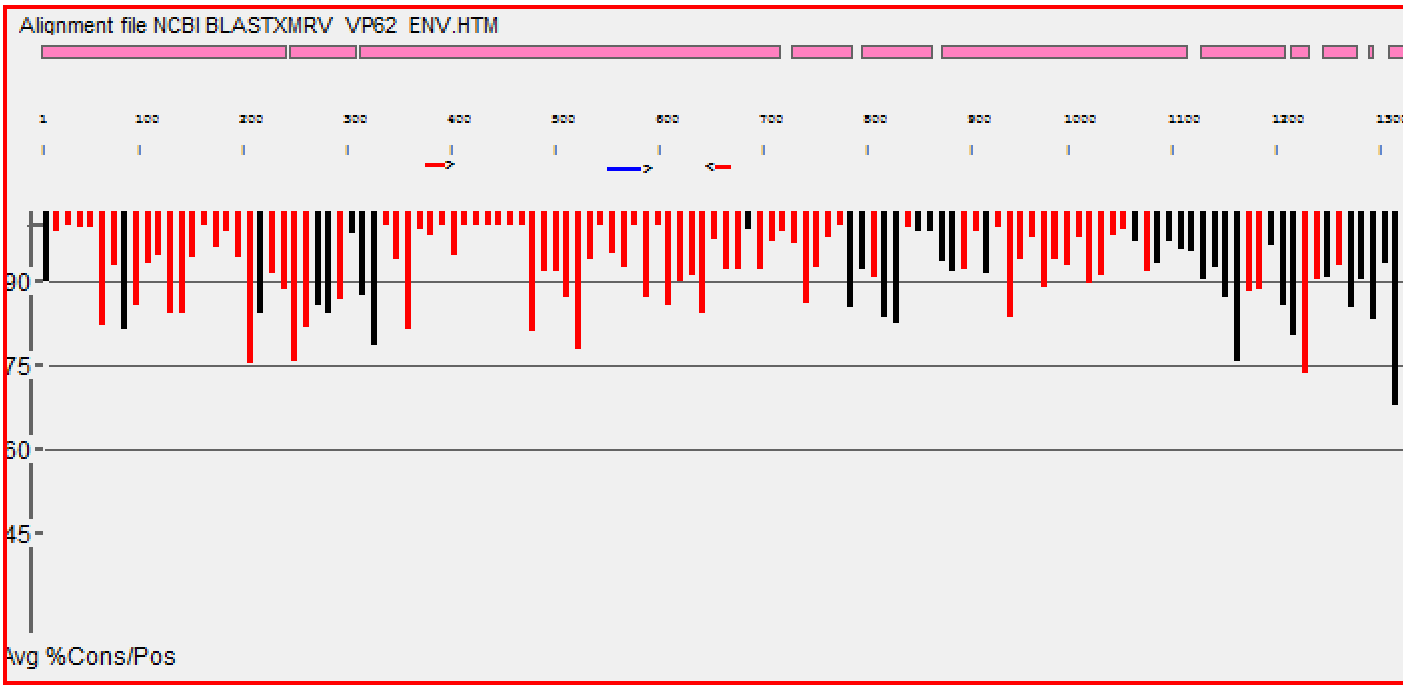


**Figure S5.** H. ConSort representation of variation in a restricted set of highly XMRV-related sequences. The variation is smaller than in the set depicted in Figure S5 C.

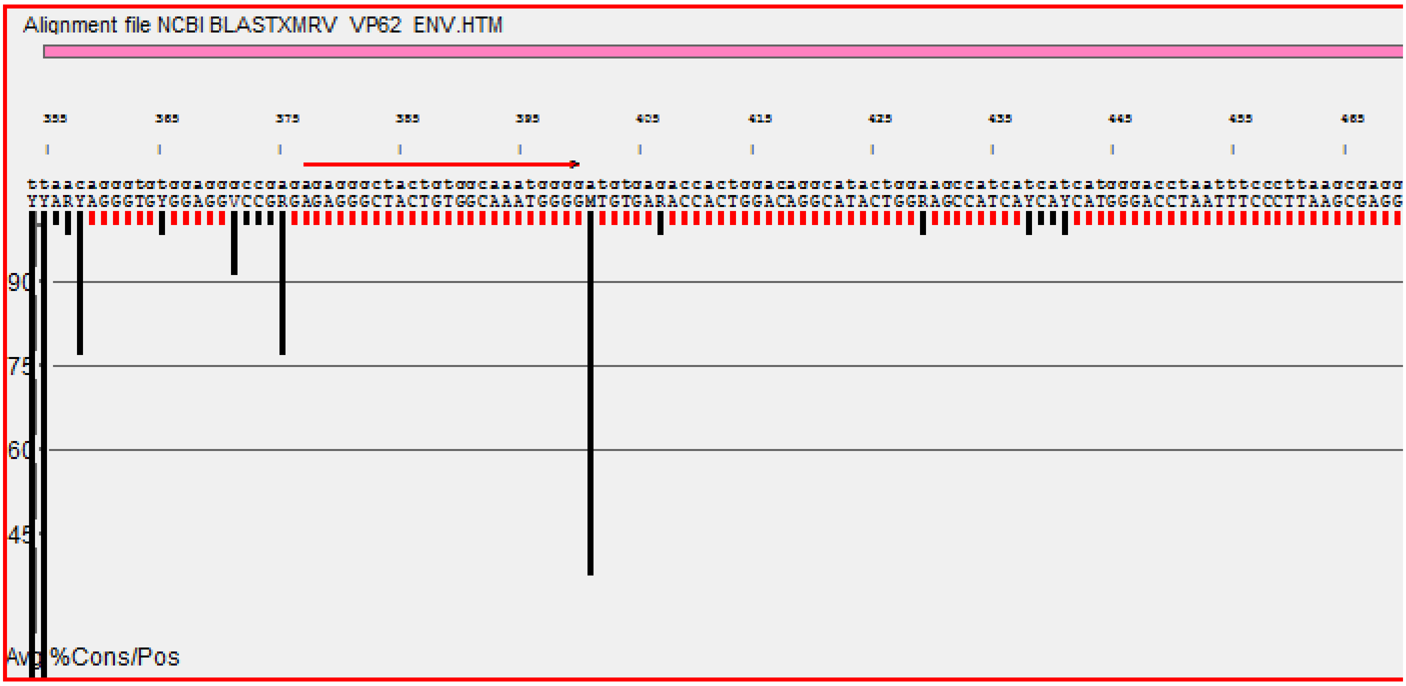


**Figure S5.** I. Close -up of the target region for the forward primer, in a restricted set of highly XMRV - related sequences.

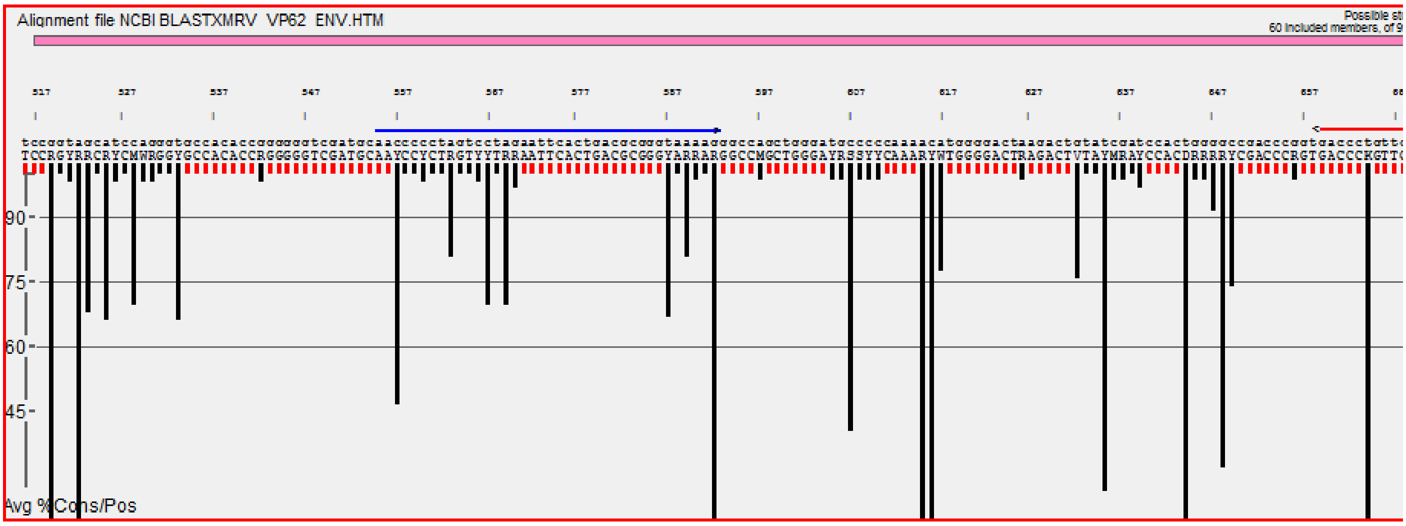


**Figure S5.** J. Close -up of the target region for the probe and reverse primer, in a restricted set of highly XMRV-related sequences.

The probe was constructed according to the MegaBeacon principle (Muradrasoli et al, 2009). This allows a greater variation tolerance than ordinary nuclease-activated probes. Probes of greater length can tolerate more mismatches than probes of shorter length. The chosen MegaBeacon probe had a length of 49 nucleotides, of which 39 were target specific. More details regarding the properties of MegaBeacon probes are given in (Muradrasoli et al, 2010; see reference in the main paper).

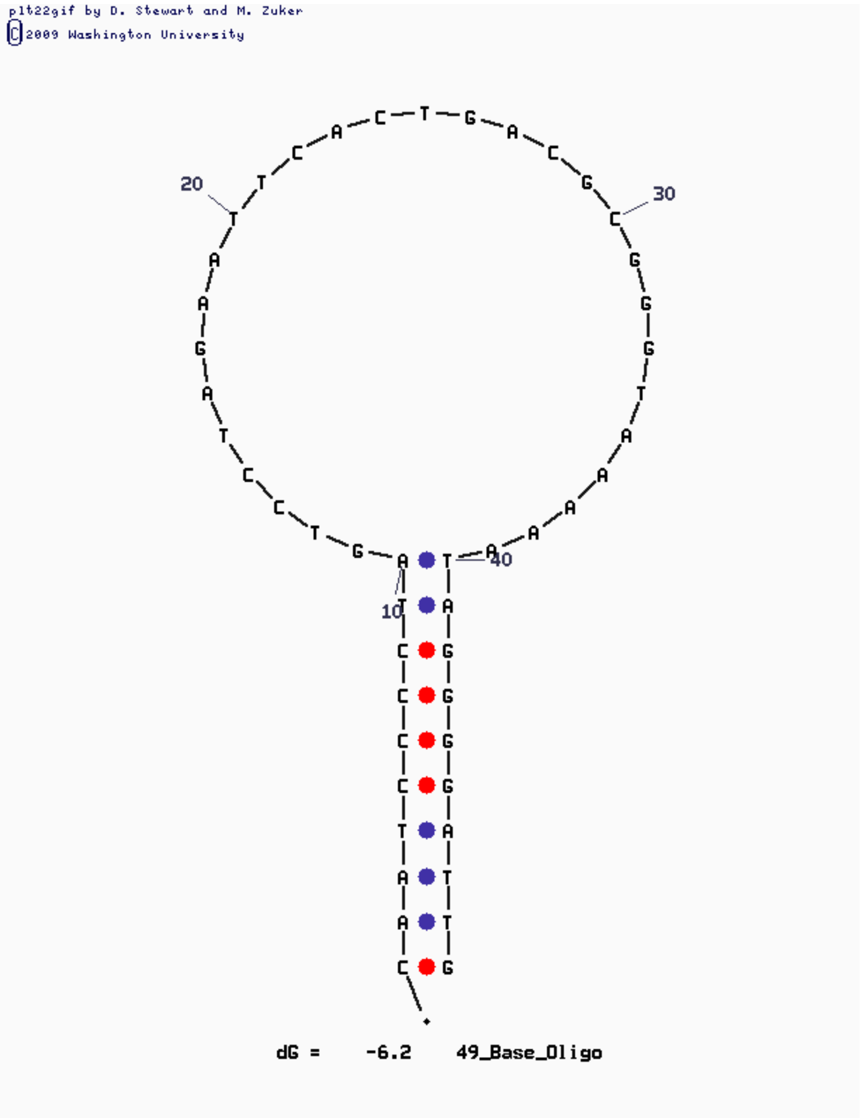


**Figure S5 K.** The MegaBeacon probe for XMRV *env*. The figure was obtained from the M. Zuker MFOLD home page at Washington University.
